# Chlorinated Enyne Fatty Acid Amides from a Marine Cyanobacterium: Discovery of Taveuniamides L-M and Pharmacological Characterization of Taveuniamide F as a GPCR Antagonist with CNR1 Selectivity

**DOI:** 10.3390/md22010028

**Published:** 2023-12-30

**Authors:** Lobna A. Elsadek, Emma K. Ellis, Gustavo Seabra, Valerie J. Paul, Hendrik Luesch

**Affiliations:** 1 Department of Medicinal Chemistry, University of Florida, 1345 Center Drive, Gainesville, FL 32610, USA; lobna.elsadek@ufl.edu (L.A.E.); downing.ekate@ufl.edu (E.K.E.); seabra@cop.ufl.edu (G.S.); 2 Center for Natural Products, Drug Discovery and Development (CNPD3), 1345 Center Drive, University of Florida, Gainesville, FL 32610, USA; 3 Smithsonian Marine Station, 701 Seaway Drive, Fort Pierce, FL 34949, USA; paul@si.edu

**Keywords:** fatty acid amides, chlorinated enyne, GPCR, CNR1, cyanobacteria

## Abstract

NMR and MS/MS-based metabolomics of a cyanobacterial extract from Piti Bomb Holes, Guam, indicated the presence of unique enyne-containing halogenated fatty acid amides. We isolated three new compounds of this class, taveuniamides L-N (**1**–**3**)**,** along with the previously reported taveuniamide F (**4**), which was the most abundant analog. The planar structures of the new compounds were established using 1D and 2D NMR as well as mass spectrometry. We established the configuration of this chemical class to be *R* at C-8 via Mosher’s analysis of **4** after reduction of the carboxamide group. Our biological investigations with **4** revealed that the compound binds to the cannabinoid receptor CNR1, acting as an antagonist/inverse agonist in the canonical G-protein signaling pathways. In selectivity profiling against 168 GPCR targets using the β-arrestin functional assay, we found that **4** antagonizes GPR119, NPSR1b, CCR9, CHRM4, GPR120, HTR2A, and GPR103, in addition to CNR1. Interestingly, **4** showed a 6.8-fold selectivity for CNR1 over CNR2. The binding mode of **4** to CNR1 was investigated using docking and molecular dynamics simulations with both natural and unnatural stereoisomers, revealing important CNR1 residues for the interaction and also providing a possible reasoning for the observed CNR1/CNR2 selectivity.

## 1. Introduction

Fatty acid amides are a broad class of compounds present in humans and other organisms [1]. Because many of these molecules are involved in several physiological effects, primarily through cell signaling, fatty acid amides offer a promising prospect for the development of novel medications to treat sleep disorders, anxiety, depression, cardiovascular disease, neurodegenerative illnesses, bacterial, and fungal infections [2,3]. Consequently, identifying new lipid amides is of significant interest. Cyanobacteria have been a rich source of diverse structural classes, including fatty acid amides [4]. Examples of cyanobacterial fatty acid amides containing halogen atoms include pitiamides [5], janthielamide A [6], and kimbeamides [6], which are characterized by a terminal vinyl chloride. Credneramides [7], malyngamides [8], and grenadamides [9] contain an internal vinyl chloride moiety and the latter also have terminal vinyl chloride. Jamaicamide A harbors an internal vinyl chloride and an alkynyl bromide terminal unit [10]. Columbamides [11] carry internal and terminal chlorine within the alkyl chain, while semiplenamides [12], serinolamides [13], and parguerene [14] are examples of fatty acids that are not halogenated.

**Figure 1:**
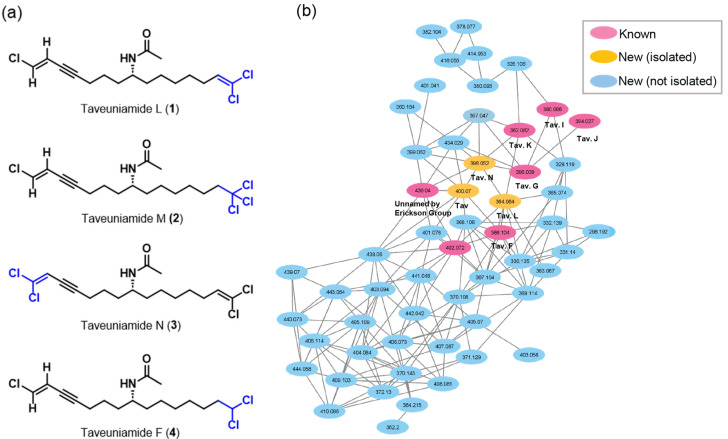
Metabolomics discovery of new and known compounds from marine cyanobacterium VPG14-26. (**a**) Chemical structures of isolated new taveuniamides L-N (**1**–**3**) and known taveunimade F (**4**) [15]. (**b**) GNPS generated network analysis using MS/MS data of the organic fraction from which **1**–**4** were purified.

Here, we report the discovery and pharmacological characterization of new halogenated enyne fatty acid amides, taveuniamide L-N (**1**–**3**), and taveuniamide F (**4**) [15], from a marine cyanobacterium collected from Piti Bomb Holes on Guam (Figure 1a).

To date, these lipid acetamides with halogenated enyne systems lack information about the absolute configuration at C-8 and have not been biologically characterized. We successfully assigned the configuration at the chiral center to be *R* after the reduction in the carboxamide group and Mosher’s analysis of **4**. Utilizing biochemical and cell-based functional studies, we discovered that **4** binds CNR1 with subsequent functional modulation through the G-protein dependent and β-arrestin pathway. Compound **4** regulated the function of other GPCR targets and, interestingly, retained an antagonism mode of action to the β-arrestin pathway in all hits revealed.

## 2. Results and Discussions

### 2.1. Isolation

HRLCMS/NMR-guided fractionation yielded a C18 column fraction (90% MeOH/H_2_O) with isotope clusters indicating that the fraction was rich in halogenated fatty acids. The ^1^H NMR spectrum showed several analogs closely related to the previously isolated halogenated unsaturated acetamides by the groups of Erickson [16] and Gerwick [15] (Figure S1). GNPS network analysis (Figure 1b) revealed the presence of taveuniamides (F, G, I, J, K), two unnamed compounds by the Erickson group [16], and several undescribed metabolites that fueled our efforts to follow up on this fraction. Since taveuniamide F (**4**) was abundant, we also purified the compound to use it as our model compound for the stereochemical determination and biological evaluation of this class of compounds.

### 2.2. Structure Elucidation

**Figure 2:**
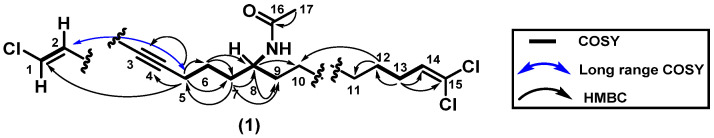
Key 2D NMR correlations for taveuniamide L (**1**).

The HRESIMS of taveuniamide L (**1**) showed three *m*/*z* signals separated by two mass units (364.0996, 366.0966, 368.0934) with relative abundances of 100%, 96%, and 30%, respectively, which matches the isotope distribution of three chlorines. Coupling the HRMS data with the analysis of ^1^H and 2D NMR (HSQC, COSY, HMBC; Table 1), we established the planar structure of **1** (Figure 2). HSQC showed a total of eight methylenes, one methyl, four methines, and one proton with no correlation indicating the presence of an exchangeable proton. From the HMBC, we observed four quaternary carbons. Aligning these data with the [M + H]^+^ of **1**, we defined the molecular formula to be C_17_H_24_ ^35^Cl_3_NO with five degrees of unsaturation. Two olefinic protons H-1 (δ_C_/δ_H_ 128.8/6.43) and H-2 (δ_C_/δ_H_ 114.0/5.90), showing COSY correlations to each other with a coupling constant of 13.6 Hz, suggested E double bond configuration. The relatively downfield chemical shift of H-1 (δ_H_ 6.43) compared to aliphatic alkene systems suggested that factors such as inductive effects or magnetic anisotropy are deshielding the vinylic proton. Therefore, we connected a chlorine atom to C-1, which is also supported by the smaller coupling constant through the alkene bond as attributed to inductive effects [17]. The methylene C-5 (δ_C_/δ_H_ 19.1/2.32) has HMBC correlations to two quaternary carbons at (δ_C_ 92.7, 76.12), suggesting it is linked to an acetylenic system. H-5 showed a strong COSY correlation to H-6 with a coupling constant of 7.66 Hz; therefore, it was linked to C-6 (δ_C_/δ_H_ 24.8/1.55). H-5 also showed a weak COSY signal to H-2 with a coupling constant of 2.32 Hz, suggesting a long-range coupling through the alkyne bond. This agrees with the ^5^*J*_H,H_ commonly seen with enyne systems as imposed by the planar acetylenic bond [17,18]. Thus, we attached C-5 to C-4 and C-3 to C-2, which explains the deshielding of C-3 (δ_C_ 92.7) that is due to the halogenated vinylic system. The spin system from C-6 to methine C-9 was connected using COSY correlations and extended to C-10 by HMBC correlations. H-8 is coupled to an exchangeable proton at (δ_H_ 5.07), which is assumed to be an amide proton since the chemical shifts (δ_C_/δ_H_ 48.7/3.93) of the methine at position 8 are diagnostic of an alpha methine to an amide bond. A singlet methyl at 1.98 ppm was attached to the amide carbonyl based on the HMBC correlation seen for H-17 to the carbonyl (δ_C_ 169.58). From the COSY spectrum, the methylene H-13 coupling partners were determined to be H-12 (δ_H_ 1.40) and the olefinic, H-14 (δ_H_ 5.83). Due to the overlapping protons at the methylene region (1.33–1.40), it was hard to draw conclusions about the connectivity of C-12, C-11 and C-10 from the COSY spectrum. However, relying on the HMBC correlations observed from H-12 and H-8, we confidently identified the linkage from C-12 to C-10. Being left with the HMBC correlation from H-13 to the quaternary olefinic carbon (δ_C_ 119.8) suggested it is a *gem*-dichloro-olefin in order to account for the missing two chlorine atoms and the degrees of unsaturation based on the molecular formula.

The analysis of taveuniamide M (**2**) HRESIMS at *m*/*z* 400.0757 in conjunction with the NMR data (Table 2) indicated that the molecular formula is C_17_H_25_^35^Cl_4_NO, which includes one extra proton, one chlorine, and a saturated double bond compared to **1**. A careful comparison of NMR data indicated an additional methylene in the HSQC of **2** at the expense of the C-14 vinylic methine in **1**. Furthermore, the C-13 (δ_C_/δ_H_ 26.2/1.76) and C-15 (δ_C_ 100.3) chemical shifts were significantly different from **1**. The methylene protons (δ_H_ 2.66) showed HMBC correlations to C-13 and the quaternary carbon C-15 that is highly downfield (δ_C_ 100.3), indicative of a trichloro substitution at C-15 [17]. With this, we have accounted for differences in the molecular formulas between **2** and **1**, which are fulfilled by the terminal trichloromethyl in **2** relative to the *gem*-dichloro olefin in **1**. Looking closely at the NMR data, we assigned all the protons and carbons and connected the scaffold based on the COSY and HMBC correlations (Table 2).

The molecular formula of taveuniamide N (**3**), C_17_H_23_^35^Cl_4_NO, with five degrees of unsaturation, was established by HRESIMS at *m*/*z* 398.0600 (for [M + H]^+^) in combination with its NMR data (Table 3). NMR analysis showed that **3** is identical to **1** except that the vinylic proton at 6.43 ppm was not present, and from the HMBC, an additional quaternary carbon (δ_C_ 130.1) appeared. This suggested that the enyne system in **3** is dichlorinated at C-1. This is also supported by the multiplicity of H-2 indicating t instead of dt compared to the H-2 in **1**, in addition to the remarkable variations in the chemical shifts of C2–4 (∆ δ_C_ −3.0, +6.1, −1.2, respectively), as influenced by the anisotropic and/or inductive effects of the additional chlorine atom.

The molecular formula of (**4**) was established as C_17_H_26_ ^35^Cl_3_NO by HRESIMS, which matches the published taveuniamide F. NMR data and optical rotation were in complete agreement with the reported data and thus confirmed that **4** is taveuniamide F.

**Figure 3:**
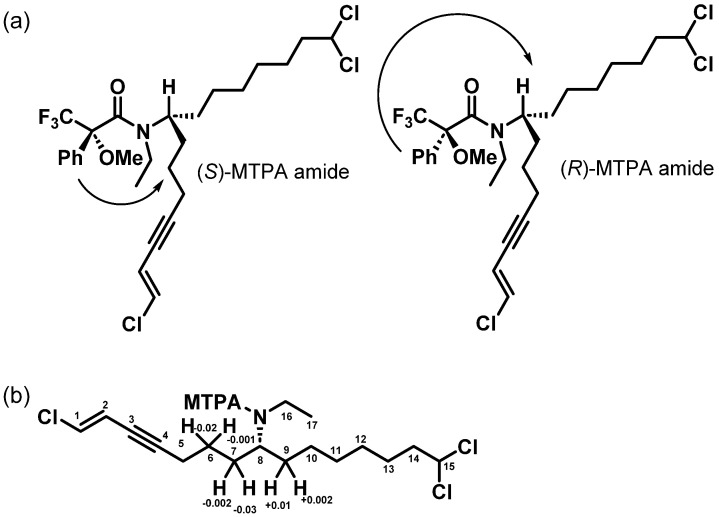
Mosher’s (tertiary amide) analysis of the reduced form of taveuniamide F (**4**). (**a**) The deshielding effect of the phenyl group of the MTPA on the corresponding side chains of the stereogenic center. The *Z*-amide configuration is depicted since it is determined to be the more stable isomer based on DFT calculations. (**b**) ∆δ^S-R^ defining the configuration of taveuniamide F (**4**) as *R*.

All new analogs (**1**, **2**, **3**) and taveuniamide F (**4**) share a negative optical rotation, which indicates they have the same absolute configuration at C-8. Several attempts have been made to determine the absolute configuration at C-8, which was not previously defined in reported analogs. First, we attempted to crystallize these pure compounds but could not obtain a definite 3D crystalline shape optimal for X-ray diffraction. Second, since a conjugated enyne system is present at the terminus of the fatty acyl chain, we collected chiral dichroism (CD) data; however, no obvious Cotton effect resulted from chromophore interaction, primarily as it is far from the stereogenic center and due to the conformational flexibility. Third, we attempted to hydrolyze the amide functionality placed at the stereogenic center to perform Mosher’s analysis, as the phenolic group from the MTPA invokes a deshielding effect on the side chain protons facing the same side (Figure 3a). While standard deacetylation by acid hydrolysis failed to liberate the desired primary amine, using Cp_2_ZrHCl, the carboxamide group of **4** was reduced to the secondary amine [19]. The following amidation using (*S*)- and (*R*)-MTPA reagents resulted in the desired (*R*)- and (*S*)-MTPA amide, respectively (Figure 3a). The configuration was then elucidated using a combination of ^1^H NMR and 1D TOCSY analysis of both diastereomers to pinpoint which side chain at the chiral center is experiencing deshielding effects by the phenyl group. However, the configuration of the amide bond introduced a conformational question that would change the electronic environment. Therefore, we investigated the geometrical configuration of the amide bond using Density Functional Theory (DFT) calculations. Conformational searches starting from 4 conformers (*R* and *S* configuration of the C-8, and *Z* or *E* configuration of the amide bond) resulted in the *Z* configuration being the most thermodynamically stable in all cases (Figure S24), which aligns with the literature reported for the tertiary MTPA amides [20]. A thorough dihedral angle scan shows a *Z* configuration at least 5 kcal/mol more stable than the *E* configuration in all cases, and including solvent effects did not change the result (Figure S24). Based on the model generated and the analysis of the proton chemical shifts of both diastereomeric derivatives (Figure 3a,b), we concluded that the stereochemistry at C-8 is *R.*

### 2.3. Biological Characterization

#### 2.3.1. Cannabinoid CNR1 Receptor Binding

**Figure 4:**
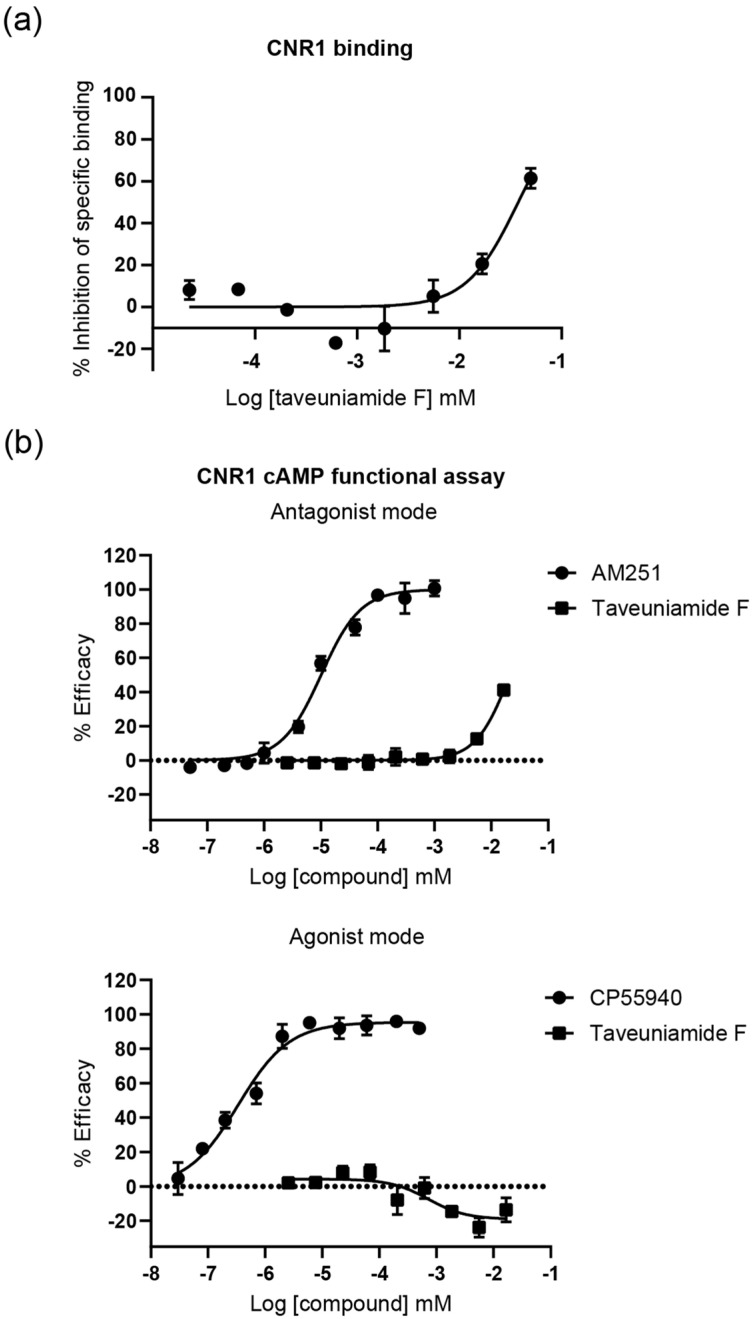
Taveuniamide F (**4**) binding and functional response characterization against CNR1. (**a**) Competitive radioligand binding assay of **4** to CNR1. Membrane homogenate of rat hematopoietic Chem-1 cells expressing human recombinant CNR1 receptors is incubated with 2 nM [3H]SR141716A for 60 min at 37 °C. Non-specific binding is estimated in the presence of 10 μM CP 55,940 (*K*_i_ = 0.025 μM; historical value at Eurofins Panlabs = 0.03 μM). (**b**) Effect of **4** in CNR1 cAMP functional assay in agonist and antagonist mode. Experiments were carried out as technical duplicates. Data are presented as mean ± SD (*n* = 2).

Endogenous fatty acid amides, such as anandamide and palmitoylethanolamide, affect numerous types of G protein-coupled receptors (GPCRs), including cannabinoid receptors (CNR1 and CNR2), transient receptor potential vanilloid type 1 (TRPV1), and peroxisome proliferator-activated receptors (PPARs) [2]. Since structurally related fatty acid amides isolated from cyanobacteria like columbamides A and B, serinolamide, semiplenamides, and mooreamide A showed cannabinomimetic effects [4], we tested **4** in a competitive radioligand binding assay against human CNR1. It appeared that **4** has a moderate binding affinity to CNR1 (Ki = 34 μM) (Figure 4a).

#### 2.3.2. CNR1 Functional Response Induced by Taveunimide F (**4**) through G-Protein-Dependent Pathway

To define the mode of activity, we examined the functional modulation of CNR1 by taveuniamide F (**4**) through the canonical cAMP pathway. Upon activation, CNR1 initiates signal transduction in a G-protein-dependent manner, primarily through Gαi/o [21,22]. This leads to adenyl cyclase (AC) inhibition and a consequent reduction in cAMP production, thereby suppressing protein kinase A (PKA)-mediated signaling cascades. Consequently, we evaluated **4** in the CNR1 cAMP functional assay (Figure 4b). In the antagonist mode, **4** inhibited CNR1-agonist-induced G-protein-dependent cAMP downregulation (IC_50_ = 21 µM). In the agonist mode, it suppressed G-protein-dependent cAMP downregulation. Hence, our findings indicate that compound **4** acts as an antagonist/inverse agonist, inhibiting CNR1 G-protein-dependent signal transduction.

#### 2.3.3. GPCR Target Selectivity Profiling and CNR1 Functional Selectivity Assessment 

In line with typical GPCRs, upon the phosphorylation of CNR1 by GPCR kinases (GRK), β-arrestin is recruited [21,22]. This facilitates receptor desensitization and endocytosis, with bound β-arrestin also enabling G-protein-independent signal transduction [21,22]. Promising therapeutic potential is observed for CNR1 ligands in neurological and metabolic disorders. However, the advancement of CNR1-targeted pharmacotherapeutics is impeded by concerns about adverse effects, rapid tolerance, and abuse potential [22]. Innovative drug discovery initiatives are investigating biased signaling to address these concerns while preserving these therapeutic benefits [22,23], as demonstrated by μ-opioid agonists that avoid recruiting β-arrestin-2 to the receptor [24]. This results in the absence of receptor internalization and the development of tolerance along with a reduction in respiratory depression.

**Figure 5:**
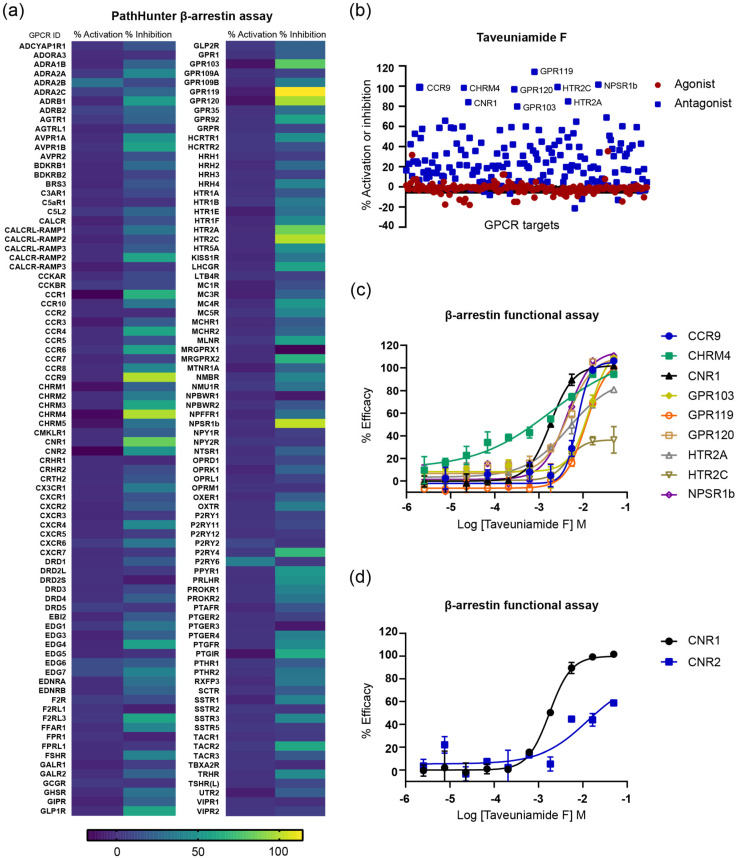
GPCR profiling of taveuniamide F (**4**) using cell-based functional screen (β-arrestin pathway). (**a**) Heatmap showing the screening of **4** (20 μM) against 168 GPCR targets in agonist (% activation) and antagonist mode (% inhibition). (**b**) Scatter plot of targets screened at 20 μM of **4**. Hits identified in the screen with ≥75% activity or inhibitions are labeled. (**c**) Dose–response curve of **4** against GPCR hits identified in the PathHunter β-arrestin primary screen. (**d**) Dose–response curve of **4** against CNR1 and CNR2. The experiments were carried out as technical duplicates. Data are presented as mean ± SD (*n* = 2).

Our aim was to broaden our comprehension of taveuniamide F’s (**4**) CNR1-mediated signaling pathways and investigate its functional selectivity. In addition, to unveil additional GPCR targets and a selectivity fingerprint, we screened **4** against a panel of 168 GPCRs (agonist, antagonist) at 20 μM final concentration in cell-based functional assays using PathHunter β-arrestin assay technology (Figure 5a). Interestingly, **4** did not show any significant agonist response, but displayed antagonistic activities (Figure 5a,b). Nine receptors were recognized as significant hits (% inhibition ≥ 80%, Figure 5b), including CNR1. To validate the hits (GPR119, NPSR1b, HTR2C, CCR9, CHRM4, GPR120, HTR2A, GPR103, CNR1) as targets, we tested **4** against them in a dose–response format using 10-point concentrations (Figure 5c, Table 4). All hits were validated except HTR2C (IC_50_ > 50 μM, Figure 5c, Table 4). Selectivity was observed in HTR2A (IC_50_ 4.8 μM) over HTR2C (Table 4). The two isoforms of the metabotropic serotonin 5-HT2 receptor—HTR2A and HTR2C—share 50% of their overall sequence homology and 80% within their seven transmembrane domains [25]. They are primarily localized in the central nervous system (CNS) and are involved in a variety of behavioral and physiological processes (e.g., appetite, sleep, and endocrine secretion), whereas their dysregulation results in neuropsychiatric diseases (e.g., addiction, obesity, schizophrenia) [25]. GPR119 (IC_50_ 11.7 μM, Table 4) is activated by glucagon-like peptide-1 (GLP-1), as well as the fatty acid derivative oleoylethanolamide (OEA), and is hence described as a novel cannabinoid receptor [26]. It is an emerging therapeutic target in type 2 diabetes and obesity, with further characterization of its pathophysiological roles needed [27]. GPR120 (IC_50_ 4.7 μM, Table 4) is referred to as a free fatty acid receptor 4 (FFAR4) and is responsible for regulating inflammatory and metabolic processes [27,28]. So far, there have been four FFARs identified that function as receptors for free fatty acids, categorized according to their chain length. FFAR1 (GPR40) and FFAR4 (GPR120) are triggered by long-chain fatty acids such as palmitate, oleate, and linoleate. On the other hand, FFAR2 (GPR43) and FFAR3 (GPR41) are primarily activated by short-chain fatty acids such as acetate, butyrate, and propionate. It was reasonable to find that GPR120 activity is influenced by **4** due to its status as a long-chain fatty acid with an aliphatic tail of more than 12 carbons. Nonetheless, the GPCRs panel tested does not encompass the aforementioned FFARs, which would have been necessary to assess the specificity of **4** for these receptors. GPR103 (IC_50_ 14 μM, Table 4) is activated by the endogenous neuropeptide ligands, QRFP26 and the N-terminally extended QRFP43 [29]. It is believed to be involved in glucose homeostasis and appetite regulation [29,30]. NPSR1b (IC_50_ 4.4 μM, Table 4) is a receptor for neuropeptide S [31]. Inflammatory bowel illness, panic disorders, rheumatoid arthritis, and asthma susceptibility have all been linked to polymorphisms in the gene encoding NPSR1b [31]. NPSR1b is thought to be a target for endometriosis, a chronic inflammatory condition causing pelvic pain and infertility in women [32]. CCR9 (IC_50_ 7.4 μM, Table 4) is a chemokine receptor that is activated by CCL25 and has been found to be overexpressed in rheumatoid arthritis, colitis, type 2 diabetes, and different malignancies [33]. The antagonism of CHRM4, a type of muscarinic acetylcholine receptor (IC_50_ 1.9 μM, Table 4), can have effects on various physiological processes, including heart rate, smooth muscle contraction, and neurotransmission, depending on the specific location and function of the receptor [34].

Taveuniamide F (**4**) was most potent against CNR1 (IC_50_ 1.84 μM), which prompted us to assess the selectivity for CNR1 vs. CNR2. Both receptors are distributed differently and result in distinct physiological effects. The CNR1 receptor is prevalent in the brain, with significant expression in the basal ganglia nuclei, hippocampus, cortex, and cerebellum [35]. This receptor’s location within the central nervous system coincides with its involvement in controlling motor function, cognition and memory, and analgesia [35]. It is typically found at the terminals of central and peripheral neurons, where it mediates neurotransmitter release [35]. The CNR2 receptor is extensively expressed in peripheral immune organs such as macrophages, spleen, tonsils, thymus, and leukocytes, as well as the lung and testes [35]. In an antagonist screen (β-arrestin), we compared the targeting of **4** to CNR1 and CNR2 receptors. We found that **4** is 6.8-fold more selective to CNR1 (Figure 5d).

Overall, the CNR1 cellular activity of **4** in the cAMP functional assay ((IC_50_ = 21 µM) correlates to the CNR1 binding data (IC_50_ 37.7 μM). The inhibition of agonist-induced β-arrestin recruitment by **4** at IC_50_ of 1.84 μM might suggest bias toward β-arrestin signaling. However, it is important to note that the current assay setup does not consider the kinetics of these processes [36]. Thus, further controlled studies are needed to address the functional selectivity of GPCR ligands.

### 2.4. Assessment of Taveuniamide F (***4***) Cytotoxicity in HEK293 Cells

To evaluate the potential safety of **4** and verify the authenticity of the observed effects in the cell-based GPCR assays, we evaluated its impact on the viability of HEK293 kidney embryonic cells. The concentrations tested encompassed a three-fold dilution series, with the highest concentration set at 100 μM. After a 24 h exposure period, the majority of tested concentrations exhibited negligible effects on cell viability, as indicated by a consistent 100% cell viability (Figure S26). Only at 100 μM was a reduction in cell viability of 56% recorded (Figure S26). These findings validate that the concentrations effective in both the cAMP functional assay and the β-arrestin recruitment assay do not elicit toxic effects on the cells and suggest a favorable safety margin within cellular systems.

### 2.5. Molecular Docking and Molecular Dynamic Simulations of Taveuniamide F (***4***) into CNR1

**Figure 6:**
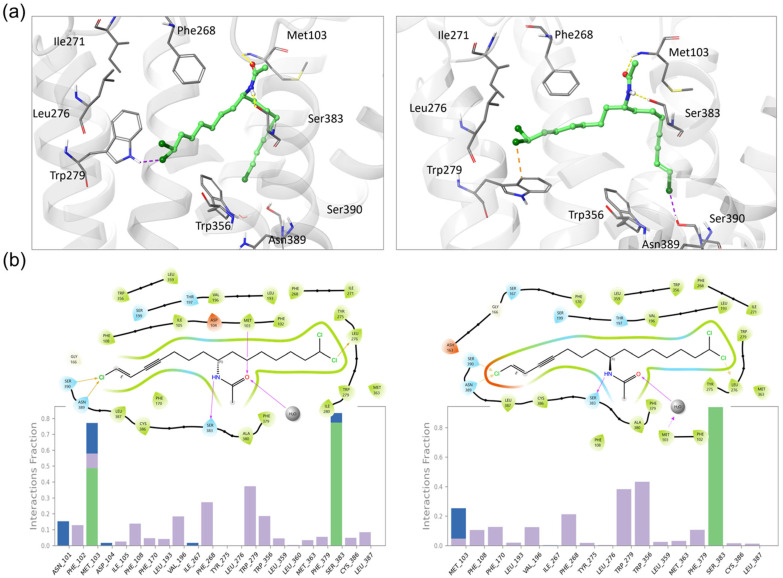
Molecular modeling of taveuniamide F enantiomers into CNR1. (**a**) Docking poses obtained for (*R*)-(left) and (*S*)-taveuniamide F (right) stereoisomers using Induced Fit Docking with GlideSP precision. Yellow lines are hydrogen bonds, orange are halogen bonds, and purple are salt bridges. (**b**) Protein–ligand interactions during 100 ns MD, for (*R*)-(left) and (*S*)-taveuniamide F (right). In the frequency plots, the bar height represents the fraction of the simulated time in which the specific interaction was present. Green bars are hydrogen bonds, purple are hydrophobic contacts, and blue are water-bridged interactions.

To gain insights into the binding mode of **4** to CNR1, we performed modeling studies using the crystal structure of CNR1 in complex with the inhibitor taranabant at 2.6 Å resolution (PDBID:5U09). The docked structure of taranabant was mostly superimposed with the crystal structure, except for the trifluoropyridine ring, which points towards helix 3 (Figure S27). A Root Mean Square Deviation (RMSD) to the crystal structure of 0.5 Å is obtained if considering only the other atoms in the structure. A similar binding pose was obtained when we employed the induced fit docking (IFD) protocol (Figure S27). Using the same protocol, we initially applied it to the natural product (*R*)-**4**, and then we tested the enantiomer of **4** to probe the importance of the configuration on binding. The docking scores of both enantiomers obtained were the same. In the lowest energy docked poses (docking scores: (*R*)-**4** = −9.4, (*S*)-**4** = −9.0), the acetamide nitrogen of **4** acts as a hydrogen bond (HB) donor to Ser383, and the acetamide oxygen accepts HB from Met103 NH (Figure 6a). These bonds anchor **4** in position, with the chloroenyne moiety reaching into the pocket occupied by taranabant’s benzonitrile, and the dichloromethyl pointing in the same direction as taranabant’s chlorophenyl, toward a hydrophobic pocket including Trp279 and Trp356 (Figure 6a). Upon comparing the CNR2 sequence in the Protein Data Bank (PDB), we observed that the loop containing Met102 is shortened, missing the Glu93-Met103 segment, and its position is followed by a proline, which may explain the observed selectivity to CNR1.

During molecular dynamics simulations (MD), both stereoisomers were stable in the pocket, keeping the same interactions as the docking poses, and are predicted to make similar contacts with the protein. Both forms are anchored in the binding site by the hydrogen bond from the acetamide-H to Ser383, which is present 77% of the time with (*R*)-**4** and 94% of the time with the (*S*)-**4**. The second HB (from Met103 to acetamide-O) is weakened for the (*S*) form, being present only when bridged by water and less than 30% of the time, while it is still present for almost 80% of the time in the simulation of the (*R*)-**4** (Figure 6b) This indicates that (*S*)-**4** would likely have a weaker affinity to CNR1 than the natural product, (*R*)-**4**, and highlights the importance of Met103, which is absent in CNR2.

## 3. Materials and Methods

### 3.1. General Experimental Procedure

The optical rotations were measured using a Perkin-Elmer 341 polarimeter. ^1^H and 2D NMR spectra for the natural products (**1**–**4**) were obtained in CDCl_3_ using Agilent VNMRS-600 MHz and a 5 mm cold probe spectrometer. The spectra were referenced using the residual solvent signal [*δ*_H/C_ 7.26/77.16]. The NMR data for MTPA amide derivatives of **4** were obtained in CDCl_3_ using Cryo 600 MHz Bruker Avance III (Billerica, MA, USA). The spectra were referenced using the residual solvent signal [*δ*_H_ 7.26]. The HRESIMS data were obtained in the positive mode using an Agilent LC-TOF mass spectrometer (Santa Clara, CA, USA) equipped with APCI/ESI multimode ion source-detector.

### 3.2. Biological Material

The abundant cyanobacterium VPG14-26 was collected from Piti Bomb Holes, Guam, on 10 June 2014. Morphologically, the cyanobacterium resembled *Symploca* sp. with somewhat stiff upright tufts that were reddish in color.

### 3.3. Extraction and Isolation

The freeze-dried sample was subjected to non-polar extraction with 1:1 EtOAc–MeOH and polar extraction with 1:1 EtOH–H_2_O. The non-polar extract was subsequently partitioned between EtOAc and H_2_O. The EtOAc fraction was fractionated using silica column chromatography applying a gradient of increasing polarity (DCM, 90% DCM/iPrOH, 80% DCM/iPrOH, 50% DCM/i-PrOH, 50% DCM/MeOH, MeOH). The fraction eluting with 90% DCM/iPrOH was further purified by C18 column (10% MeOH/H_2_O, 30% MeOH/H_2_O, 50% MeOH/H_2_O, 70% MeOH/H_2_O, 90% MeOH/H_2_O, MeOH, DCM). The fraction eluting at 90% MeOH/H_2_O was purified using reversed-phase [Luna C18, 250 × 10.0 mm; flow rate, 4.0 mL/min; PDA detection 200–800 nm] using a linear MeCN–H_2_O gradient (65–100% MeCN over 21 min, 100% ACN for 5 min) to afford twenty-three fractions of compound mixtures. Fractions 8 (*t*_R_ 15 min), 11 (*t*_R_ 16.5 min), and 14 (*t*_R_ 18 min) were subjected to purification. Fraction 8 was purified using [SynergiHydro 250 × 4.6 mm; flow rate, 4.0 mL/min; PDA detection 200–800 nm] using 70% MeCN/H20 to afford **4** (2.5 mg, *t*_R_ 13.8 min). Fraction 11 was purified using [SynergiHydro 250 × 4.6 mm; flow rate, 4.0 mL/min; PDA detection 200–800 nm] using 60% MeCN/H20, then re-purified using [SynergiHydro 250 × 4.6 mm; flow rate, 4.0 mL/min; PDA detection 200–800 nm] using 85% MeCN/H_2_O to afford **1** (0.6 mg, *t*_R_ 7.1 min). Fraction 14 was purified using [SynergiHydro 250 × 4.6 mm; flow rate, 4.0 mL/min; PDA detection 200–800 nm] using 70% MeCN/H20 to afford **2** (2.0 mg, *t*_R_ 18 min) and **3** (0.06 mg, *t*_R_ 19.8 min).

Taveuniamide L (**1**): White amorphous solid; [α]^23^_D_ −11 (*c* 0.034, CHCl_3_); NMR data, ^1^H NMR, COSY, HSQC, HMBC, in CDCl_3_, see Table 1 and Supporting Information; HRESIMS *m*/*z*: 364.0996 [M + H]^+^ (calcd for C_17_H_25_NO^35^Cl_3_, 364.1002)

Taveuniamide M (**2**): White amorphous solid; [α]^23^_D_ −6 (*c* 0.19, CHCl_3_); NMR data, ^1^H NMR, COSY, HSQC, HMBC, in CDCl_3_, see Table 2 and Supporting Information; HRESIMS *m*/*z*: 400.0757 [M + H]^+^ (calcd for C_17_H_26_NO^35^Cl_4_, 400.0769)

Taveuniamide N (**3**): White amorphous solid; [α]^23^_D_ −4 (*c* 0.12, CHCl_3_); NMR data, ^1^H NMR, COSY, HSQC, HMBC, in CDCl_3_, see Table 3 and Supporting Information; HRESIMS *m*/*z*: 398.0600 [M + H]^+^ (calcd for C_17_H_24_NO^35^Cl_4_, 398.0612)

Taveuniamide F (**4**): White amorphous solid; [α]^23^_D_ −4 (*c* 0.054, CHCl_3_); NMR data, ^1^H NMR, COSY, HSQC, HMBC, in CDCl_3_, see Supporting Information; HRESIMS *m*/*z*: 366.1138 [M + H]^+^ (calcd for C_17_H_27_NO^35^Cl_3_, 366.1158)

### 3.4. Determination of the Absolute Configuration of Taveuniamide F (***4***)

Reduction to the secondary amine: Dry THF (13.5 equiv., 0.033 mmol) was added to stirred Cp_2_ZrHCl (3 equiv., 2.62 µmol) under argon, then added to 0.3 mg of **4** in THF (32.16 equiv., 0.026 mmol) under argon, stirring for 3 min. The reaction was quenched with water and then extracted into EtOAc twice, followed by a brine wash of the combined organic layers. The resulting extract was dried over Mg_2_SO_4_ and the pressure was reduced to elute the crude product. This was subjected to TLC and flash chromatography purification to give **5**.

MTPA-amidation: **5** was separated into 2 reaction vials (0.1 mg each, 0.30 µmol) and dissolved in DCM (50 µL) and DIEA (19.5 equiv., 1 µL) at room temperature under argon. Both (*S*)-MTPA-Cl and (*R*)-MTPA-Cl (19 equiv., 1 µL) were added under argon, respectively, and stirred overnight. The mixture was concentrated under nitrogen and taken up in 1 M NH_4_Cl and extracted into DCM twice. The combined organic layers were dried under Mg_2_SO_4_ and reduced pressure, and purified using reversed-phase HPLC [Phenomenex 4 µm Synergi™ 4 µm Hydro-RP 80 Å (4.6 × 250 mm), 10:0 ACN/H_2_O, detection at 235 nm], yielding (*R*)-MTPA amide (**6b**) and (*S*)-MTPA amide (**6a**), respectively.

Reduced analogue (**5**): White amorphous solid; HRESIMS *m*/*z*: 352.1353 [M + H]^+^, (calcd for C_17_H_29_^35^Cl_3_N, 352.1365).

(*S*)-MTPA amide product (**6a**): White amorphous solid; NMR data, 1H (600 MHz, CDCl_3_) δ 7.56 (m, 2H, Ph), δ 7.38 (m, 3H, Ph), δ 6.30 (d, *J* = 3 Hz, 1H, C_1_), δ 5.84 (dt, *J* = 7 Hz, 2 Hz, 1H, C_2_), δ 5.74 (m, 1H, C_15_), δ 3.73 (m, 1H, C_8_), δ 3.50 (s, 3H, OMe), δ 3.39 (m, 1H, C_16a_), δ 3.19 (m, 1H, C_16b_), δ 2.43 (m, 2H, C_5_), δ 2.19 (m, 2H, C_14_), δ 1.92 (m, 1H, C_7a_), δ 1.84 (m, 1H, C_7b_), δ 1.60 (p, *J* = 7 Hz, 2H, C_6a_), δ 1.54 (m, 2H, C_13_), δ 1.462 (m, 1H, C_9a_), δ 1.34 (m, 2H, C_12_), δ 1.30 (m, 1H, C_9b_), δ 1.28 (m, 2H, C_11_), δ 1.25 (m, 2H, C_10_), δ 0.53 (t, *J* = 7 Hz, 3H, C_17_); COSY, 1D TOCSY, see Supporting Information; HRESIMS *m*/*z* 568.1750 [M + H]^+^, (calcd for C_27_H_36_^35^Cl_3_F_3_NO_2_, 568.1764)

(*R*)-MTPA amide product (**6b**): White amorphous solid; NMR data, 1H (600 MHz, CDCl_3_) δ 7.56 (m, 2H, Ph), δ 7.38 (m, 3H, Ph), δ 6.30 (d, *J* = 3 Hz, 1H, C_1_), δ 5.84 (dt, *J* = 7 Hz, 2 Hz, 1H, C_2_), δ 5.74 (m, 1H, C_15_), δ 3.73 (m, 1H, C_8_), δ 3.50 (s, 3H, OMe), δ 3.39 (m, 1H, C_16a_), δ 3.19 (m, 1H, C_16b_), δ 2.43 (m, 2H, C_5_), δ 2.19 (m, 2H, C_14_), δ 1.95 (m, 1H, C_7a_), δ 1.85 (m, 1H, C_7b_), δ 1.60 (p, 7 Hz, 2H, C_6a_), δ 1.54 (m, 2H, C_13_), δ 1.459 (m, 1H, C_9a_), δ 1.34 (m, 2H, C_12_), δ 1.29 (m, 1H, C_9b_), δ 1.28 (m, 2H, C_11_), δ 1.25 (m, 2H, C_10_), δ 0.53 (t, *J* = 7 Hz, 3H, C_17_); COSY, 1D TOCSY, see Supporting Information; HRESIMS *m*/*z*: 568.1750 [M + H]^+^, (calcd for C_27_H_36_^35^Cl_3_F_3_NO_2_, 568.1764)

### 3.5. Cannabinoid CNR1 Receptor Binding Assay 

The assay was carried out by Eurofins Panlabs Discovery Services (New Taipei City, Taiwan). Human recombinant cannabinoid CNR1 receptors expressed in rat hematopoietic Chem-1 cells were used in modified HEPES buffer (50 mM HEPES, pH 7.4, 5 mM MgCl_2_, 1 mM CaCl_2_, 0.2% BSA). A 5 µg aliquot of membrane homogenate was incubated with 2 nM [^3^H]SR141716A for 60 min at 37 °C. Non-specific binding was estimated in the presence of 10 μM CP 55,940. Membranes were filtered and washed four times and the filters were counted to determine [^3^H]SR141716A as specifically bound. Compound **4** was screened at 8 concentrations, where the highest concentration was 50 µM. All compounds were dissolved in DMSO to give a final concentration of 1% DMSO. The experiments were carried out as technical duplicates. 

### 3.6. cAMP Hunter™ eXpress CNR1 (CB1) CHO-K1 GPCR Assay

The experiments (agonist and antagonist mode) were carried out by DiscoveRx Corporation (Fremont, CA, USA). Human CNR1 in stably transfected CHO-K1 cells were seeded in a total volume of 20 μL into white-walled, 384-well microplates and incubated at 37 °C overnight. Before testing, cell plating media was exchanged with 10 μL of assay buffer (HBSS + 10 mM HEPES). 

As described in catalog ref. (86-0007P-2277AG) for the agonist mode, intermediate dilution of sample stocks was performed to generate 4× samples in assay buffer. A total of 5 μL of 4× sample and 5 μL of 4× forskolin were added to cells and incubated at 37 °C for 30 min. The final assay vehicle concentration was 1%. The results are expressed as a percent efficacy relative to the maximum response of the control ligand.

As described in catalog ref. (86-0007P-2277AN) for the antagonist mode, intermediate dilution of sample stocks was performed to generate 4× samples in assay buffer. A total of 5 μL of 4X sample was added to cells and incubated at 37 °C for 30 min, and 5uL of 4× EC80 of CP55940 in 4× forskolin reagent was added and cells incubated for 37 °C for 30 min. The final assay vehicle concentration was 1%. The results are expressed as a percent inhibition of the control ligand. The assays were performed at 10-point concentrations of **4** using 3-fold serial dilutions in duplicate, where the highest concentration was 50 µM.

### 3.7. PathHunter β-Arrestin GPCR Profiling

GpcrMAX panel biosensor assays were used to characterize taveuniamide F (**4**, 20 µM) (agonist and antagonist mode). The experiments were carried out by DiscoveRx Corporation (Fremont, CA, USA) as described before [37], using PathHunter β-arrestin enzyme fragment complementation (EFC) technology.

Dose–response curves were obtained by subjecting taveuniamide F (**4**) to a secondary antagonist screen utilizing ten GPCR biosensor assays (Arrestin) against the following targets: GPR119, NPSR1b, HTR2C, CCR9, CHRM4, GPR120, HTR2A, GPR103, CNR1, and CNR2. Stably transfected HEK293 cells expressing GPR119, U2OS cells expressing NPSR1b, HTR2C and HTR2A, and CHO-K1 cells expressing the rest of the mentioned receptors, were incubated with **4** for 30 min. The assays were performed at 10-point concentrations using 3-fold serial dilutions in duplicate, where the highest concentration was 50 µM.

### 3.8. MTT Cell Viability Assay

HEK293 cells were purchased from ATCC (Manassas, VA, USA). They were cultured and maintained in Dulbecco’s modified Eagle medium (DMEM; Invitrogen, Waltham, MA, USA) supplemented with 10% fetal bovine serum (FBS; HyClone, Logan, UT, USA) and 1% antibiotic-antimycotic (Invitrogen) at 37 °C in a humidified atmosphere with 5% CO_2_. HEK293 cells were seeded in 96-well plates at densities of 15,000 cells per well in 100 μL, respectively. After 16 h of incubation, the cells were treated with 0.5 μL of various concentrations of compounds or a DMSO solvent control. Following 24 h of incubation, cells were treated with 3-(4,5-dimethylthiazol-2-yl)-2,5-diphenyltetrazolium bromide according to the manufacturer’s instructions (Promega, Madison, WI, USA). The experiments were performed as technical triplicates.

### 3.9. Molecular Modeling and Simulations

Density Functional Calculations. The amide derivative of **4** was built using Maestro, with both *R* and *S* configurations for C8. Conformer searches were initiated from either the Z or E conformer of the amide bond with Schrödinger’s AutoConf.py program. In this scheme, up to 1 million conformers are generated for each starting structure. Duplicate conformers are filtered out, and the lowest 5 conformers with the lowest PM6 heats of formation are selected. Those are geometry-optimized at the B3LYP-D3/LACVP** level of theory, and finally ranked by single point energy calculations with M06-2X/cc-pVTZ(-f) at the previously optimized geometries. The coordinate scan of the amide bond angle was carried out by starting with the lowest energy conformer and rotating the bond from 0° to 360° with 20° increments. At each point, the geometry of the molecule was reoptimized at the B3LYP-D3/6-31G** level of theory, keeping the angle fixed at the corresponding value. Finally, single point energies were obtained for the final geometries at the M06-2X/cc-pVTZ(-f) level of theory. The effect of the solvent was estimated at the same level with C-PCM solvent model, and chloroform as solvent. All QM calculations were carried out with *Schroding*er’s Jaguar software (Jaguar version 12.1, release 125, included in Schrodinger Suite 2023-3).

CNR1 model. The model for CNR1 was obtained from the crystal structure of CNR1 in complex with the inhibitor taranabant at 2.6 Å resolution (PDBID:5U09) [38]. The structure was prepared using Schrödinger Protein Preparation Workflow at pH 7.4 ± 1.0. The ligands taranabant and taveuniamide F (**4**) were drawn using Maestro 2D sketcher and prepared with Schrödinger LigPrep. To verify the importance of the stereochemical configuration, we modeled both *R* and *S* forms of **4**.

Dockings. Taranabant was docked in the CNR1 binding site using Schrödinger GlideSP [39], and also with Schrödinger’s Induced Fit Docking protocol (IFD) [40]. Compound **4** was docked into the binding site only with the IFD protocol, and the bound structure with the lowest Glide Docking Score and Glide Emodel scores were selected for molecular dynamics simulations.

Molecular Dynamics. MD simulations were carried out with Schrödinger Desmond MD engine [41]. The systems were embedded in a POPC membrane, which was included in an orthorhombic box with SPC waters. Counterions were added to neutralize the charges, and extra ion pairs were added to reach a salt concentration of 0.15 M. The OPLS4 force field was used for all atoms except water. The simulations used the NP*γ*T ensemble at 1 atm pressure and 300 K. Membrane models were relaxed with Schrödinger standard membrane relaxation protocol. Simulations were run in two stages: one initial simulation of 10.2 ns for system relaxation, and a second 100 ns simulation for data acquisition.

## 4. Conclusions and Outlook

In summary, this study has uncovered new halogenated enyne fatty acid amides, taveuniamide L-N (**1**–**3**), and taveuniamide F (**4**), from a marine cyanobacterium, emphasizing the importance of cyanobacteria as a rich source of structurally unusual, bioactive natural products. Through HRESIMS and NMR analysis, the structures of taveuniamides L-N (**1**–**3**) were elucidated. The absolute configuration at C-8 of this structural class was determined as *R* via a comprehensive analysis of **4**, employing Mosher’s amide analysis and DFT studies. The biological characterization of taveuniamide F (**4**) highlights its ability to modulate GPCRs, particularly CNR1, with antagonist/inverse agonist effects.

The unique structure features of taveuniamides, including an odd-numbered fatty acid chain (C15), halogenation at the termini, and an enyne system consistently present at one or both termini, make them intriguing from a biosynthesis perspective. Exploring the enzymatic machinery of taveuniamides’ biosynthesis can provide valuable insights and resources for the field of biocatalysis. It can lead to the discovery of novel enzymes, facilitate the development of improved biocatalysts, and contribute to a deeper understanding of enzymatic mechanisms.

## Figures and Tables

**Table 1:** NMR Spectroscopic data (600 MHz, CDCl_3_) for taveuniamide L (**1**).

C/H No.	*δ*_C_, Type	*δ*_H_ (*J* in Hz)	COSY	HMBC
1	128.8, CH	6.43, d (13.6)	2	
2	114.0, CH	5.90, dt (13.6, 2.3)	1, 5	
3	92.7, C			
4	76.1, C			
5	19.1, CH_2_	2.32, td (7.6, 2.3)	1, 2, 6	1, 3, 4, 6, 7
6	24.8, CH_2_	1.55	5, 7a	7, 8
7	34.4, CH_2_	a: 1.41 b: 1.61	8, 7b, 6 8, 7a	5, 8, 9 5, 8, 9
8	48.7, CH	3.93, dtd (12.7, 8.8, 4.5)	7a, 7b, 9a, 9b, NH	9, 10
9	35.3, CH_2_	a: 1.33 b: 1.49	8, 9b 8, 9a	
10	25.5, CH_2_	1.33		
11	28.8, CH_2_	1.32		
12	28.0, CH_2_	1.40	13	10
13	29.4, CH_2_	2.15, dt (7.3, 7.4)	12, 14	12, 14, 15
14	129.8, CH	5.83, t (7.3)	13	
15	119.8, C			
16	169.5,C			
17	23.5, CH_3_	1.98, s		16
NH		5.07, d (9.3)	8	

**Table 2:** NMR spectroscopic data (600 MHz, CDCl_3_) for taveuniamide M (**2**).

C/H No.	*δ*_C_, Type	*δ*_H_ (*J* in Hz)	COSY	HMBC
1	128.6, CH	6.43, d (13.6)	2, 5	2, 4
2	114.0, CH	5.90, dt (13.6, 2.3)	1, 5	1, 3
3	92.7, C			
4	76.2, C			
5	19.1, CH_2_	2.32, td (7.4, 2.0)	1, 2, 6	1, 3, 4, 6, 7
6	24.8, CH_2_	1.55	5, 7a	5, 7, 8
7	34.4, CH_2_	a: 1.41 b: 1.61	8, 7b, 6 8, 7a	5, 8, 9 5, 8, 9
8	48.7, CH	3.94, dtd (12.7, 8.8, 4.5)	7a, 7b, 9a, 9b, NH	9, 10
9	35.3, CH_2_	a: 1.34 b: 1.49	8, 9b 8, 9a	8, 10, 11
10	25.5, CH_2_	1.34		
11	28.9, CH_2_	1.33		
12	28.4, CH_2_	1.38	13	11, 10
13	26.2, CH_2_	1.76, dtd (11, 7.8, 5.8)	12, 14	12, 14, 15
14	55.0, CH_2_	2.66, m	13	12, 13, 15
15	100.3, C			
16	169.6, C			
17	23.5, CH_3_	1.98, s		16
NH		5.08, d (9.3)	8	8, 16

**Table 3:** NMR spectroscopic data (600 MHz, CDCl_3_) for taveuniamide N (**3**).

C/H No.	*δ*_C_, Type	*δ*_H_ (*J* in Hz)	COSY	HMBC
1	130.1, C			
2	111.1, CH	5.93, t (2.3)	5	
3	98.8, C			
4	74.9, C			
5	19.6, CH_2_	2.40, td (6.8, 2.3)	2, 6	1, 3, 4, 6, 7
6	24.8, CH_2_	1.59	5, 7a, 7b	
7	34.4, CH_2_	a: 1.44 b: 1.66	8, 7b, 6 8, 7a, 6	
8	48.8, CH	3.94, ddq (13.3, 8.9, 4.9)	7a, 7b, 9a, 9b, NH	
9	35.3, CH_2_	a: 1.34 b: 1.48	8, 9b 8, 9a	
10	25.8, CH_2_	1.33		
11	29.2, CH_2_	1.32		
12	28.2, CH_2_	1.40	13	10, 11
13	29.6, CH_2_	2.15, q (7.3)	12, 14	12, 14, 15
14	129.8, CH	5.84, t (7.4)	13	
15	119.5, C			
16	169.5, C			
17	23.7, CH_3_	1.98, s		16
NH		5.08, d (9.3)	8	

**Table 4:** IC_50_ of taveuniamide F (**4**) against GPCR targets identified in the PathHunter β-arrestin primary screen.

GPCR Target	IC_50_ (µM)
CNR1	1.84
CHRM4	1.92
NPSR1b	4.36
GPR120	4.72
HTR2A	4.83
CCR9	7.38
GPR119	11.67
GPR103	14.07
HTR2C	>50

## Data Availability

The data are contained within the article or Supplementary Materials. Raw data will be made available upon request.

## Supplementary Materials

The following supporting information can be downloaded at: https://www.mdpi.com/article/10.3390/md22010028/s1, Figure S1: Structurally related analogs to taveuniamides in the literature [15,16]; Figures S2–S18: ^1^H NMR, COSY, HSQC, HMBC data for taveuniamide L (**1**), taveuniamide M (**2**), taveuniamide N (**3**) and taveuniamide F (**4**); Figures S19–S23: ^1^H NMR, COSY, 1D TOCSY of MTPA amide derivatives of **4**; Figure S24: DFT calculations for the preferred configuration at the amide bond of the *R*-MTPA derivative of taveuniamide F (**4**). Figure S25: Results of taveuniamide F (**4**) in GpcrMAX Panel Primary screen (β-arrestin) at 20 µM in agonist and antagonist mode; Figure S26: Cell viability of taveuniamide F assay using HEK293 cells; Figure S27: Docking poses obtained for taranabant.
