# Supplementary material for: Chlorinated Enyne Fatty Acid Amides from a Marine Cyanobacterium: Discovery of Taveuniamides L-M and Pharmacological Characterization of Taveuniamide F as a GPCR Antagonist with CNR1 Selectivity
Source: Mar Drugs. 2023 Dec 30;22(1):28. doi: 10.3390/md22010028 (PMC10817531; doi:10.3390/md22010028)
Supplement: Supplementary file 1 [file marinedrugs-22-00028-s001.zip › marinedrugs-2498692-supplementary.pdf]

**Supplementary Materials**

# Chlorinated Enyne Fatty Acid Amides from a Marine Cyanobacterium: Discovery of Taveuniamides L-M and Pharmacological Characterization of Taveuniamide F as a GPCR Antagonist with CNR1 Selectivity

**Lobna A. Elsadek [1,2], Emma K. Ellis [1,2], Gustavo Seabra [1,2], Valerie J. Paul [3] and Hendrik Luesch [1,2]***

1 Department of Medicinal Chemistry, University of Florida, 1345 Center Drive, Gainesville, Florida 32610, United States
2 Center for Natural Products, Drug Discovery and Development (CNPD3), University of Florida, 1345 Center Drive, Gainesville, Florida 32610, United States
3 Smithsonian Marine Station at Ft. Pierce, 701 Seaway Drive, Ft. Pierce, Florida 34949, United States
* Correspondence: luesch@cop.ufl.edu

Figure S1: Structurally related analogs in the literature

Figure S2: $^1$H NMR (600 MHz, $CDCl_3$) of taveuniamide L (**1**)

Figure S3: HSQC (600 MHz, $CDCl_3$) of taveuniamide L (**1**)

Figure S4: COSY (600 MHz, $CDCl_3$) of taveuniamide L (**1**)

Figure S5: HMBC (600 MHz, $CDCl_3$) of taveuniamide L (**1**). The number of increments = 320

Figure S6: HMBC (600 MHz, $CDCl_3$) of taveuniamide L (**1**). The number of increments = 512

Figure S7: $^1$H NMR (600 MHz, $CDCl_3$) of taveuniamide M (**2**)

Figure S8: HSQC (600 MHz, $CDCl_3$) of taveuniamide M (**2**)

Figure S9: COSY (600 MHz, $CDCl_3$) of taveuniamide M (**2**)

Figure S10: HMBC (600 MHz, $CDCl_3$) of taveuniamide M (**2**)

Figure S11: $^1$H NMR (600 MHz, $CDCl_3$) of taveuniamide N (**3**)

Figure S12: HSQC (600 MHz, $CDCl_3$) of taveuniamide N (**3**)

Figure S13: COSY (600 MHz, $CDCl_3$) of taveuniamide N (**3**)

Figure S14: HMBC (600 MHz, $CDCl_3$) of taveuniamide N (**3**)

Figure S15: $^1$H NMR (600 MHz, $CDCl_3$) of taveuniamide F (**4**)

Figure S16: HSQC (600 MHz, $CDCl_3$) of taveuniamide F (**4**)

Figure S17: COSY (600 MHz, $CDCl_3$) of taveuniamide F (**4**)

Figure S18: HMBC (600 MHz, $CDCl_3$) of taveuniamide F (**4**)

Figure S19. $^1$H NMR (600 MHz, $CDCl_3$) of (*S*)-MTPA amide (**6a**) and (*R*)-MTPA amide (**6b**)

Figure S20. COSY (600 MHz, $CDCl_3$) of the (*S*)-MTPA amide (**6a**)

Figure S21. COSY (600 MHz, $CDCl_3$) of the (*R*)- MTPAamide (**6b**)

Figure S22. 1D TOCSY irradiating H-5 of the (*S*)-MTPA amide (**6a**) at 80 ms, 60 ms, 45 ms, 30 ms, and 15 ms

Figure S23. 1D TOCSY irradiating H-5 of the (*R*)-MTPA amide (**6b**) at 80 ms, 60 ms, 45 ms, 30 ms, and 15 ms

Figure S24. Results of DFT coordinate scan for the preferred configuration at the amide bond of the (*R*)-MTPA derivative of taveuniamide F

Figure S25: Results of taveuniamide F (**4**) in GPCR MAX Panel Primary screen (β-arrestin) at 20 µM in agonist and antagonist mode

Figure S26: Cell viability assay of taveuniamide F using HEK293 cells

Figure S27**:** Docking poses obtained for taranabant

Supplementary References

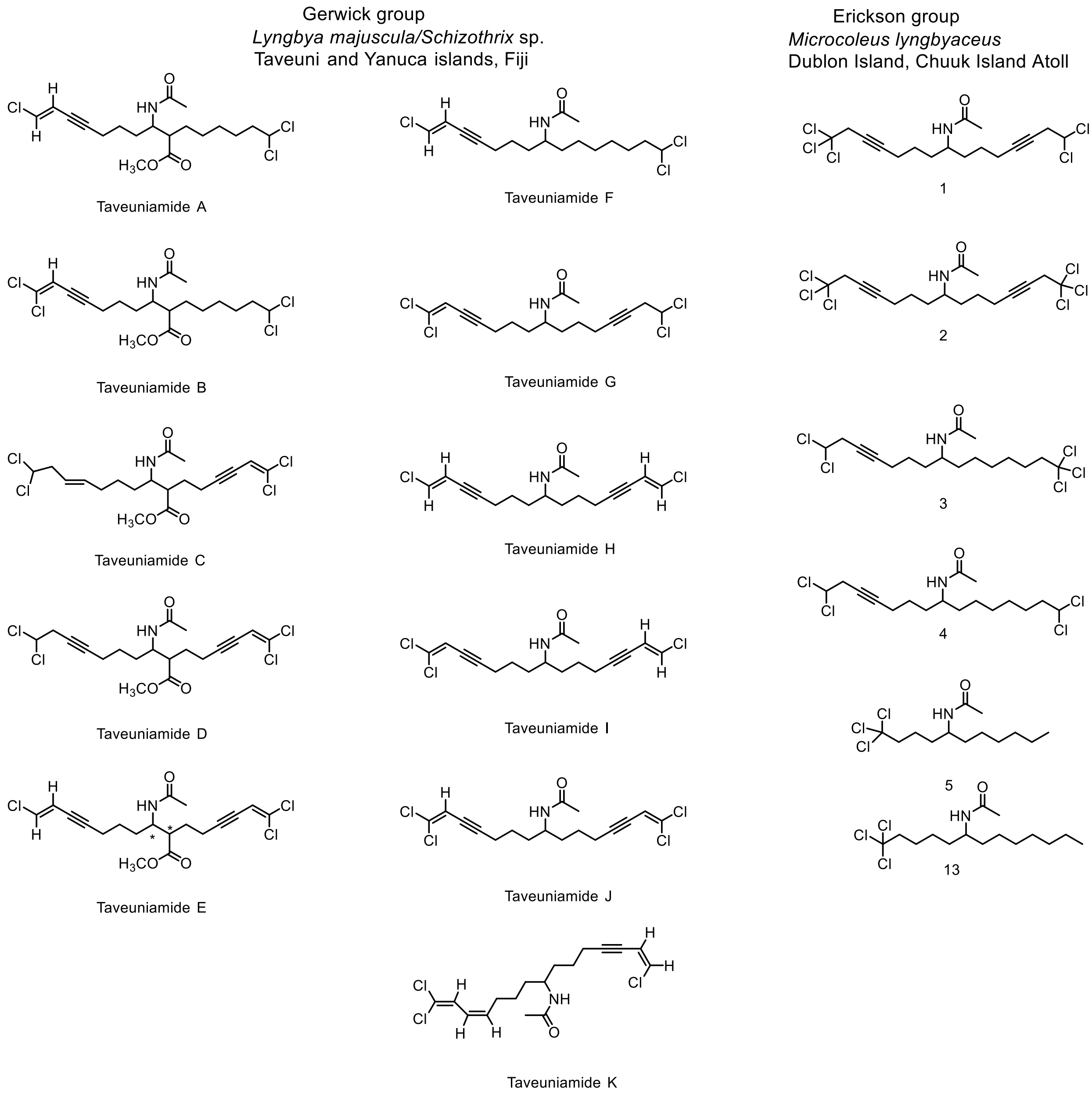


**Figure S1.** Structurally related analogs of taveuniamides in the literature.[1,2] The relative configuration of taveuniamide E was determined by the Gerwick group.[1]

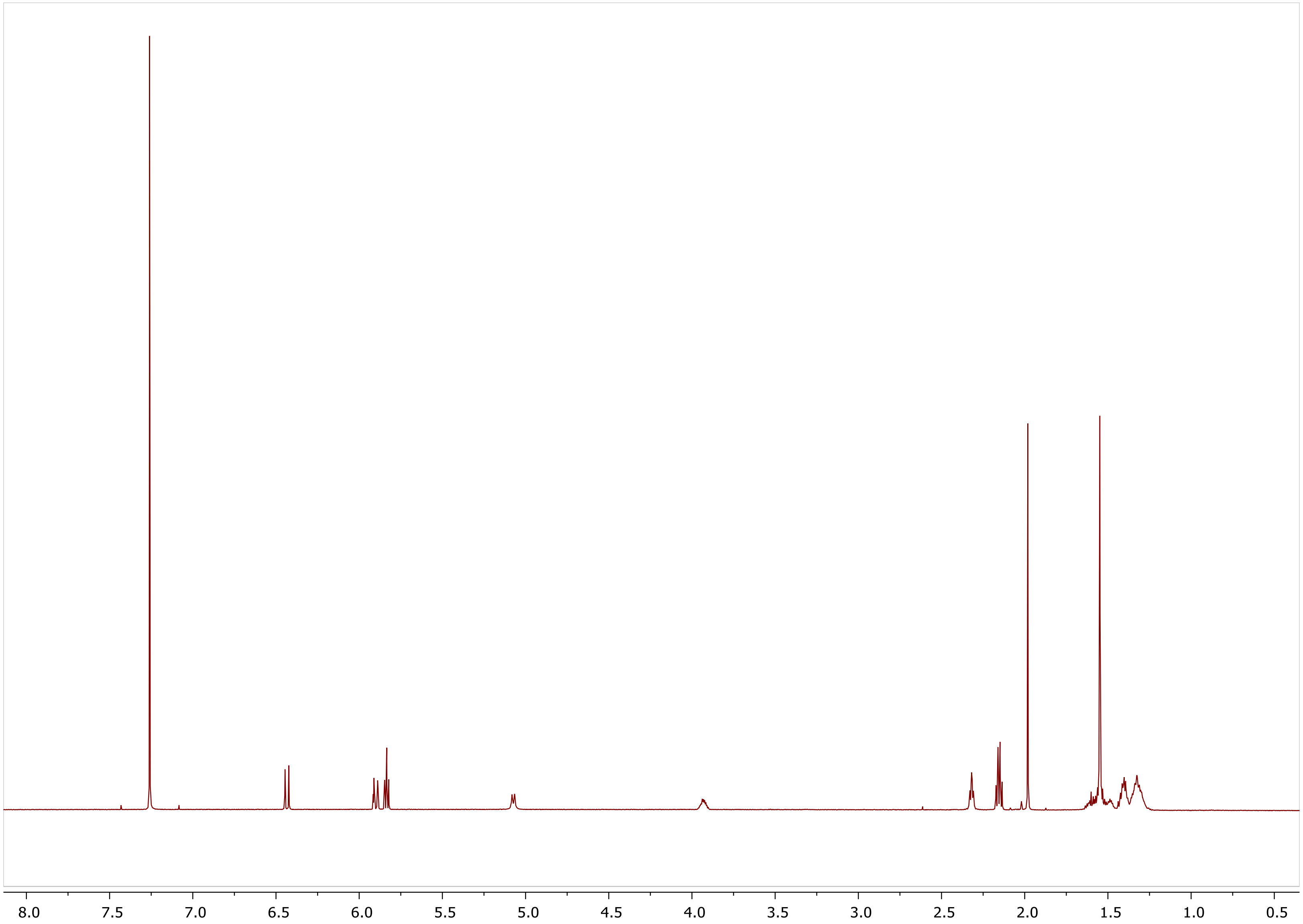


**Figure S2**: $^{1}$H NMR (600 MHz, $CDCl_3$) of taveuniamide L (**1**).

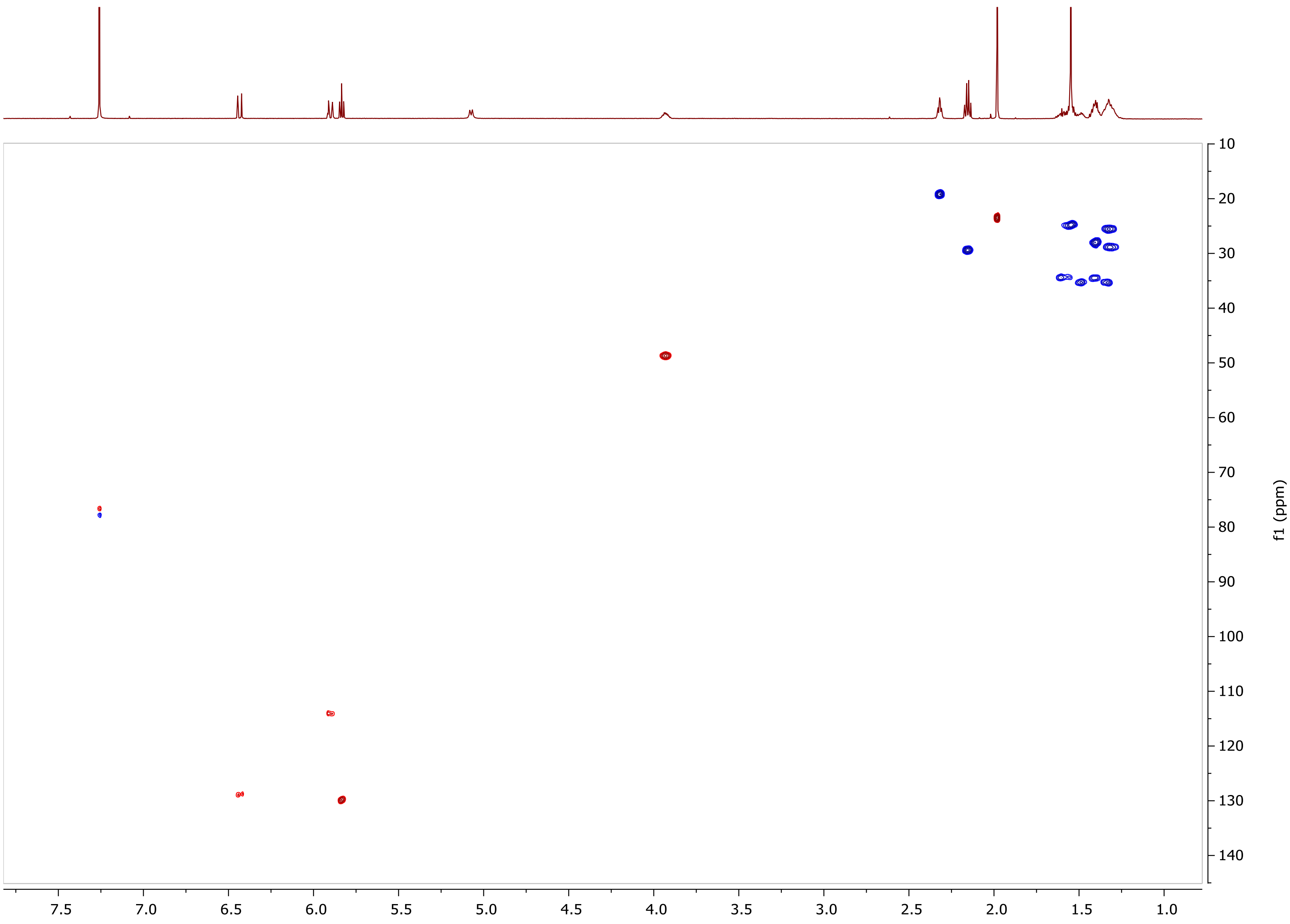


**Figure S3:** HSQC (600 MHz, $CDCl_3$) of taveuniamide L (**1**).

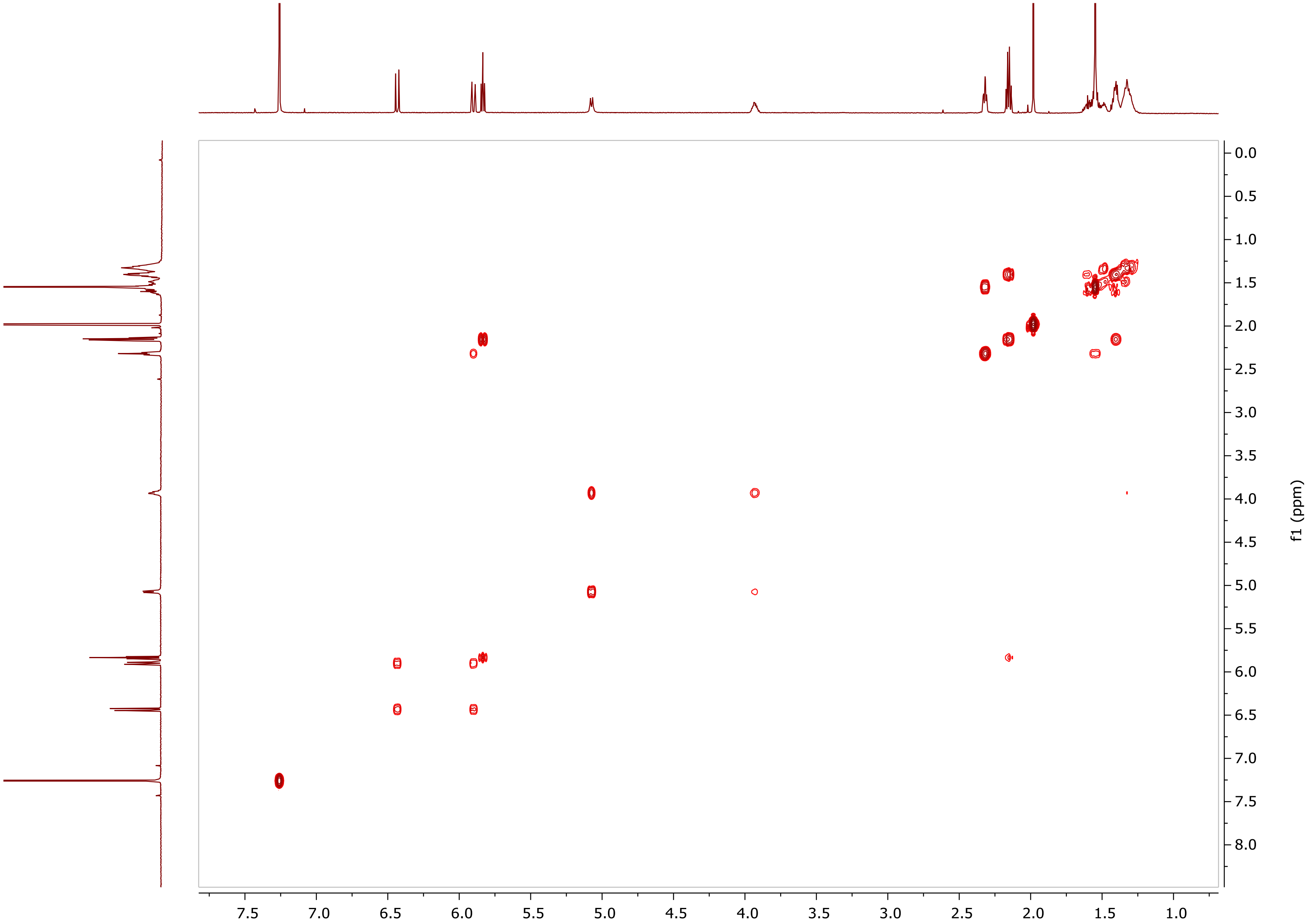


**Figure S4:** COSY (600 MHz, $CDCl_3$) of taveuniamide L (**1**).

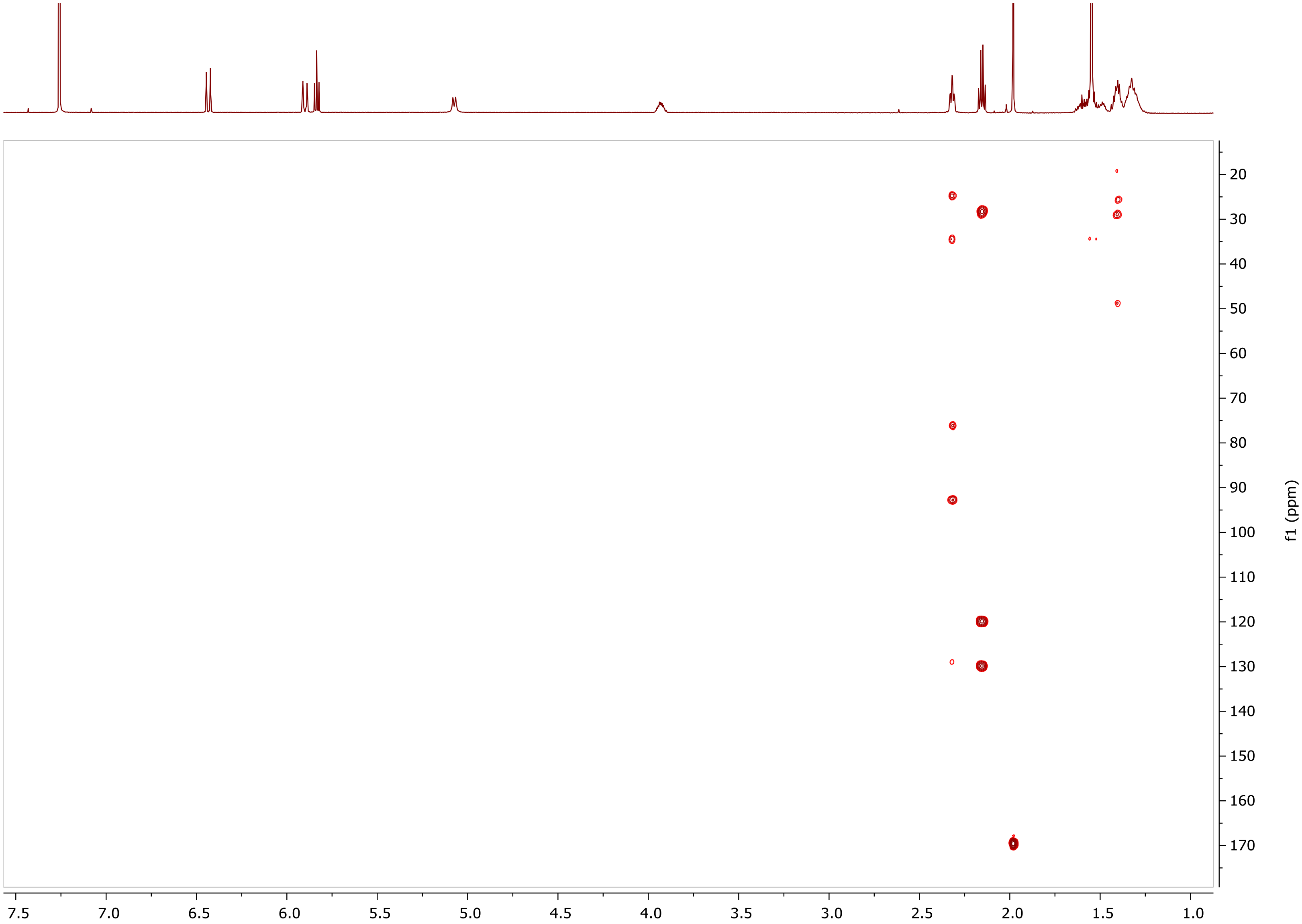


**Figure S5**: HMBC (600 MHz, $CDCl_3$) of taveuniamide L (**1**). The number of increments = 320

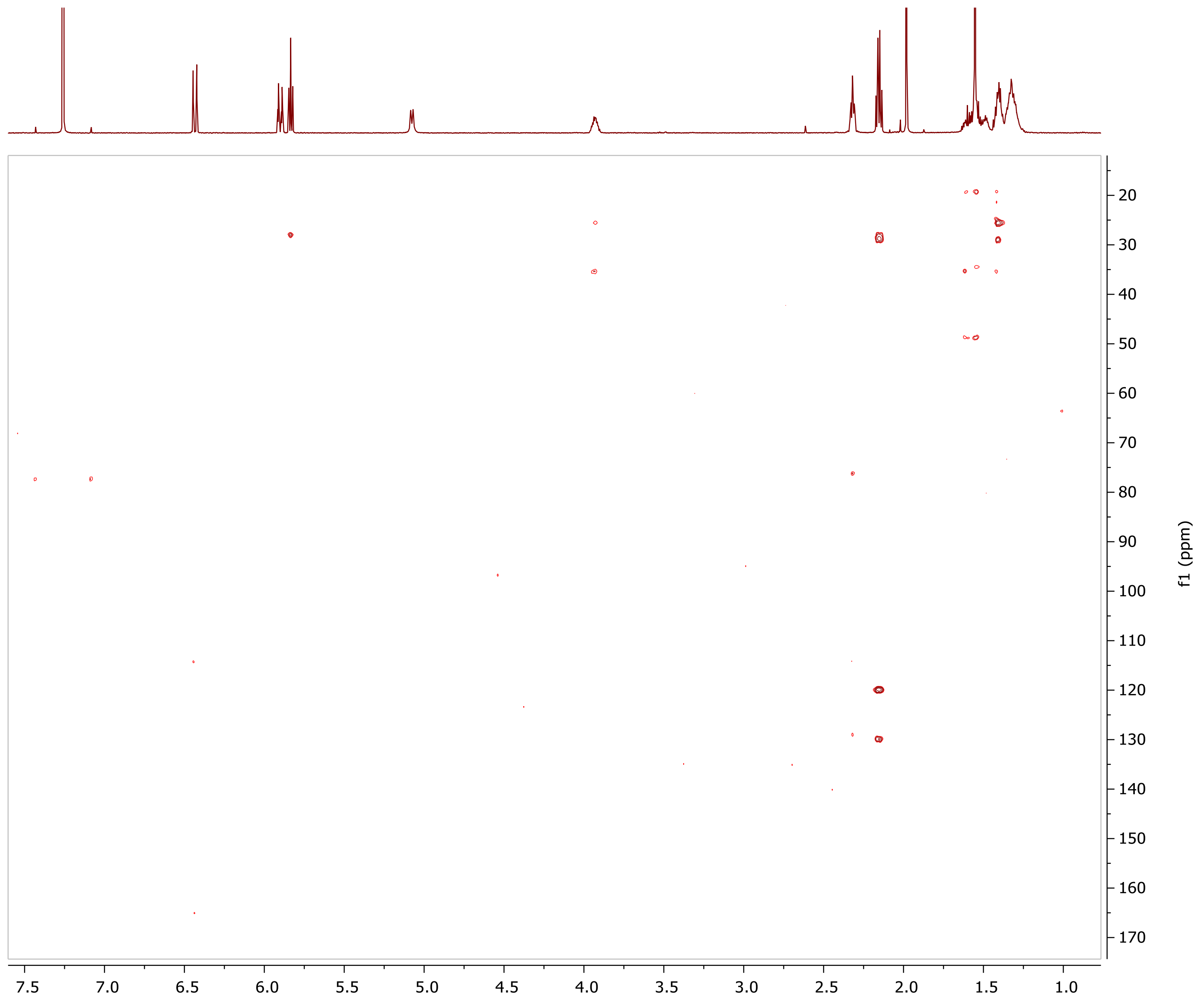


**Figure S6:** HMBC (600 MHz, $CDCl_3$) of taveuniamide L (**1**). The number of increments = 512.

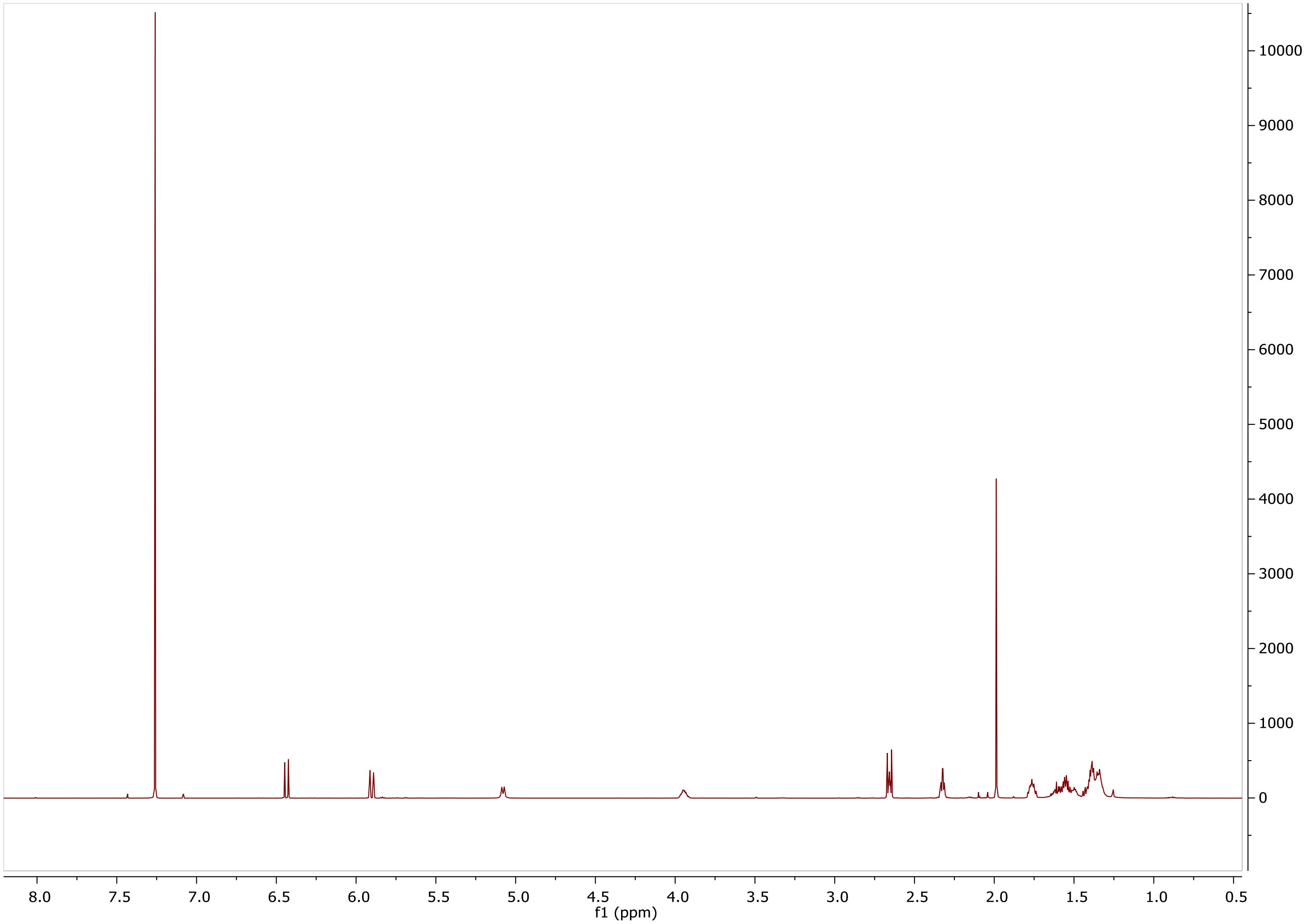


**Figure S7:** $^{1}H$ NMR (600 MHz, $CDCl_3$) of taveuniamide M (**2**).

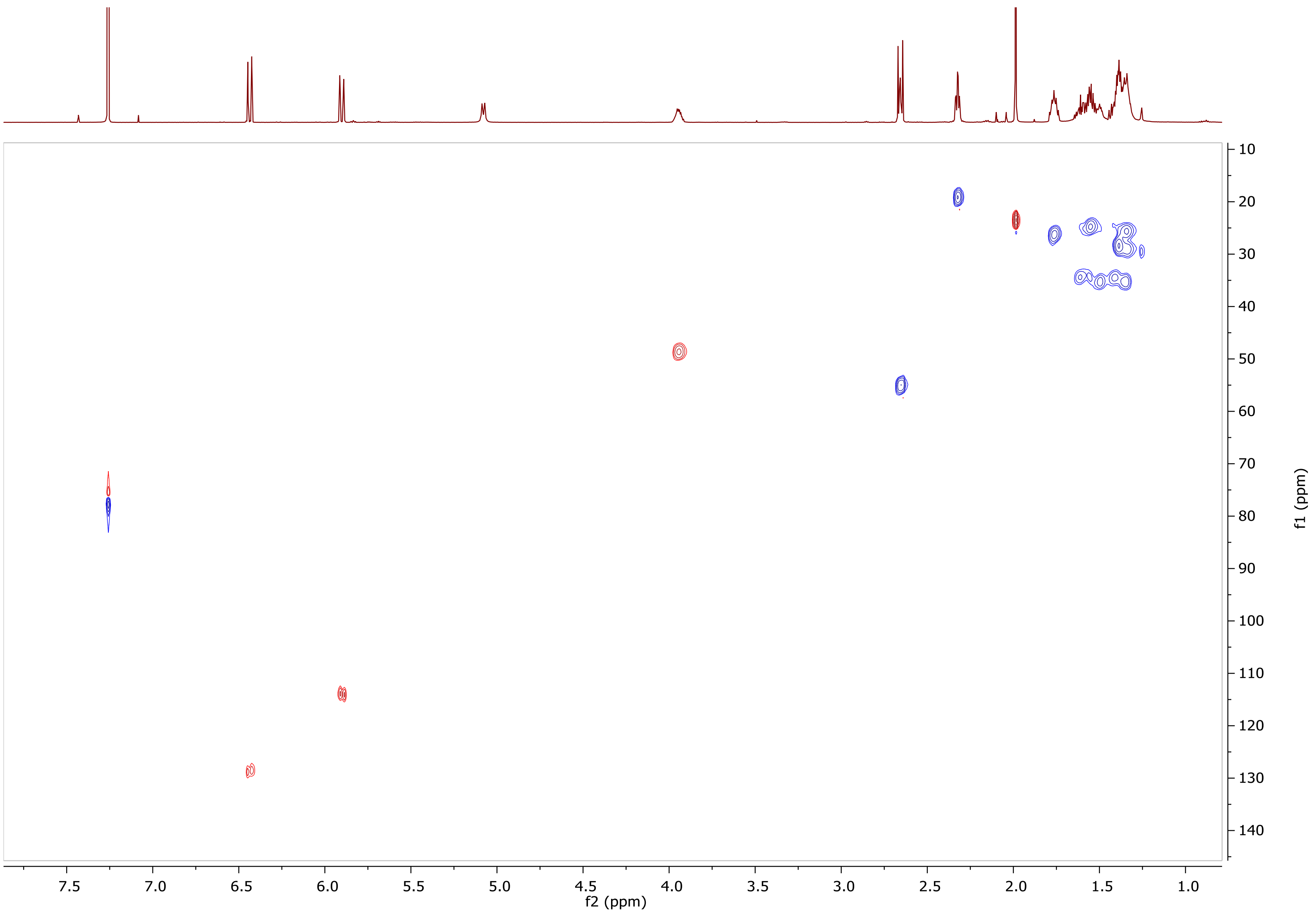


**Figure S8:** HSQC (600 MHz, $CDCl_3$) of taveuniamide M (**2**).

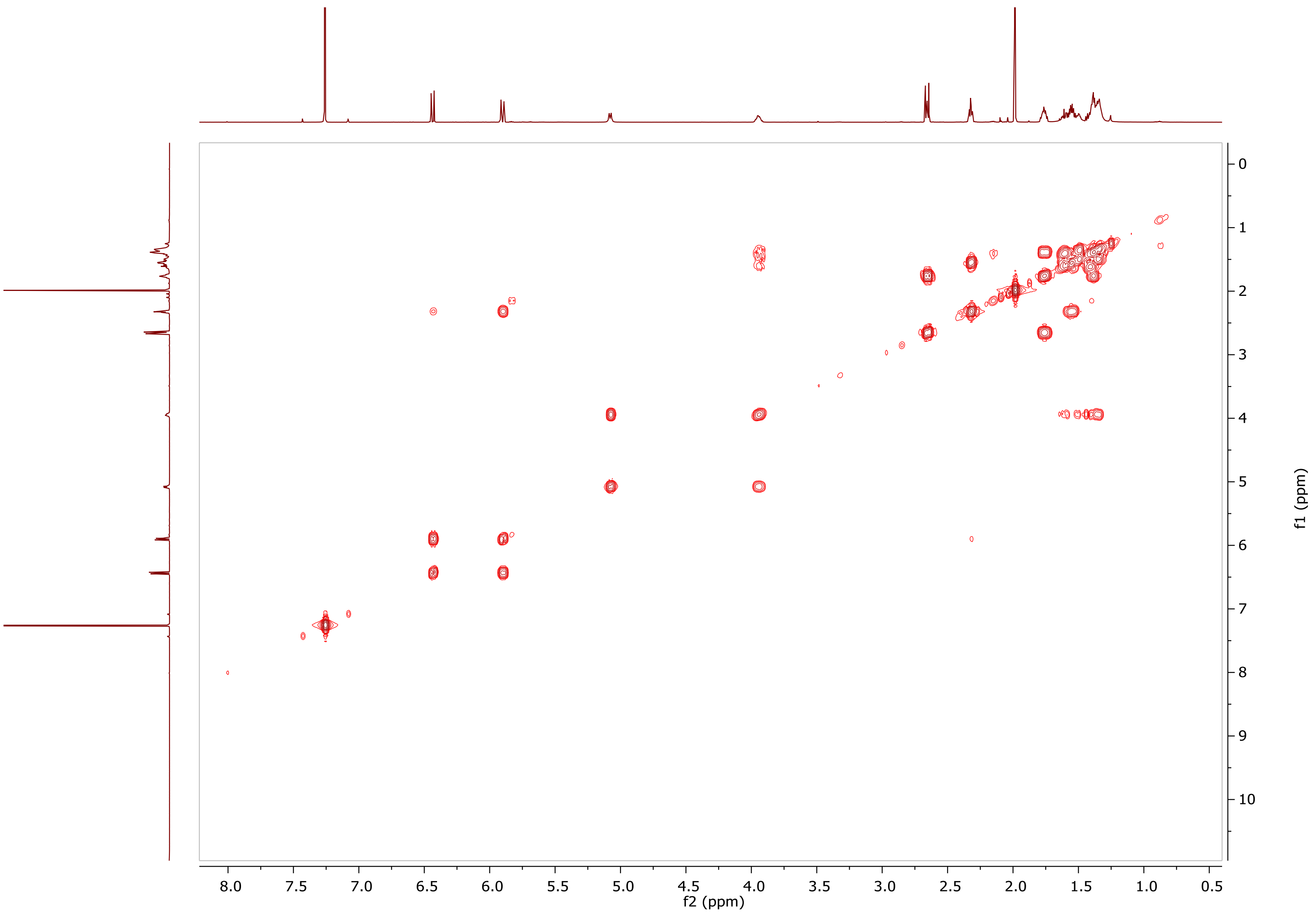


**Figure S9:** COSY (600 MHz, $CDCl_3$) of taveuniamide M (**2**).

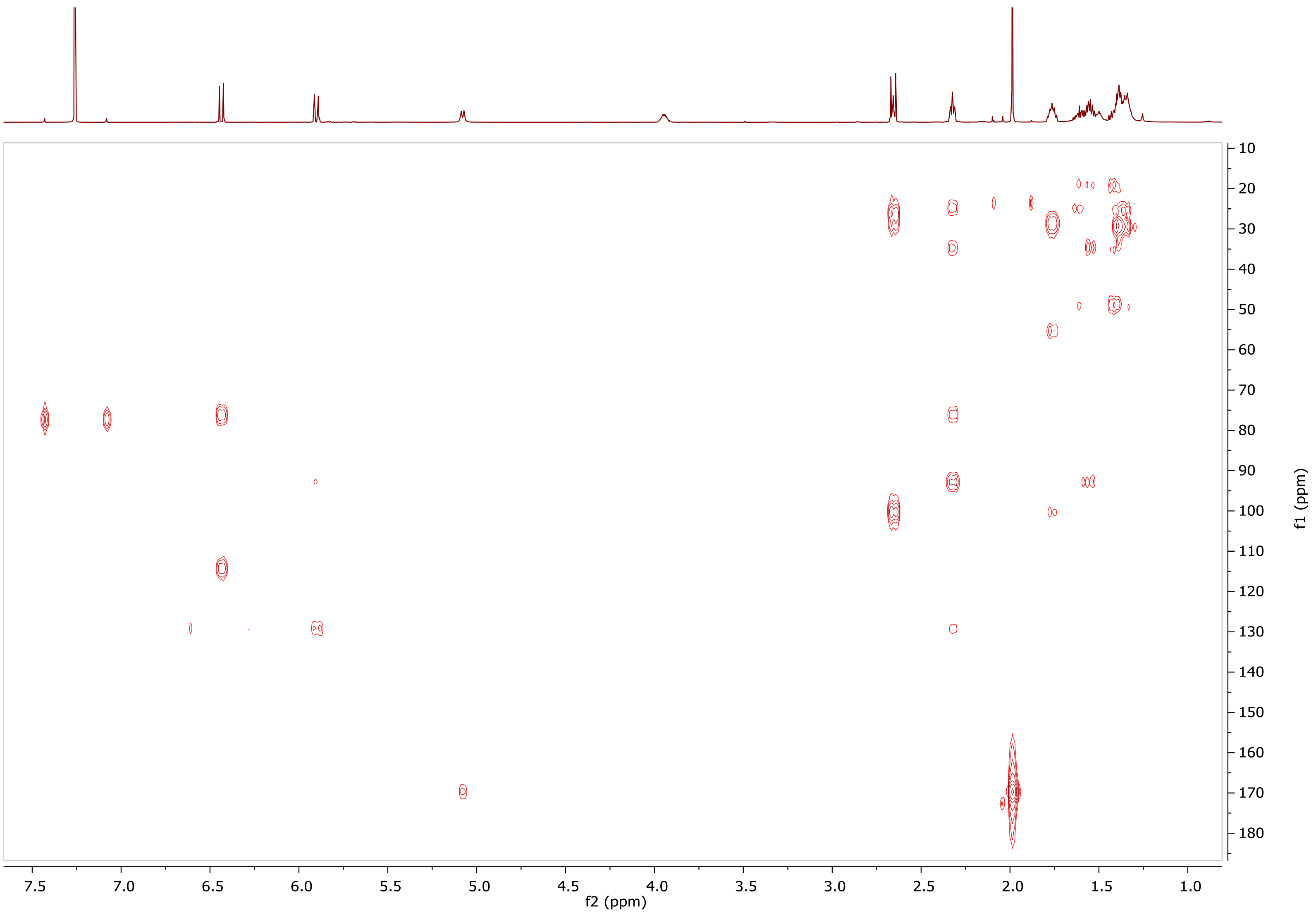


**Figure S10:** HMBC (600 MHz, $CDCl_3$) of taveuniamide M (**2**).

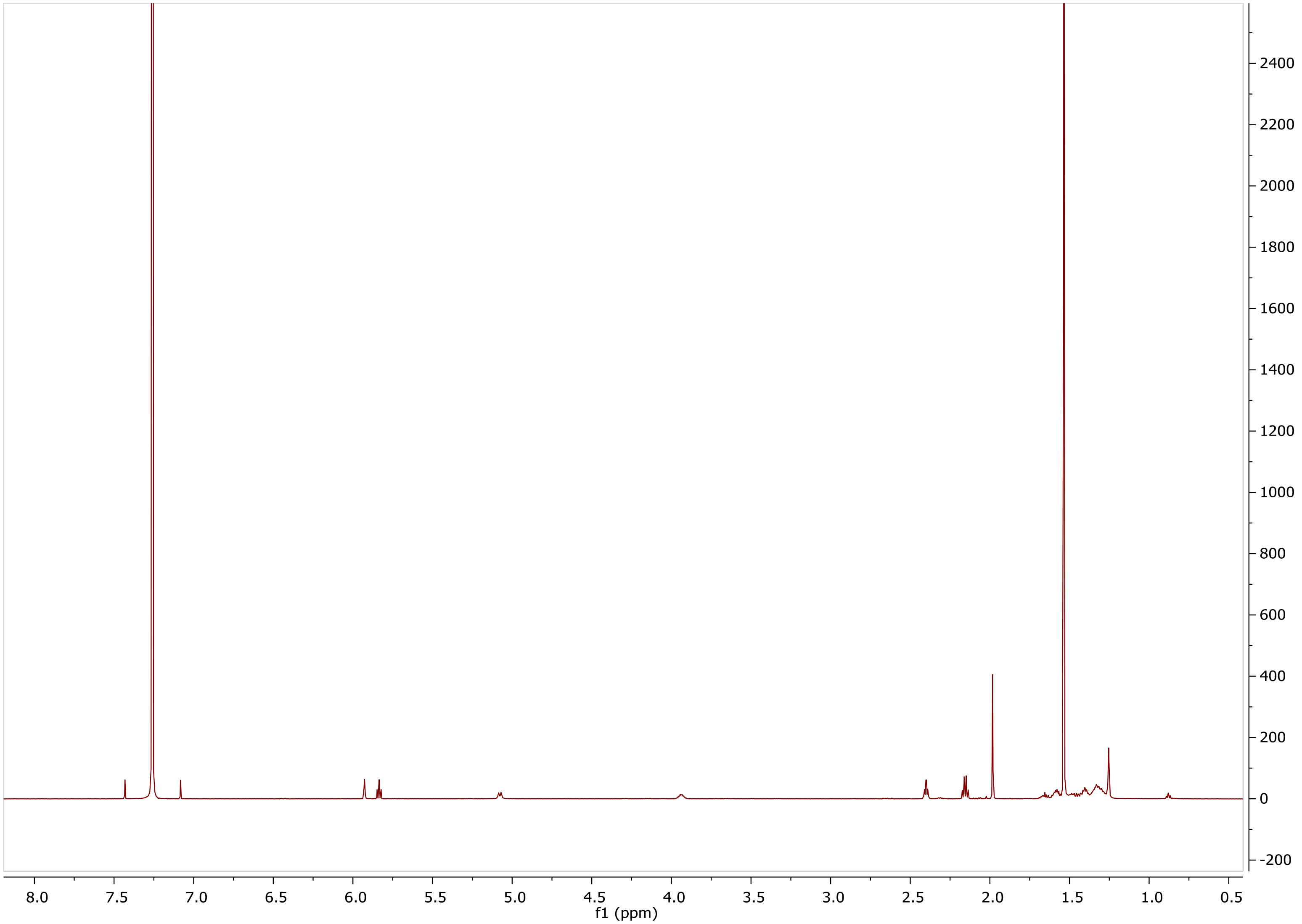


**Figure S11:** $^{1}$H NMR (600 MHz, $CDCl_3$) of taveuniamide N (**3**).

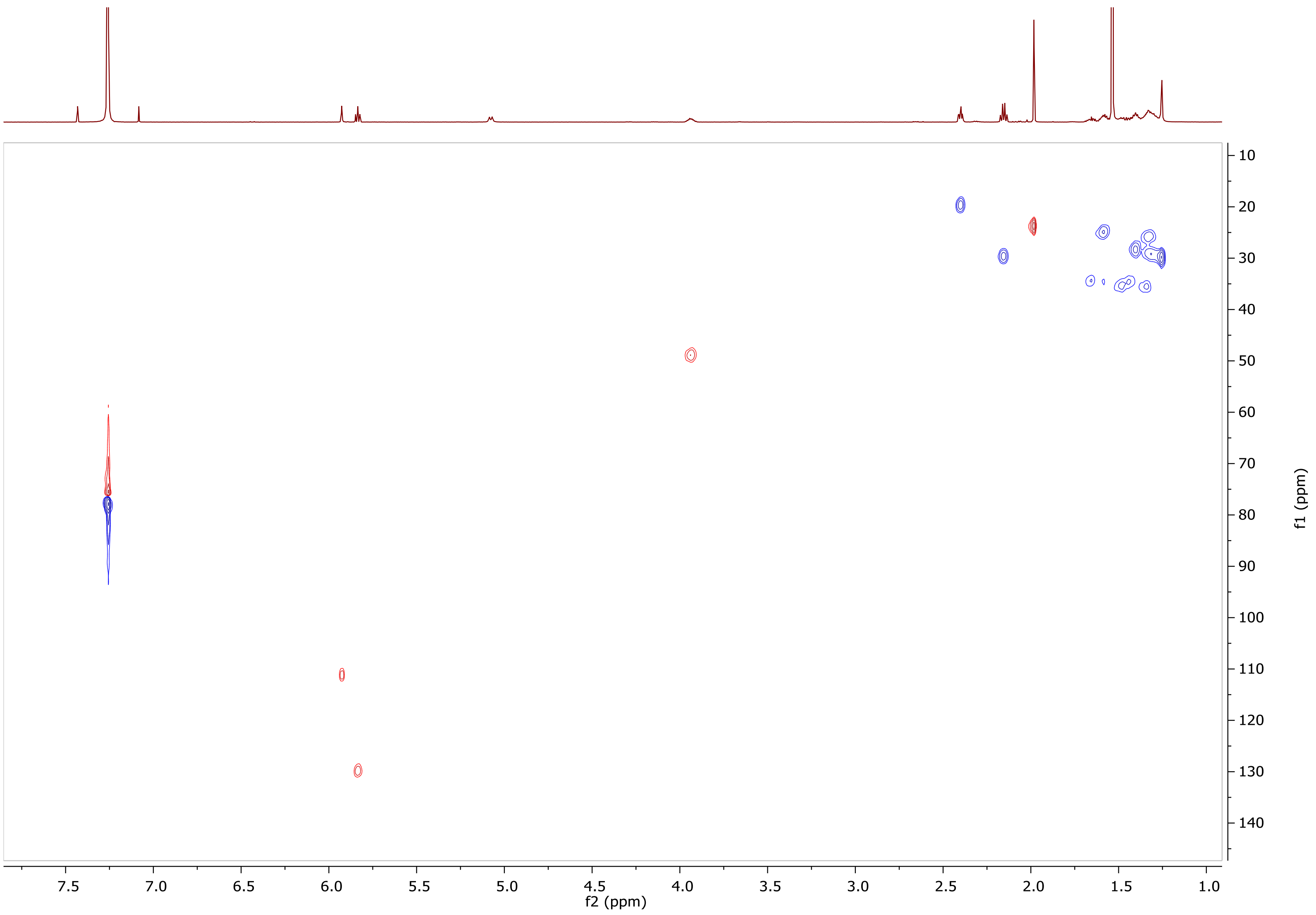


**Figure S12:** HSQC (600 MHz, $CDCl_3$) of taveuniamide N (**3**).

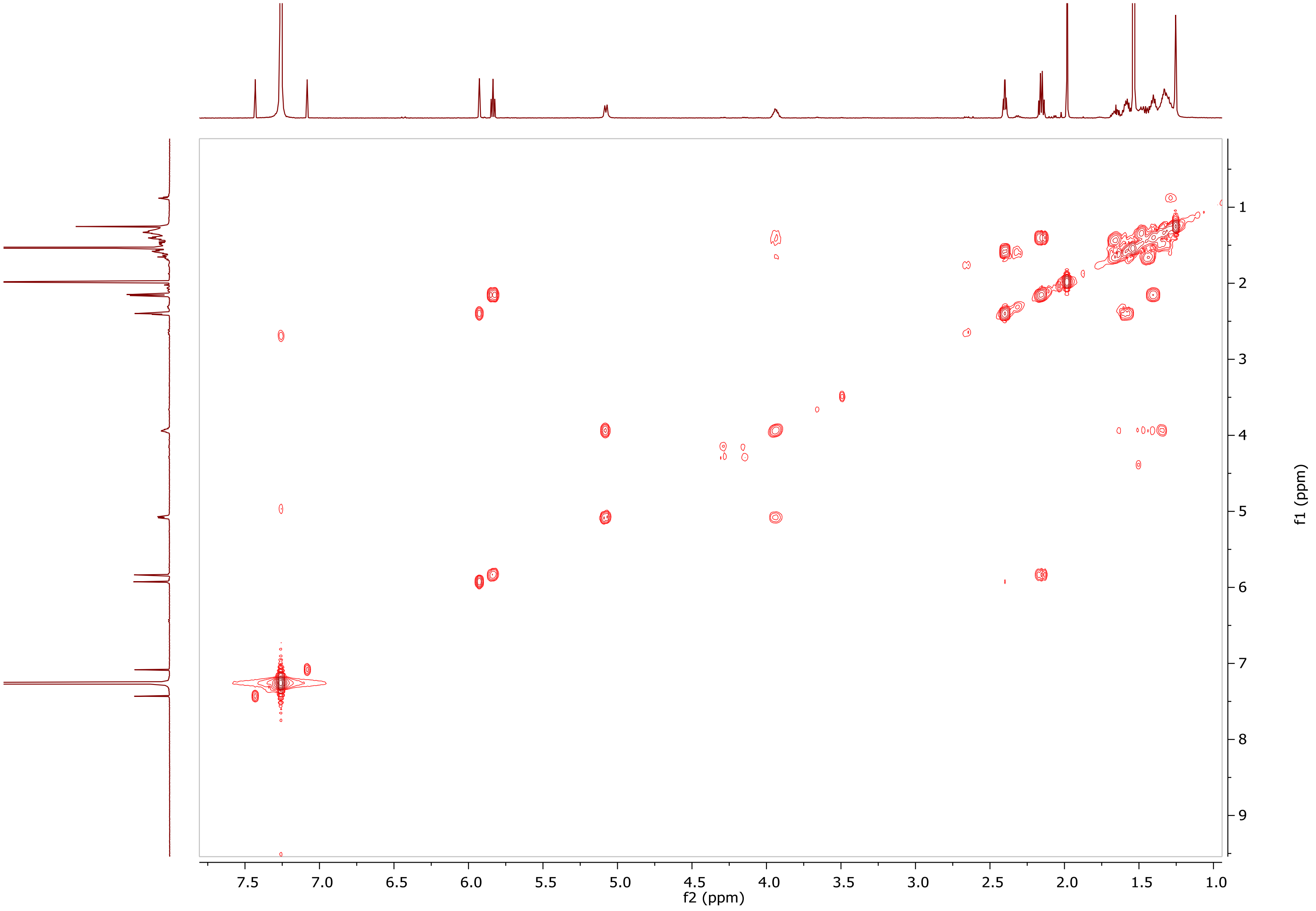


**Figure S13:** COSY (600 MHz, $CDCl_3$) of taveuniamide N (**3**).

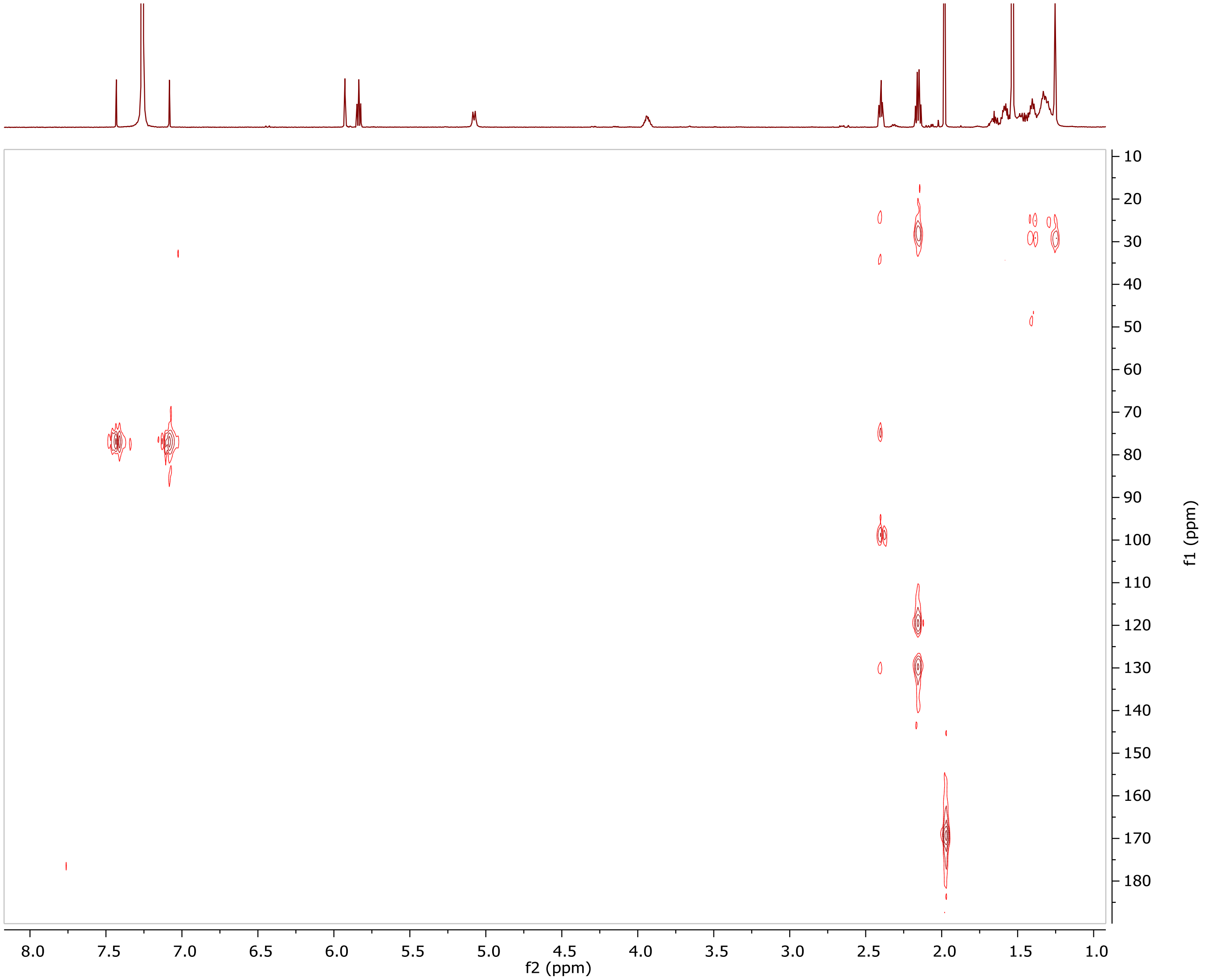


**Figure S14**: HMBC (600 MHz, $CDCl_3$) of taveuniamide N (**3**).

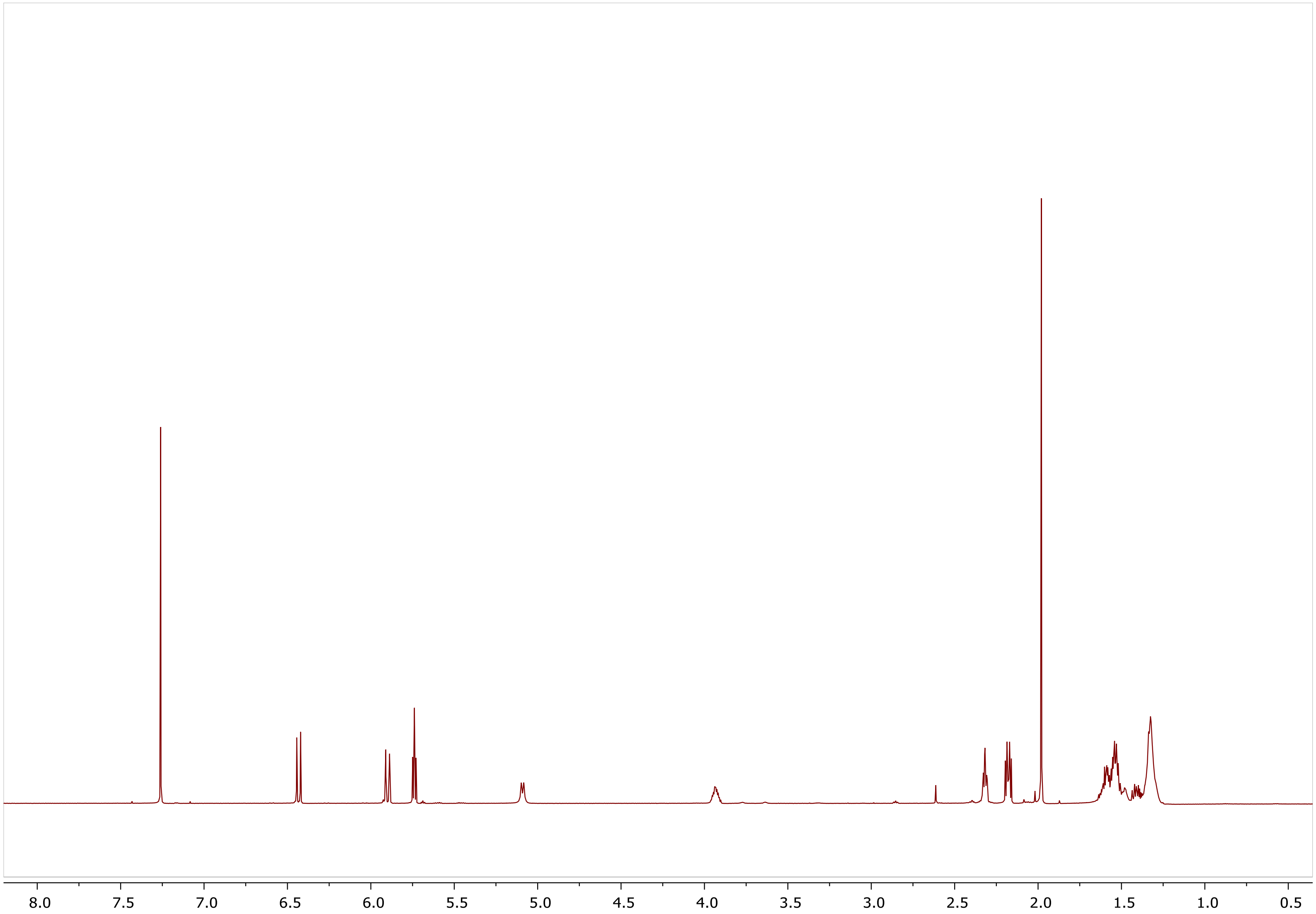


**Figure S15**: $^{1}H$ NMR (600 MHz, $CDCl_3$) of taveuniamide F (**4**).

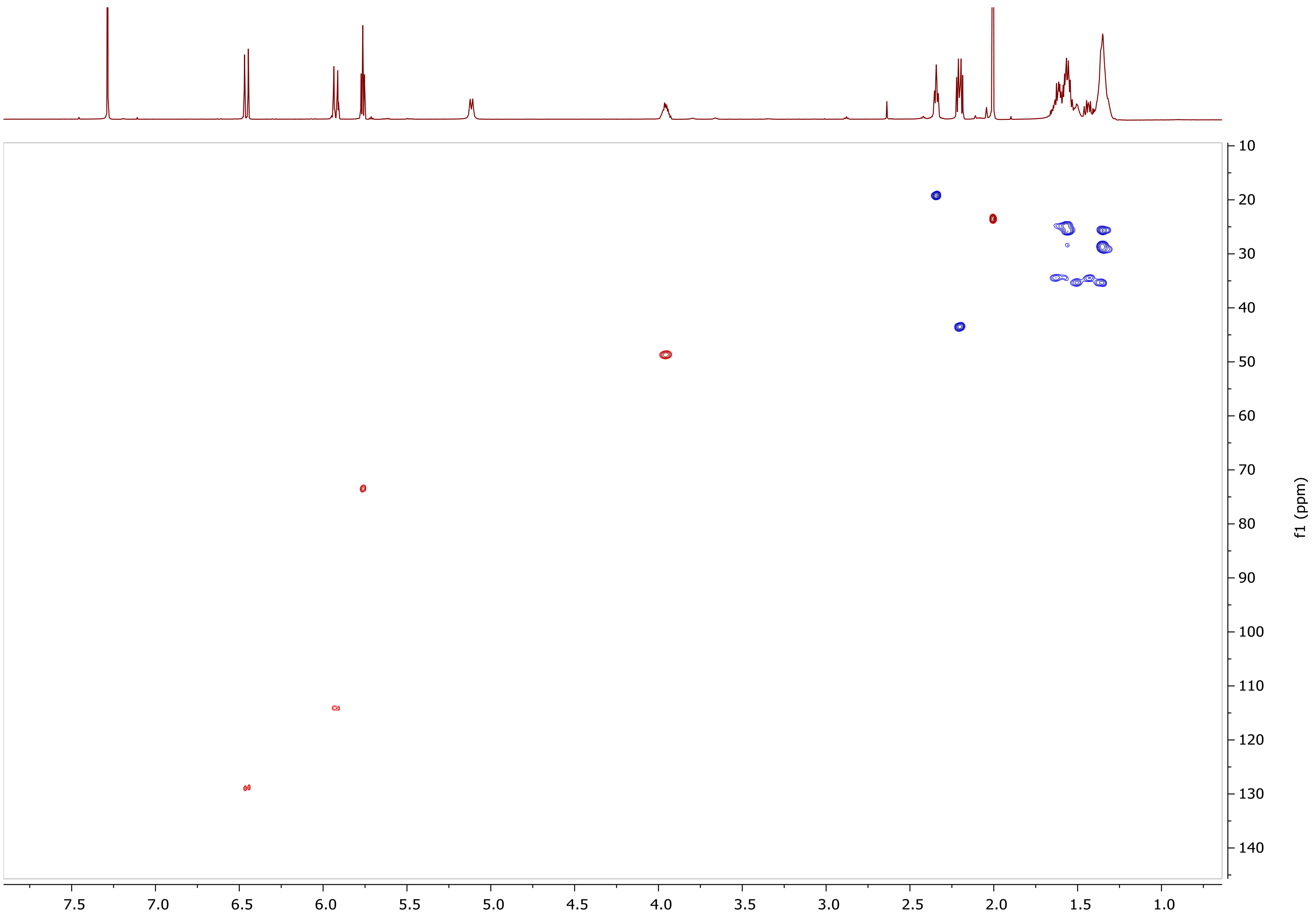


**Figure S16**: HSQC (600 MHz, $CDCl_3$) of taveuniamide F (**4**).

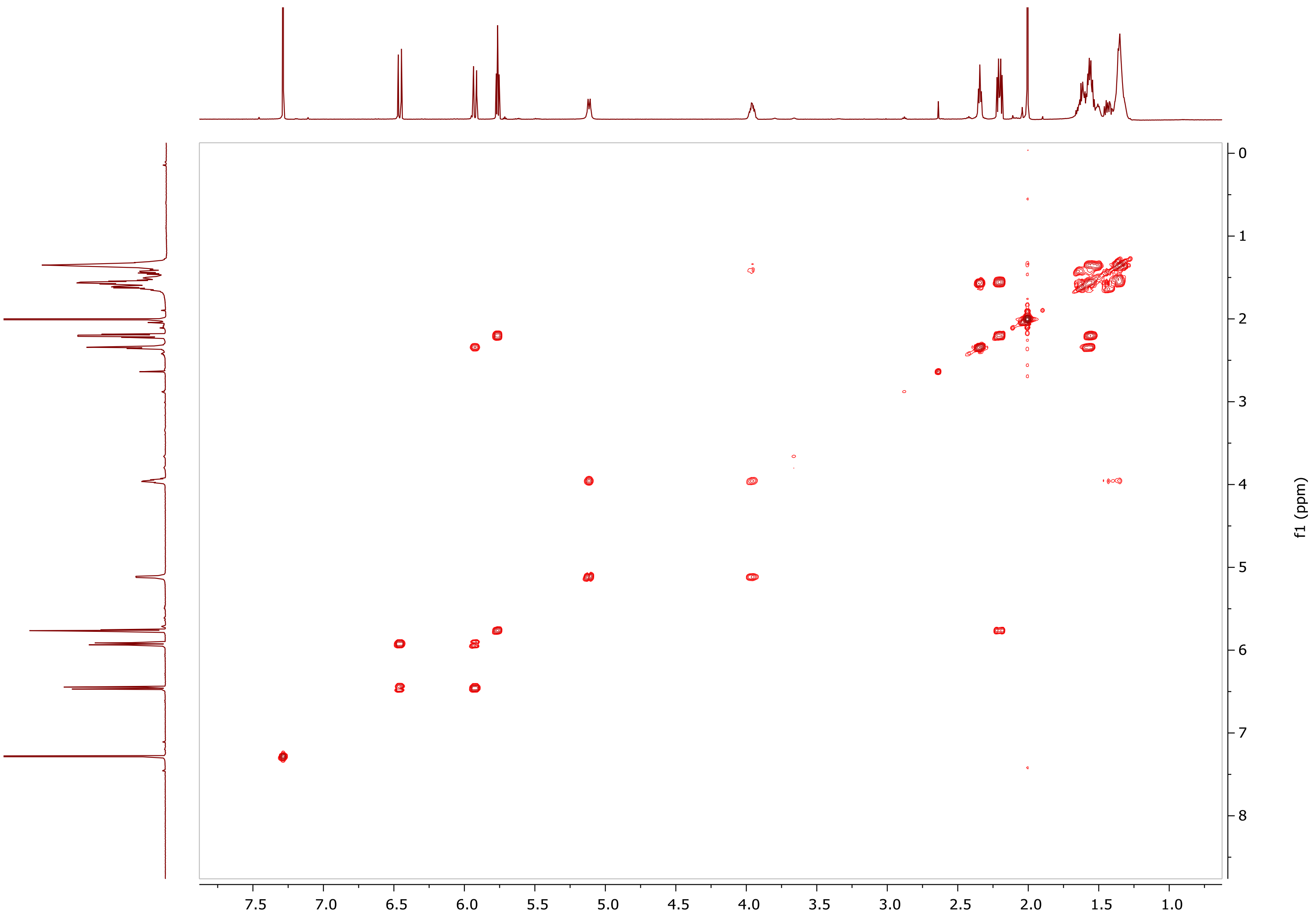


**Figure S17**: COSY (600 MHz, $CDCl_3$) of taveuniamide F (**4**).

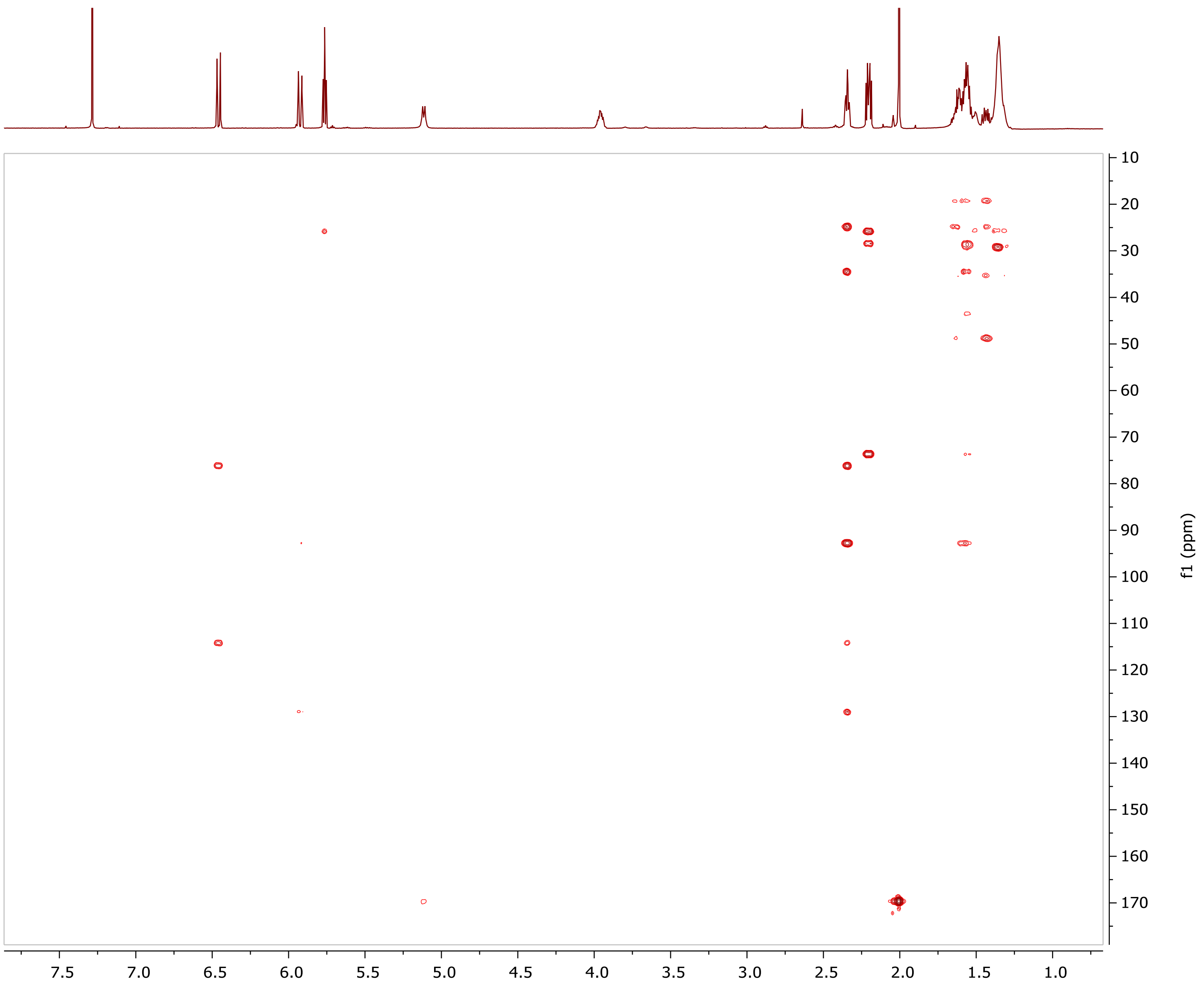


**Figure S18**: HMBC (600 MHz, $CDCl_3$) of taveuniamide F (**4**).

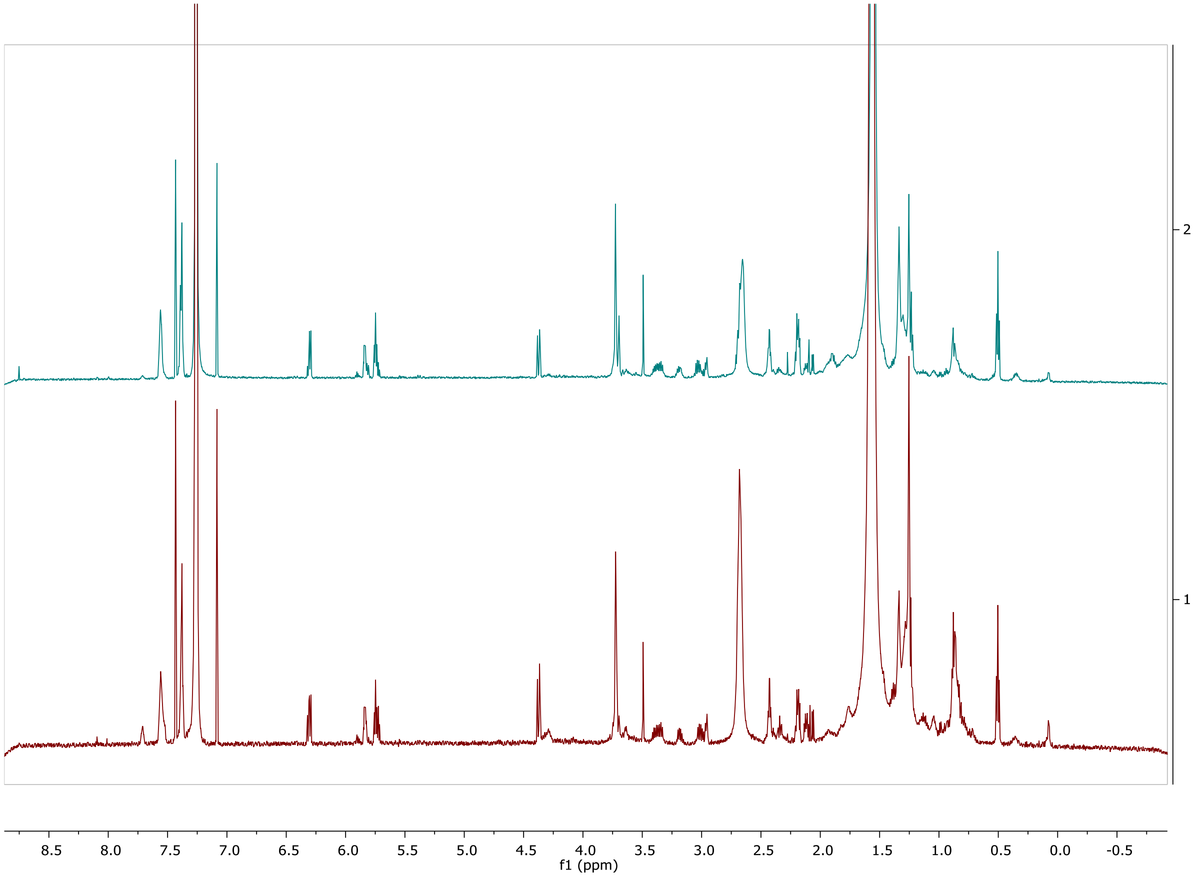


**Figure S19.** $^{1}$HNMR (600 MHz, $CDCl_3$) of (*S*)-MTPA amide (top, **6a**) and (*R*)-MTPA amide (bottom, **6b**).

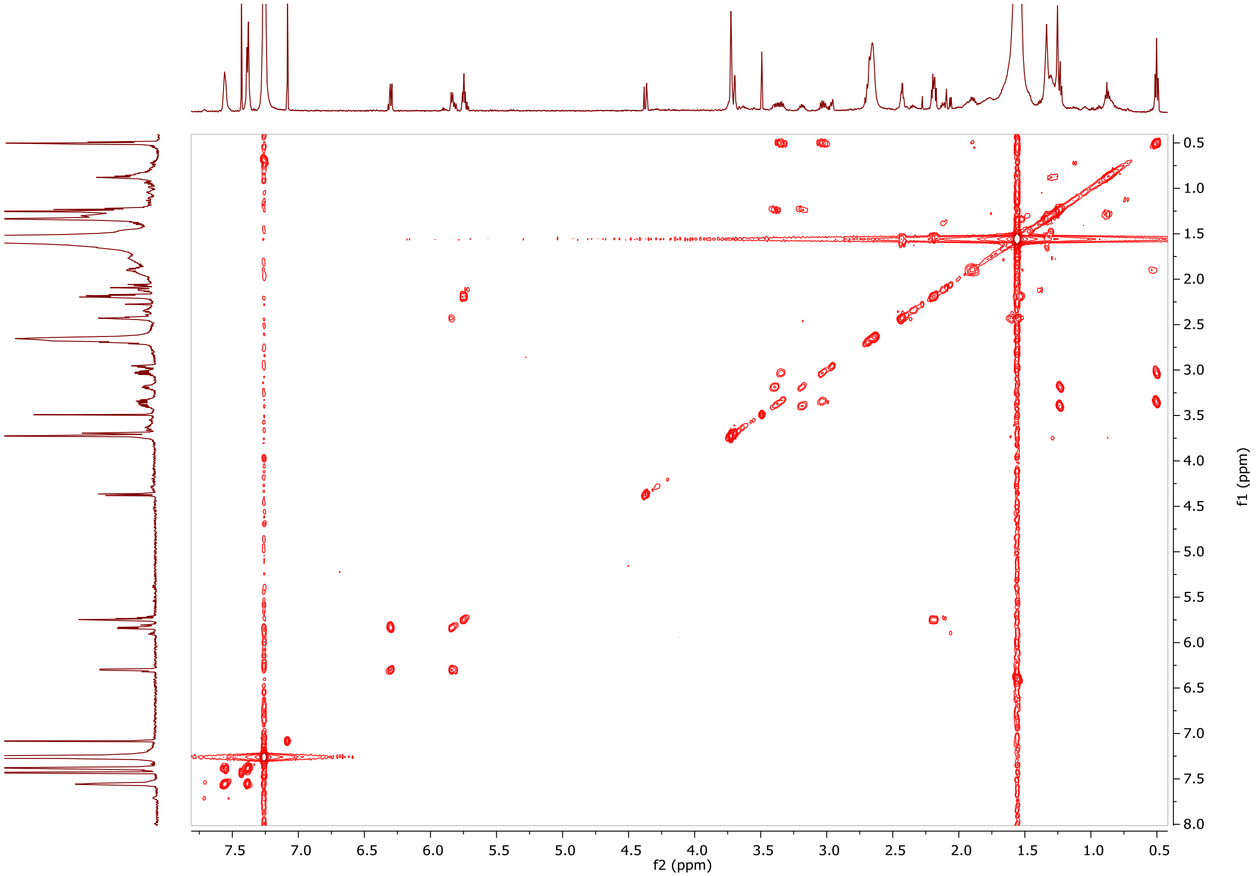


**Figure S20**. COSY (600 MHz, $CDCl_3$) of the (*S*)-MTPA amide (**6a**).

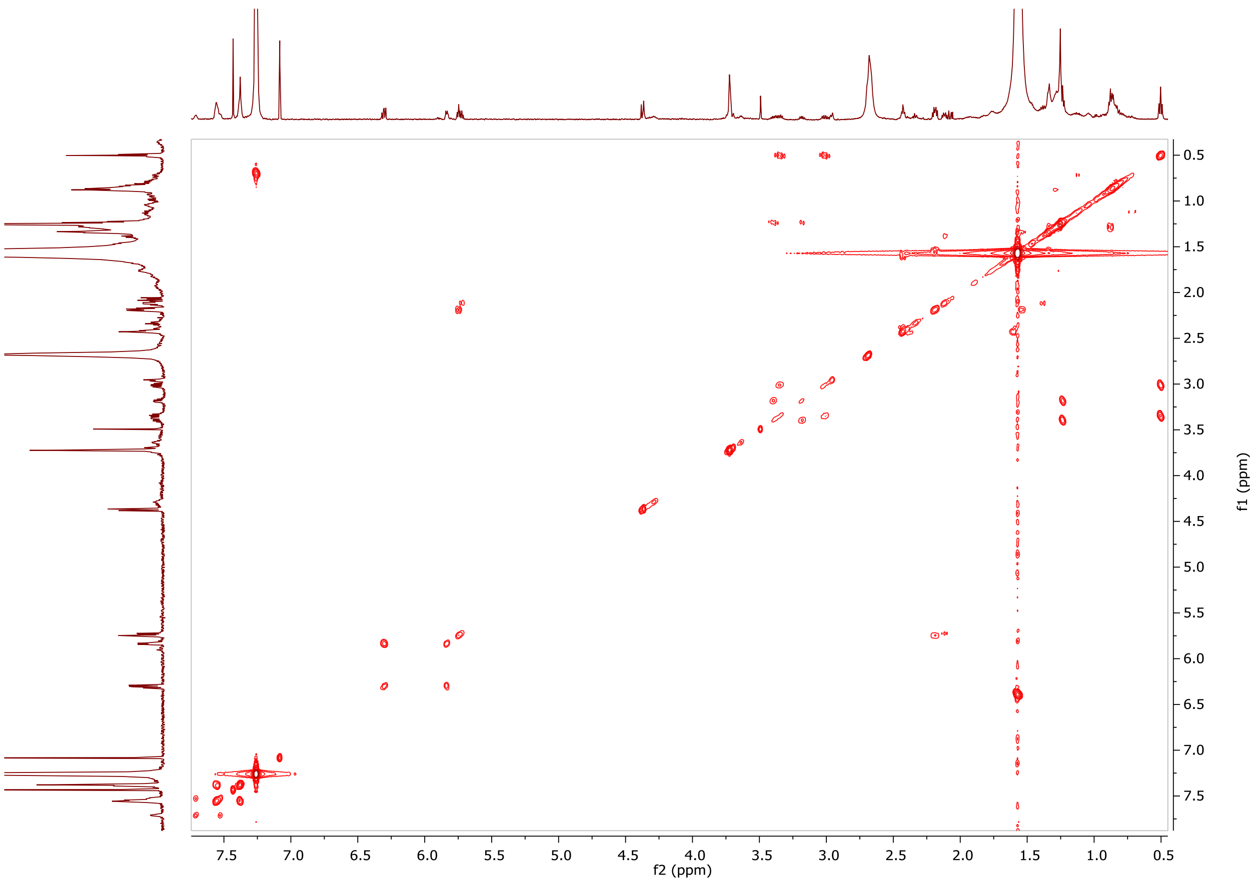


**Figure S21**. COSY (600 MHz, $CDCl_3$) of the (*R*)-MTPA amide (**6b**).

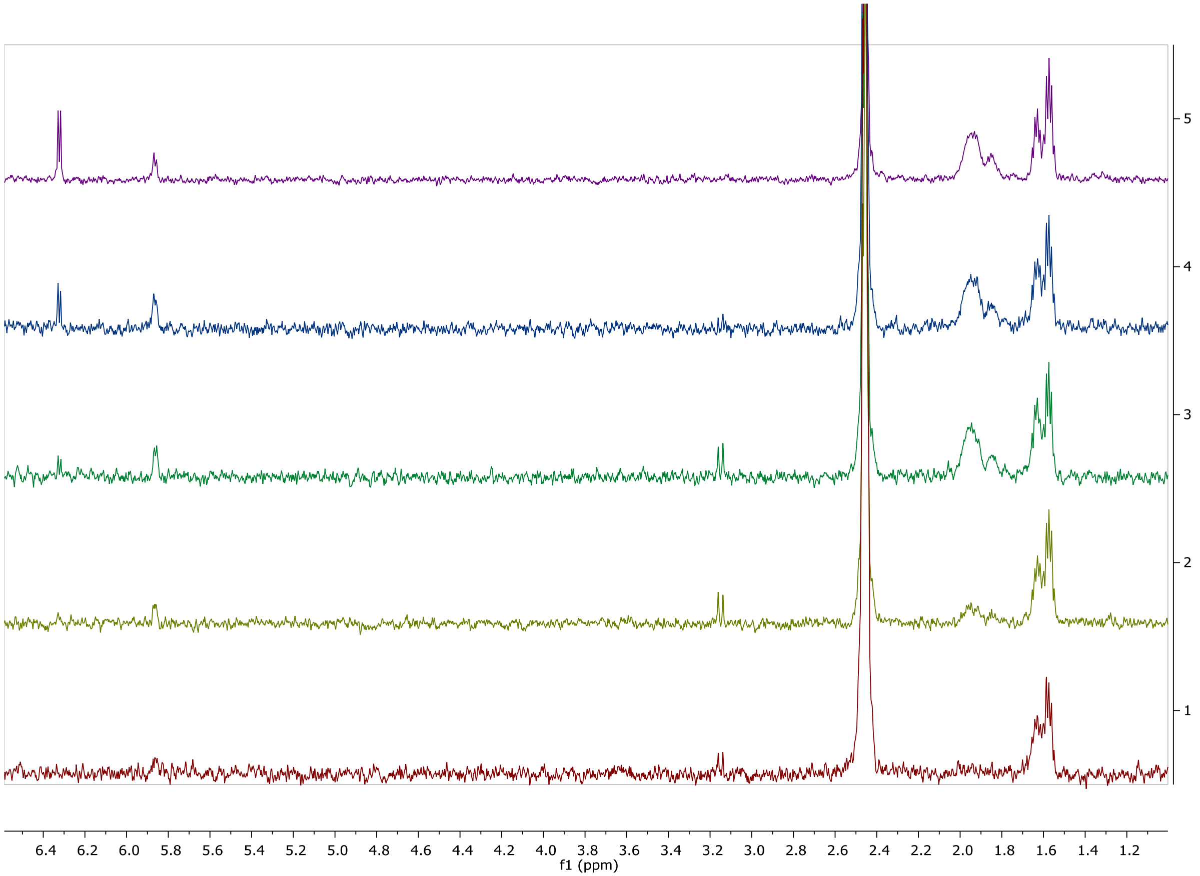


**Figure S22**. 1D TOCSY irradiating H-5 of the (*S*)-MTPA amide (**6a**) at 80 ms, 60 ms, 45 ms, 30 ms, and 15 ms (bottom to top)

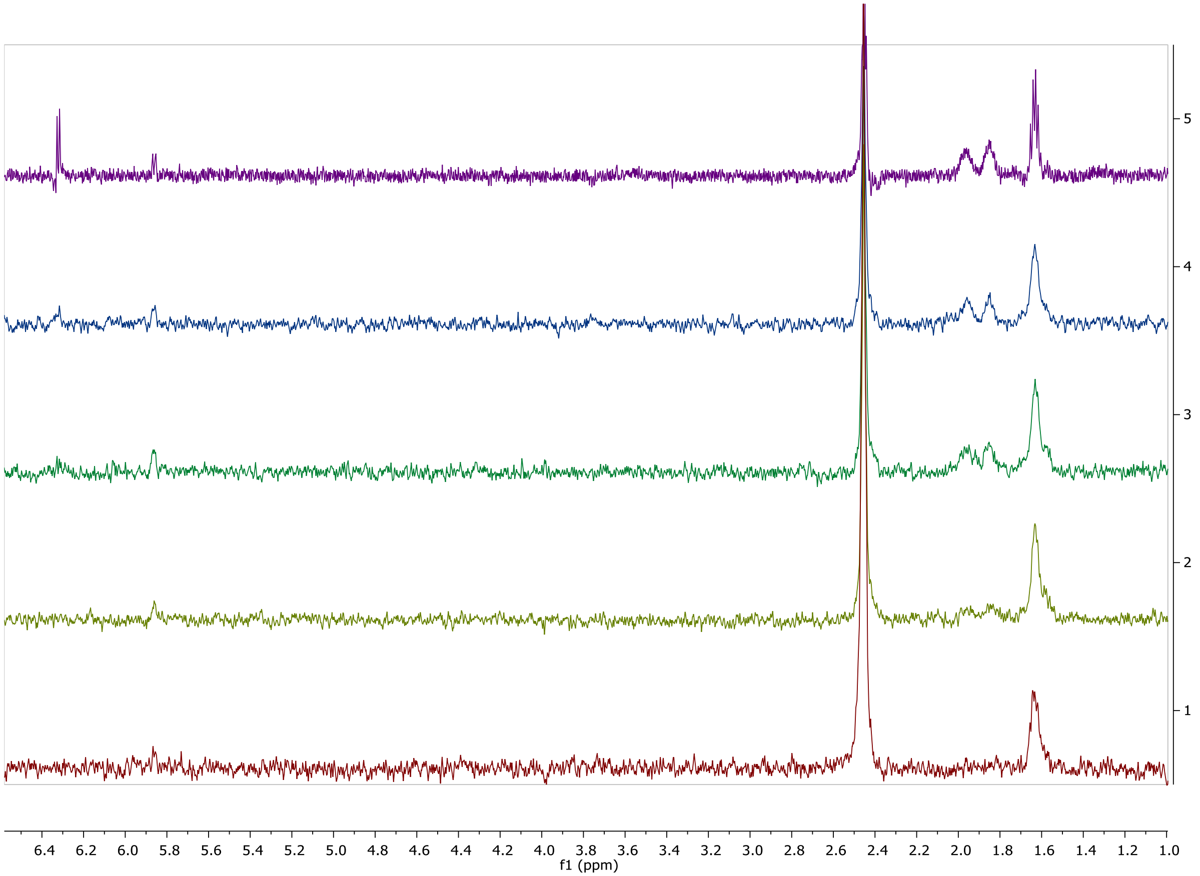


**Figure S23**. 1D TOCSY irradiating H-5 of the (*R*)-MTPA amide (**6b**) at 80 ms, 60 ms, 45 ms, 30 ms, and 15 ms (bottom to top).

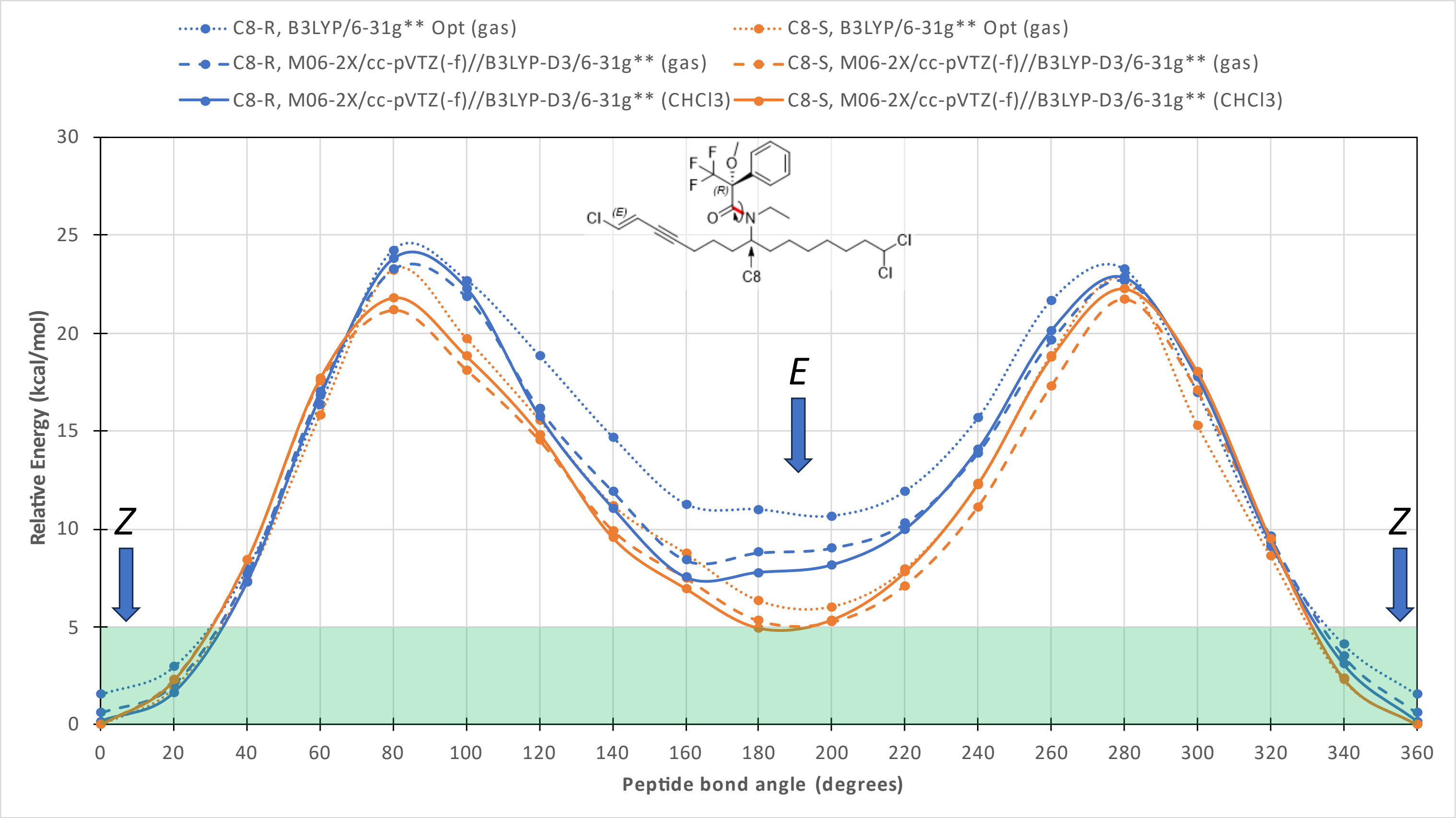


**Figure S24**. Results of DFT coordinate scan for the preferred configuration at the amide bond of the (*R*)-MTPA derivative of taveuniamide F. Both possibilities for the configuration of C8 were considered. The green shading indicates the thermally accessible energy range at room temperature (approximately 5 kcal/mol).

**Figure S25.** Results of Taveuniamide F (**4**) in GPCR MAX Panel Primary screen (β-arrestin) at 20 µM in agonist and antagonist mode

| GPCR ID | Control 1 | Mean RLU | SD | %CV | Control 2 | Mean RLU | SD | %CV | Assay Mode | Rep 1 RLU | Rep 2 RLU | Mean RLU | SD | % Activity |
|---|---|---|---|---|---|---|---|---|---|---|---|---|---|---|
| ADCYAP1R1 | Baseline | 48455.00 | 8172.4 | 0.169 | Max | 476480.00 | 23407.9 | 0.049 | Agonist | 53640 | 55040 | 54340.00 | 990 | 1.4 |
| ADORA3 | Baseline | 13725.00 | 800.4 | 0.058 | Max | 58280.00 | 4382.6 | 0.075 | Agonist | 12600 | 13120 | 12860.00 | 367.7 | -1.9 |
| ADRA1B | Baseline | 58745.00 | 6223 | 0.106 | Max | 476710.00 | 34646.7 | 0.073 | Agonist | 48760 | 48520 | 48640.00 | 169.7 | -2.4 |
| ADRA2A | Baseline | 56390.00 | 5469.2 | 0.097 | Max | 146810.00 | 4133.8 | 0.028 | Agonist | 61120 | 58240 | 59680.00 | 2036.5 | 3.6 |
| ADRA2B | Baseline | 11975.00 | 1578.4 | 0.132 | Max | 85490.00 | 6551.8 | 0.077 | Agonist | 36960 | 33400 | 35180.00 | 2517.3 | 31.6 |
| ADRA2C | Baseline | 25345.00 | 3078.3 | 0.121 | Max | 145830.00 | 12233.8 | 0.084 | Agonist | 34640 | 33680 | 34160.00 | 678.8 | 7.3 |
| ADRB1 | Baseline | 29773.33 | 6177.2 | 0.207 | Max | 107980.00 | 9560.7 | 0.089 | Agonist | 28680 | 27200 | 27940.00 | 1046.5 | -2.3 |
| ADRB2 | Baseline | 14160.00 | 5769.4 | 0.407 | Max | 85670.00 | 7898.8 | 0.092 | Agonist | 20120 | 19040 | 19580.00 | 763.7 | 7.6 |
| AGTR1 | Baseline | 131700.00 | 11668.6 | 0.089 | Max | 527280.00 | 35677.6 | 0.068 | Agonist | 141760 | 145320 | 143540.00 | 2517.3 | 3 |
| AGTRL1 | Baseline | 65185.00 | 7297.2 | 0.112 | Max | 323080.00 | 29207.1 | 0.09 | Agonist | 55880 | 57480 | 56680.00 | 1131.4 | -3.3 |
| AVPR1A | Baseline | 6135.00 | 819.5 | 0.134 | Max | 92580.00 | 5198.4 | 0.056 | Agonist | 5360 | 4720 | 5040.00 | 452.6 | -1.3 |
| AVPR1B | Baseline | 3865.00 | 607.7 | 0.157 | Max | 35340.00 | 2784.8 | 0.079 | Agonist | 3600 | 4600 | 4100.00 | 707.1 | 0.7 |
| AVPR2 | Baseline | 96240.00 | 11511.2 | 0.12 | Max | 438250.00 | 41276.8 | 0.094 | Agonist | 92000 | 93200 | 92600.00 | 848.5 | -1.1 |
| BDKRB1 | Baseline | 3765.00 | 519.1 | 0.138 | Max | 27660.00 | 2108.1 | 0.076 | Agonist | 2920 | 3440 | 3180.00 | 367.7 | -2.4 |
| BDKRB2 | Baseline | 157840.00 | 17681.3 | 0.112 | Max | 741100.00 | 57942.2 | 0.078 | Agonist | 143000 | 144240 | 143620.00 | 876.8 | -2.4 |
| BRS3 | Baseline | 68210.00 | 10803.1 | 0.158 | Max | 286270.00 | 22749.2 | 0.079 | Agonist | 60960 | 62000 | 61480.00 | 735.4 | -3.1 |
| C3AR1 | Baseline | 7025.00 | 1182.1 | 0.168 | Max | 260740.00 | 36854.4 | 0.141 | Agonist | 6520 | 7280 | 6900.00 | 537.4 | 0 |
| C5aR1 | Baseline | 22190.00 | 1610.4 | 0.073 | Max | 272890.00 | 15421.4 | 0.057 | Agonist | 16560 | 16800 | 16680.00 | 169.7 | -2.2 |
| C5L2 | Baseline | 56200.00 | 3618.9 | 0.064 | Max | 166520.00 | 15394.4 | 0.092 | Agonist | 55040 | 64040 | 59540.00 | 6364 | 3 |
| CALCR | Baseline | 6145.00 | 397.1 | 0.065 | Max | 28170.00 | 2917.6 | 0.104 | Agonist | 4960 | 5000 | 4980.00 | 28.3 | -5.3 |
| CALCRL-RAMP1 | Baseline | 10360.00 | 1635.5 | 0.158 | Max | 128370.00 | 9444.8 | 0.074 | Agonist | 8840 | 10480 | 9660.00 | 1159.7 | -0.6 |
| CALCRL-RAMP2 | Baseline | 240725.00 | 17081.4 | 0.071 | Max | 810580.00 | 33357.2 | 0.041 | Agonist | 207960 | 222440 | 215200.00 | 10238.9 | -4.5 |
| CALCRL-RAMP3 | Baseline | 25505.00 | 3852.8 | 0.151 | Max | 298400.00 | 31137.1 | 0.104 | Agonist | 21120 | 25080 | 23100.00 | 2800.1 | -0.9 |
| CALCR-RAMP2 | Baseline | 9425.00 | 1506.6 | 0.16 | Max | 53090.00 | 2352 | 0.044 | Agonist | 9120 | 9320 | 9220.00 | 141.4 | -0.5 |
| CALCR-RAMP3 | Baseline | 2200.00 | 219.1 | 0.1 | Max | 6480.00 | 250.9 | 0.039 | Agonist | 1920 | 1880 | 1900.00 | 28.3 | -7 |

| | | | | | | | | | | | | | | |
|---|---|---|---|---|---|---|---|---|---|---|---|---|---|---|
| CCKAR | Baseline | 6970.00 | 903.3 | 0.13 | Max | 96290.00 | 4141.7 | 0.043 | Agonist | 5880 | 7200 | 6540.00 | 933.4 | -0.5 |
| CCKBR | Baseline | 133635.00 | 14906.3 | 0.112 | Max | 437560.00 | 9533.7 | 0.022 | Agonist | 146360 | 151000 | 148680.00 | 3281 | 5 |
| CCR1 | Baseline | 202600.00 | 10293.4 | 0.051 | Max | 362733.33 | 12049 | 0.033 | Agonist | 170560 | 178640 | 174600.00 | 5713.4 | -17.5 |
| CCR10 | Baseline | 4585.00 | 555.9 | 0.121 | Max | 54220.00 | 5307.6 | 0.098 | Agonist | 3160 | 3880 | 3520.00 | 509.1 | -2.1 |
| CCR2 | Baseline | 8990.00 | 1478.5 | 0.164 | Max | 164080.00 | 10292.9 | 0.063 | Agonist | 6360 | 7440 | 6900.00 | 763.7 | -1.3 |
| CCR3 | Baseline | 24045.00 | 2346.4 | 0.098 | Max | 67840.00 | 4755.9 | 0.07 | Agonist | 20960 | 20320 | 20640.00 | 452.6 | -7.8 |
| CCR4 | Baseline | 31690.00 | 6179.2 | 0.195 | Max | 344000.00 | 37277.4 | 0.108 | Agonist | 29560 | 28920 | 29240.00 | 452.6 | -0.8 |
| CCR5 | Baseline | 7405.00 | 1291.3 | 0.174 | Max | 163030.00 | 8707.4 | 0.053 | Agonist | 6000 | 6000 | 6000.00 | 0 | -0.9 |
| CCR6 | Baseline | 9625.00 | 1376.5 | 0.143 | Max | 134000.00 | 5976 | 0.045 | Agonist | 11320 | 12240 | 11780.00 | 650.5 | 1.7 |
| CCR7 | Baseline | 194430.00 | 12963.9 | 0.067 | Max | 811160.00 | 52830.4 | 0.065 | Agonist | 178800 | 172200 | 175500.00 | 4666.9 | -3.1 |
| CCR8 | Baseline | 3875.00 | 407 | 0.105 | Max | 132560.00 | 10424 | 0.079 | Agonist | 2120 | 2360 | 2240.00 | 169.7 | -1.3 |
| CCR9 | Baseline | 9390.00 | 1456.7 | 0.155 | Max | 256980.00 | 37816.1 | 0.147 | Agonist | 4040 | 4520 | 4280.00 | 339.4 | -2.1 |
| CHRM1 | Baseline | 30595.00 | 3665.1 | 0.12 | Max | 95640.00 | 6499.2 | 0.068 | Agonist | 22000 | 22560 | 22280.00 | 396 | -12.8 |
| CHRM2 | Baseline | 20840.00 | 3378.2 | 0.162 | Max | 147780.00 | 13905.2 | 0.094 | Agonist | 21440 | 20240 | 20840.00 | 848.5 | 0 |
| CHRM3 | Baseline | 11890.00 | 1913.8 | 0.161 | Max | 51506.67 | 2491.7 | 0.048 | Agonist | 11200 | 11560 | 11380.00 | 254.6 | -1.3 |
| CHRM4 | Baseline | 35520.00 | 2549.3 | 0.072 | Max | 95910.00 | 6155.2 | 0.064 | Agonist | 26520 | 26000 | 26260.00 | 367.7 | -15.3 |
| CHRM5 | Baseline | 298306.67 | 29897.9 | 0.1 | Max | 748550.00 | 45923.8 | 0.061 | Agonist | 244400 | 244960 | 244680.00 | 396 | -11.9 |
| CMKLR1 | Baseline | 20775.00 | 2241.7 | 0.108 | Max | 308390.00 | 18748.1 | 0.061 | Agonist | 21480 | 20240 | 20860.00 | 876.8 | 0 |
| CNR1 | Baseline | 10730.00 | 2086.7 | 0.194 | Max | 108010.00 | 8658.7 | 0.08 | Agonist | 11000 | 10640 | 10820.00 | 254.6 | 0.1 |
| CNR2 | Baseline | 68815.00 | 7746.6 | 0.113 | Max | 158906.67 | 5397.7 | 0.034 | Agonist | 50920 | 54200 | 52560.00 | 2319.3 | -18 |
| CRHR1 | Baseline | 51755.00 | 7468 | 0.144 | Max | 643040.00 | 59389.6 | 0.092 | Agonist | 43240 | 43920 | 43580.00 | 480.8 | -1.4 |
| CRHR2 | Baseline | 13645.00 | 1893 | 0.139 | Max | 381810.00 | 38733.3 | 0.101 | Agonist | 11360 | 13240 | 12300.00 | 1329.4 | -0.4 |
| CRTH2 | Baseline | 20290.00 | 1506.7 | 0.074 | Max | 115370.00 | 8278.4 | 0.072 | Agonist | 18720 | 21200 | 19960.00 | 1753.6 | -0.3 |
| CX3CR1 | Baseline | 78110.00 | 7825 | 0.1 | Max | 1259580.00 | 56995.3 | 0.045 | Agonist | 55720 | 56520 | 56120.00 | 565.7 | -1.9 |
| CXCR1 | Baseline | 8015.00 | 1298.8 | 0.162 | Max | 181400.00 | 12026.7 | 0.066 | Agonist | 6400 | 8120 | 7260.00 | 1216.2 | -0.4 |
| CXCR2 | Baseline | 30225.00 | 2298.3 | 0.076 | Max | 206060.00 | 11417.4 | 0.055 | Agonist | 28040 | 24640 | 26340.00 | 2404.2 | -2.2 |
| CXCR3 | Baseline | 41720.00 | 4595.8 | 0.11 | Max | 126946.67 | 8894.4 | 0.07 | Agonist | 39240 | 39120 | 39180.00 | 84.8 | -3 |
| CXCR4 | Baseline | 4775.00 | 532.4 | 0.111 | Max | 19653.33 | 1575 | 0.08 | Agonist | 4720 | 4440 | 4580.00 | 198 | -1.3 |
| CXCR5 | Baseline | 16165.00 | 1385.6 | 0.086 | Max | 72410.00 | 6365.4 | 0.088 | Agonist | 13920 | 16440 | 15180.00 | 1781.9 | -1.8 |
| CXCR6 | Baseline | 800.00 | 145 | 0.181 | Max | 5720.00 | 360.7 | 0.063 | Agonist | 1040 | 1040 | 1040.00 | 0 | 4.9 |

| | | | | | | | | | | | | | | |
|---|---|---|---|---|---|---|---|---|---|---|---|---|---|---|
| CXCR7 | Baseline | 21475.00 | 1376.1 | 0.064 | Max | 148030.00 | 3033.4 | 0.02 | Agonist | 20960 | 20960 | 20960.00 | 0 | -0.4 |
| DRD1 | Baseline | 5505.00 | 758.6 | 0.138 | Max | 91970.00 | 12562.1 | 0.137 | Agonist | 5760 | 5240 | 5500.00 | 367.7 | 0 |
| DRD2L | Baseline | 8090.00 | 789.9 | 0.098 | Max | 42500.00 | 3713.3 | 0.087 | Agonist | 7840 | 8560 | 8200.00 | 509.1 | 0.3 |
| DRD2S | Baseline | 31560.00 | 4041 | 0.128 | Max | 166980.00 | 8897.1 | 0.053 | Agonist | 31200 | 32880 | 32040.00 | 1187.9 | 0.4 |
| DRD3 | Baseline | 37573.33 | 5119.6 | 0.136 | Max | 119610.00 | 4432 | 0.037 | Agonist | 38320 | 36960 | 37640.00 | 961.7 | 0.1 |
| DRD4 | Baseline | 1435.00 | 162 | 0.113 | Max | 8830.00 | 277.8 | 0.031 | Agonist | 1640 | 1280 | 1460.00 | 254.6 | 0.3 |
| DRD5 | Baseline | 4765.00 | 832.3 | 0.175 | Max | 64370.00 | 2501.1 | 0.039 | Agonist | 8240 | 8280 | 8260.00 | 28.3 | 5.9 |
| EBI2 | Baseline | 73285.00 | 6612.4 | 0.09 | Max | 934050.00 | 38150.7 | 0.041 | Agonist | 51600 | 50280 | 50940.00 | 933.4 | -2.6 |
| EDG1 | Baseline | 6235.00 | 954.4 | 0.153 | Max | 90510.00 | 11112.7 | 0.123 | Agonist | 8120 | 9560 | 8840.00 | 1018.2 | 3.1 |
| EDG3 | Baseline | 213755.00 | 37042.8 | 0.173 | Max | 1180390.00 | 54040.5 | 0.046 | Agonist | 166840 | 164440 | 165640.00 | 1697.1 | -5 |
| EDG4 | Baseline | 148775.00 | 10759.3 | 0.072 | Max | 378960.00 | 32300.6 | 0.085 | Agonist | 137240 | 145200 | 141220.00 | 5628.6 | -3.3 |
| EDG5 | Baseline | 55855.00 | 8967.6 | 0.161 | Max | 650120.00 | 16484.1 | 0.025 | Agonist | 38600 | 38960 | 38780.00 | 254.6 | -2.9 |
| EDG6 | Baseline | 49355.00 | 3594.8 | 0.073 | Max | 106120.00 | 3518 | 0.033 | Agonist | 54720 | 57800 | 56260.00 | 2177.9 | 12.2 |
| EDG7 | Baseline | 78730.00 | 12251.4 | 0.156 | Max | 400750.00 | 10072.4 | 0.025 | Agonist | 117520 | 119160 | 118340.00 | 1159.7 | 12.3 |
| EDNRA | Baseline | 2820.00 | 307.6 | 0.109 | Max | 47850.00 | 1843.9 | 0.039 | Agonist | 2600 | 2680 | 2640.00 | 56.6 | -0.4 |
| EDNRB | Baseline | 7520.00 | 719 | 0.096 | Max | 112280.00 | 8494.1 | 0.076 | Agonist | 6000 | 6120 | 6060.00 | 84.8 | -1.4 |
| F2R | Baseline | 4210.00 | 1205.1 | 0.286 | Max | 48720.00 | 6982 | 0.143 | Agonist | 6080 | 6920 | 6500.00 | 594 | 5.1 |
| F2RL1 | Baseline | 137280.00 | 8748 | 0.064 | Max | 604070.00 | 46341 | 0.077 | Agonist | 144680 | 146640 | 145660.00 | 1385.9 | 1.8 |
| F2RL3 | Baseline | 234745.00 | 9614.2 | 0.041 | Max | 605700.00 | 25682.7 | 0.042 | Agonist | 251240 | 239760 | 245500.00 | 8117.6 | 2.9 |
| FFAR1 | Baseline | 29440.00 | 1713.4 | 0.058 | Max | 82530.00 | 6397 | 0.078 | Agonist | 29000 | 29560 | 29280.00 | 396 | -0.3 |
| FPR1 | Baseline | 126415.00 | 12685.5 | 0.1 | Max | 445390.00 | 31902.7 | 0.072 | Agonist | 132440 | 129880 | 131160.00 | 1810.2 | 1.5 |
| FPRL1 | Baseline | 9800.00 | 711.1 | 0.073 | Max | 274620.00 | 17632.2 | 0.064 | Agonist | 10320 | 9000 | 9660.00 | 933.4 | -0.1 |
| FSHR | Baseline | 7725.00 | 544.4 | 0.07 | Max | 42130.00 | 4273.1 | 0.101 | Agonist | 7160 | 7360 | 7260.00 | 141.4 | -1.4 |
| GALR1 | Baseline | 117125.00 | 16278 | 0.139 | Max | 731290.00 | 32376.5 | 0.044 | Agonist | 86680 | 86440 | 86560.00 | 169.7 | -5 |
| GALR2 | Baseline | 76130.00 | 6887.4 | 0.09 | Max | 168420.00 | 4693.3 | 0.028 | Agonist | 73480 | 79000 | 76240.00 | 3903.2 | 0.1 |
| GCGR | Baseline | 32320.00 | 2272.6 | 0.07 | Max | 307560.00 | 8233.1 | 0.027 | Agonist | 44840 | 39560 | 42200.00 | 3733.5 | 3.6 |
| GHSR | Baseline | 49220.00 | 5747.4 | 0.117 | Max | 183490.00 | 13491.6 | 0.074 | Agonist | 45480 | 48560 | 47020.00 | 2177.9 | -1.6 |
| GIPR | Baseline | 680.00 | 88.2 | 0.13 | Max | 4510.00 | 535 | 0.119 | Agonist | 400 | 560 | 480.00 | 113.1 | -5.2 |
| GLP1R | Baseline | 5190.00 | 992.3 | 0.191 | Max | 212790.00 | 20291.5 | 0.095 | Agonist | 4240 | 4040 | 4140.00 | 141.4 | -0.5 |
| GLP2R | Baseline | 8440.00 | 1364.9 | 0.162 | Max | 65270.00 | 2579.6 | 0.04 | Agonist | 8560 | 9760 | 9160.00 | 848.5 | 1.3 |

| | | | | | | | | | | | | | | |
|---|---|---|---|---|---|---|---|---|---|---|---|---|---|---|
| GPR1 | Baseline | 8595.00 | 675.3 | 0.079 | Max | 143240.00 | 3199.3 | 0.022 | Agonist | 6960 | 7320 | 7140.00 | 254.6 | -1.1 |
| GPR103 | Baseline | 7120.00 | 537.5 | 0.075 | Max | 19480.00 | 1519 | 0.078 | Agonist | 5400 | 5160 | 5280.00 | 169.7 | -14.9 |
| GPR109A | Baseline | 48250.00 | 4465.6 | 0.093 | Max | 202900.00 | 13075.6 | 0.064 | Agonist | 45480 | 48200 | 46840.00 | 1923.3 | -0.9 |
| GPR109B | Baseline | 59180.00 | 6502.9 | 0.11 | Max | 485300.00 | 23787.1 | 0.049 | Agonist | 42320 | 46720 | 44520.00 | 3111.3 | -3.4 |
| GPR119 | Baseline | 41740.00 | 3222.5 | 0.077 | Max | 84450.00 | 1687.1 | 0.02 | Agonist | 36480 | 37920 | 37200.00 | 1018.2 | -10.6 |
| GPR120 | Baseline | 5360.00 | 607.8 | 0.113 | Max | 20550.00 | 1502.8 | 0.073 | Agonist | 3280 | 2920 | 3100.00 | 254.6 | -14.9 |
| GPR35 | Baseline | 12600.00 | 2040.8 | 0.162 | Max | 98350.00 | 6179.1 | 0.063 | Agonist | 10760 | 10680 | 10720.00 | 56.6 | -2.2 |
| GPR92 | Baseline | 91360.00 | 11602.1 | 0.127 | Max | 250240.00 | 10830.2 | 0.043 | Agonist | 89200 | 90000 | 89600.00 | 565.7 | -1.1 |
| GRPR | Baseline | 5005.00 | 572.3 | 0.114 | Max | 115450.00 | 7294.2 | 0.063 | Agonist | 3960 | 4640 | 4300.00 | 480.8 | -0.6 |
| HCRTR1 | Baseline | 12040.00 | 1724.2 | 0.143 | Max | 349540.00 | 36498.8 | 0.104 | Agonist | 13040 | 12880 | 12960.00 | 113.1 | 0.3 |
| HCRTR2 | Baseline | 5555.00 | 1060.1 | 0.191 | Max | 497870.00 | 32276.8 | 0.065 | Agonist | 6200 | 5800 | 6000.00 | 282.8 | 0.1 |
| HRH1 | Baseline | 49585.00 | 4519.1 | 0.091 | Max | 181180.00 | 12977.8 | 0.072 | Agonist | 46320 | 49320 | 47820.00 | 2121.3 | -1.3 |
| HRH2 | Baseline | 11720.00 | 1363.4 | 0.116 | Max | 38293.33 | 2758.9 | 0.072 | Agonist | 10240 | 10680 | 10460.00 | 311.1 | -4.7 |
| HRH3 | Baseline | 4490.00 | 534.8 | 0.119 | Max | 29220.00 | 1686.2 | 0.058 | Agonist | 3640 | 4520 | 4080.00 | 622.2 | -1.7 |
| HRH4 | Baseline | 33580.00 | 2426.5 | 0.072 | Max | 147290.00 | 3252.3 | 0.022 | Agonist | 31880 | 29400 | 30640.00 | 1753.6 | -2.6 |
| HTR1A | Baseline | 177580.00 | 24198 | 0.136 | Max | 689960.00 | 49142.8 | 0.071 | Agonist | 155640 | 178760 | 167200.00 | 16348.3 | -2 |
| HTR1B | Baseline | 48313.33 | 1464.3 | 0.03 | Max | 131333.33 | 12112.8 | 0.092 | Agonist | 47320 | 48880 | 48100.00 | 1103.1 | -0.3 |
| HTR1E | Baseline | 5828.57 | 422.5 | 0.072 | Max | 14840.00 | 1109.2 | 0.075 | Agonist | 4840 | 5040 | 4940.00 | 141.4 | -9.9 |
| HTR1F | Baseline | 13348.57 | 864.3 | 0.065 | Max | 40226.67 | 3456.3 | 0.086 | Agonist | 12200 | 11680 | 11940.00 | 367.7 | -5.2 |
| HTR2A | Baseline | 40855.00 | 6454.4 | 0.158 | Max | 285440.00 | 17703.1 | 0.062 | Agonist | 29960 | 28000 | 28980.00 | 1385.9 | -4.9 |
| HTR2C | Baseline | 74966.67 | 6137.6 | 0.082 | Max | 181160.00 | 11558.8 | 0.064 | Agonist | 70840 | 70920 | 70880.00 | 56.6 | -3.8 |
| HTR5A | Baseline | 66653.33 | 9131 | 0.137 | Max | 276500.00 | 24512 | 0.089 | Agonist | 58000 | 56200 | 57100.00 | 1272.8 | -4.6 |
| KISS1R | Baseline | 5640.00 | 811.1 | 0.144 | Max | 32500.00 | 995.2 | 0.031 | Agonist | 3880 | 3840 | 3860.00 | 28.3 | -6.6 |
| LHCGR | Baseline | 1650.00 | 264.2 | 0.16 | Max | 10920.00 | 56.6 | 0.005 | Agonist | 1200 | 1480 | 1340.00 | 198 | -3.3 |
| LTB4R | Baseline | 17770.00 | 2040 | 0.115 | Max | 174940.00 | 6873.1 | 0.039 | Agonist | 16840 | 15920 | 16380.00 | 650.5 | -0.9 |
| MC1R | Baseline | 1355.00 | 154.8 | 0.114 | Max | 5386.67 | 622.7 | 0.116 | Agonist | 920 | 1120 | 1020.00 | 141.4 | -8.3 |
| MC3R | Baseline | 1765.00 | 163.4 | 0.093 | Max | 8870.00 | 706.1 | 0.08 | Agonist | 1120 | 1040 | 1080.00 | 56.6 | -9.6 |
| MC4R | Baseline | 5370.00 | 921.6 | 0.172 | Max | 38050.00 | 2697.2 | 0.071 | Agonist | 4320 | 4040 | 4180.00 | 198 | -3.6 |
| MC5R | Baseline | 9335.00 | 608.1 | 0.065 | Max | 23960.00 | 1723.9 | 0.072 | Agonist | 8800 | 9400 | 9100.00 | 424.3 | -1.6 |
| MCHR1 | Baseline | 1240.00 | 205.1 | 0.165 | Max | 7620.00 | 311.1 | 0.041 | Agonist | 920 | 920 | 920.00 | 0 | -5 |

| | | | | | | | | | | | | | | |
|---|---|---|---|---|---|---|---|---|---|---|---|---|---|---|
| MCHR2 | Baseline | 8160.00 | 699.1 | 0.086 | Max | 61980.00 | 4047.2 | 0.065 | Agonist | 6080 | 6880 | 6480.00 | 565.7 | -3.1 |
| MLNR | Baseline | 15335.00 | 1588.5 | 0.104 | Max | 119100.00 | 3901.5 | 0.033 | Agonist | 15800 | 14800 | 15300.00 | 707.1 | 0 |
| MRGPRX1 | Baseline | 129240.00 | 8157.5 | 0.063 | Max | 398066.67 | 26576.8 | 0.067 | Agonist | 119120 | 118400 | 118760.00 | 509.1 | -3.9 |
| MRGPRX2 | Baseline | 34325.00 | 3647.3 | 0.106 | Max | 138800.00 | 10423.7 | 0.075 | Agonist | 29480 | 31560 | 30520.00 | 1470.8 | -3.6 |
| MTNR1A | Baseline | 5845.00 | 546.1 | 0.093 | Max | 21300.00 | 652 | 0.031 | Agonist | 4240 | 4600 | 4420.00 | 254.6 | -9.2 |
| NMBR | Baseline | 10420.00 | 796 | 0.076 | Max | 185470.00 | 14493.8 | 0.078 | Agonist | 8920 | 8600 | 8760.00 | 226.3 | -0.9 |
| NMU1R | Baseline | 8865.00 | 851.2 | 0.096 | Max | 135540.00 | 8982.5 | 0.066 | Agonist | 10240 | 9760 | 10000.00 | 339.4 | 0.9 |
| NPBWR1 | Baseline | 8833.33 | 585.4 | 0.066 | Max | 22546.67 | 2021.6 | 0.09 | Agonist | 7880 | 8280 | 8080.00 | 282.8 | -5.5 |
| NPBWR2 | Baseline | 10920.00 | 1029.8 | 0.094 | Max | 94360.00 | 6116 | 0.065 | Agonist | 8960 | 9560 | 9260.00 | 424.3 | -2 |
| NPFFR1 | Baseline | 7145.00 | 1438.4 | 0.201 | Max | 35826.67 | 2374 | 0.066 | Agonist | 5160 | 5680 | 5420.00 | 367.7 | -6 |
| NPSR1b | Baseline | 6380.00 | 704.3 | 0.11 | Max | 58550.00 | 2734.7 | 0.047 | Agonist | 4920 | 5240 | 5080.00 | 226.3 | -2.5 |
| NPY1R | Baseline | 21985.00 | 2480.5 | 0.113 | Max | 98830.00 | 6298.6 | 0.064 | Agonist | 19880 | 18920 | 19400.00 | 678.8 | -3.4 |
| NPY2R | Baseline | 25340.00 | 4057.2 | 0.16 | Max | 484220.00 | 22268.9 | 0.046 | Agonist | 22600 | 23240 | 22920.00 | 452.6 | -0.5 |
| NTSR1 | Baseline | 42885.00 | 3919.1 | 0.091 | Max | 176830.00 | 13815.8 | 0.078 | Agonist | 38080 | 39560 | 38820.00 | 1046.5 | -3 |
| OPRD1 | Baseline | 14980.00 | 977.2 | 0.065 | Max | 115630.00 | 5917.3 | 0.051 | Agonist | 12760 | 13280 | 13020.00 | 367.7 | -1.9 |
| OPRK1 | Baseline | 2195.00 | 370 | 0.169 | Max | 10210.00 | 567 | 0.056 | Agonist | 1600 | 1680 | 1640.00 | 56.6 | -6.9 |
| OPRL1 | Baseline | 37770.00 | 2578.2 | 0.068 | Max | 174950.00 | 6098 | 0.035 | Agonist | 35120 | 32440 | 33780.00 | 1895 | -2.9 |
| OPRM1 | Baseline | 24835.00 | 1861.3 | 0.075 | Max | 335400.00 | 32936.3 | 0.098 | Agonist | 20680 | 22320 | 21500.00 | 1159.7 | -1.1 |
| OXER1 | Baseline | 10665.00 | 757.4 | 0.071 | Max | 39520.00 | 1018.2 | 0.026 | Agonist | 9640 | 9520 | 9580.00 | 84.8 | -3.8 |
| OXTR | Baseline | 5510.00 | 310.4 | 0.056 | Max | 98580.00 | 2696.7 | 0.027 | Agonist | 4720 | 4720 | 4720.00 | 0 | -0.8 |
| P2RY1 | Baseline | 38020.00 | 2568.2 | 0.068 | Max | 108630.00 | 7690 | 0.071 | Agonist | 37400 | 37600 | 37500.00 | 141.4 | -0.7 |
| P2RY11 | Baseline | 5940.00 | 666.9 | 0.112 | Max | 56760.00 | 4072.9 | 0.072 | Agonist | 5120 | 5320 | 5220.00 | 141.4 | -1.4 |
| P2RY12 | Baseline | 14310.00 | 770.2 | 0.054 | Max | 128750.00 | 10019.9 | 0.078 | Agonist | 11480 | 12160 | 11820.00 | 480.8 | -2.2 |
| P2RY2 | Baseline | 56130.00 | 6387.1 | 0.114 | Max | 232700.00 | 13330.8 | 0.057 | Agonist | 71440 | 68120 | 69780.00 | 2347.6 | 7.7 |
| P2RY4 | Baseline | 54150.00 | 3440.1 | 0.064 | Max | 196280.00 | 17306.7 | 0.088 | Agonist | 56040 | 54080 | 55060.00 | 1385.9 | 0.6 |
| P2RY6 | Baseline | 55320.00 | 6618.5 | 0.12 | Max | 261990.00 | 19680.8 | 0.075 | Agonist | 116200 | 140920 | 128560.00 | 17479.7 | 35.4 |
| PPYR1 | Baseline | 4920.00 | 861.6 | 0.175 | Max | 57170.00 | 6062.4 | 0.106 | Agonist | 3920 | 3920 | 3920.00 | 0 | -1.9 |
| PRLHR | Baseline | 1580.00 | 193.6 | 0.123 | Max | 13560.00 | 1525.6 | 0.113 | Agonist | 1360 | 1200 | 1280.00 | 113.1 | -2.5 |
| PROKR1 | Baseline | 6785.00 | 1009.6 | 0.149 | Max | 88870.00 | 8237.6 | 0.093 | Agonist | 6600 | 5520 | 6060.00 | 763.7 | -0.9 |
| PROKR2 | Baseline | 1715.00 | 204.4 | 0.119 | Max | 7350.00 | 435 | 0.059 | Agonist | 1600 | 1720 | 1660.00 | 84.8 | -1 |

| | | | | | | | | | | | | | | |
|---|---|---|---|---|---|---|---|---|---|---|---|---|---|---|
| PTAFR | Baseline | 115470.00 | 12923.4 | 0.112 | Max | 542270.00 | 23979.3 | 0.044 | Agonist | 93160 | 92480 | 92820.00 | 480.8 | -5.3 |
| PTGER2 | Baseline | 2120.00 | 290.8 | 0.137 | Max | 12740.00 | 835.5 | 0.066 | Agonist | 1720 | 2080 | 1900.00 | 254.6 | -2.1 |
| PTGER3 | Baseline | 53680.00 | 5457.3 | 0.102 | Max | 171400.00 | 13649.1 | 0.08 | Agonist | 49680 | 53080 | 51380.00 | 2404.2 | -2 |
| PTGER4 | Baseline | 41730.00 | 4451.2 | 0.107 | Max | 165830.00 | 10930.5 | 0.066 | Agonist | 39760 | 41400 | 40580.00 | 1159.7 | -0.9 |
| PTGFR | Baseline | 825.00 | 97.8 | 0.119 | Max | 22973.33 | 2077.4 | 0.09 | Agonist | 680 | 680 | 680.00 | 0 | -0.7 |
| PTGIR | Baseline | 39465.00 | 3517.3 | 0.089 | Max | 118200.00 | 5572.2 | 0.047 | Agonist | 28160 | 28000 | 28080.00 | 113.1 | -14.5 |
| PTHR1 | Baseline | 30905.00 | 3143.3 | 0.102 | Max | 540020.00 | 31185.1 | 0.058 | Agonist | 28800 | 28000 | 28400.00 | 565.7 | -0.5 |
| PTHR2 | Baseline | 22515.00 | 3396.7 | 0.151 | Max | 373170.00 | 31305.7 | 0.084 | Agonist | 18360 | 19920 | 19140.00 | 1103.1 | -1 |
| RXFP3 | Baseline | 9040.00 | 831.7 | 0.092 | Max | 28590.00 | 2144.5 | 0.075 | Agonist | 7880 | 8280 | 8080.00 | 282.8 | -4.9 |
| SCTR | Baseline | 67840.00 | 9948.8 | 0.147 | Max | 434620.00 | 45014.2 | 0.104 | Agonist | 62200 | 62360 | 62280.00 | 113.1 | -1.5 |
| SSTR1 | Baseline | 1390.00 | 219.9 | 0.158 | Max | 5830.00 | 367.2 | 0.063 | Agonist | 960 | 1200 | 1080.00 | 169.7 | -7 |
| SSTR2 | Baseline | 735.00 | 111 | 0.151 | Max | 48690.00 | 3807 | 0.078 | Agonist | 760 | 800 | 780.00 | 28.3 | 0.1 |
| SSTR3 | Baseline | 9940.00 | 415.7 | 0.042 | Max | 68350.00 | 8435 | 0.123 | Agonist | 8120 | 9520 | 8820.00 | 990 | -1.9 |
| SSTR5 | Baseline | 11330.00 | 766.1 | 0.068 | Max | 77320.00 | 1768.8 | 0.023 | Agonist | 10360 | 12520 | 11440.00 | 1527.4 | 0.2 |
| TACR1 | Baseline | 62555.00 | 5013.8 | 0.08 | Max | 365010.00 | 18214.3 | 0.05 | Agonist | 54560 | 57240 | 55900.00 | 1895 | -2.2 |
| TACR2 | Baseline | 101895.00 | 11584.7 | 0.114 | Max | 396170.00 | 32159.9 | 0.081 | Agonist | 111160 | 100680 | 105920.00 | 7410.5 | 1.4 |
| TACR3 | Baseline | 11800.00 | 1535.7 | 0.13 | Max | 224430.00 | 13268.5 | 0.059 | Agonist | 9840 | 10440 | 10140.00 | 424.3 | -0.8 |
| TBXA2R | Baseline | 21150.00 | 1641.6 | 0.078 | Max | 96190.00 | 8526.3 | 0.089 | Agonist | 19080 | 22400 | 20740.00 | 2347.6 | -0.5 |
| TRHR | Baseline | 1380.00 | 326.4 | 0.237 | Max | 22400.00 | 487.7 | 0.022 | Agonist | 840 | 1000 | 920.00 | 113.1 | -2.2 |
| TSHR(L) | Baseline | 685.00 | 142.5 | 0.208 | Max | 8220.00 | 779.4 | 0.095 | Agonist | 520 | 560 | 540.00 | 28.3 | -1.9 |
| UTR2 | Baseline | 7365.00 | 425.7 | 0.058 | Max | 22170.00 | 1585.4 | 0.072 | Agonist | 5960 | 5680 | 5820.00 | 198 | -10.4 |
| VIPR1 | Baseline | 48970.00 | 6735.7 | 0.138 | Max | 506870.00 | 31114.9 | 0.061 | Agonist | 45000 | 46200 | 45600.00 | 848.5 | -0.7 |
| VIPR2 | Baseline | 13385.00 | 2362.1 | 0.176 | Max | 375280.00 | 24813.8 | 0.066 | Agonist | 11520 | 11360 | 11440.00 | 113.1 | -0.5 |
| ADCYAP1R1 | EC80 | 423837.50 | 22316.9 | 0.053 | Basal | 44205.00 | 6462.5 | 0.146 | Antagonist | 365600 | 356720 | 361160.00 | 6279.1 | 16.5 |
| ADORA3 | EC80 | 45735.00 | 1857.6 | 0.041 | Basal | 12565.00 | 922.5 | 0.073 | Antagonist | 45080 | 45720 | 45400.00 | 452.6 | 1 |
| ADRA1B | EC80 | 335063.33 | 23114.6 | 0.069 | Basal | 57095.00 | 8248.3 | 0.144 | Antagonist | 281800 | 261040 | 271420.00 | 14679.5 | 22.9 |
| ADRA2A | EC80 | 115402.50 | 8316.2 | 0.072 | Basal | 55520.00 | 3310.6 | 0.06 | Antagonist | 87760 | 92440 | 90100.00 | 3309.3 | 42.3 |
| ADRA2B | EC80 | 74473.33 | 6770.4 | 0.091 | Basal | 12200.00 | 1141.2 | 0.094 | Antagonist | 71440 | 64240 | 67840.00 | 5091.2 | 10.7 |
| ADRA2C | EC80 | 115636.67 | 10493.7 | 0.091 | Basal | 23925.00 | 1800.4 | 0.075 | Antagonist | 96240 | 89000 | 92620.00 | 5119.4 | 25.1 |
| ADRB1 | EC80 | 70425.00 | 4159.2 | 0.059 | Basal | 27220.00 | 2691.7 | 0.099 | Antagonist | 48080 | 47200 | 47640.00 | 622.2 | 52.7 |

| | | | | | | | | | | | | | | |
|---|---|---|---|---|---|---|---|---|---|---|---|---|---|---|
| ADRB2 | EC80 | 53325.00 | 3185.7 | 0.06 | Basal | 12400.00 | 3371.3 | 0.272 | Antagonist | 43840 | 42080 | 42960.00 | 1244.5 | 25.3 |
| AGTR1 | EC80 | 441076.67 | 27079.6 | 0.061 | Basal | 118730.00 | 10590 | 0.089 | Antagonist | 393480 | 338240 | 365860.00 | 39060.6 | 23.3 |
| AGTRL1 | EC80 | 244297.78 | 25384 | 0.104 | Basal | 58640.00 | 4268 | 0.073 | Antagonist | 243840 | 219360 | 231600.00 | 17310 | 6.8 |
| AVPR1A | EC80 | 83992.50 | 5761.3 | 0.069 | Basal | 5735.00 | 545.1 | 0.095 | Antagonist | 46040 | 46000 | 46020.00 | 28.3 | 48.5 |
| AVPR1B | EC80 | 26416.67 | 1577.4 | 0.06 | Basal | 3725.00 | 356.8 | 0.096 | Antagonist | 13280 | 13440 | 13360.00 | 113.1 | 57.5 |
| AVPR2 | EC80 | 408021.82 | 27400.5 | 0.067 | Basal | 81685.00 | 7214.9 | 0.088 | Antagonist | 370240 | 352120 | 361180.00 | 12812.8 | 14.4 |
| BDKRB1 | EC80 | 20095.00 | 864.6 | 0.043 | Basal | 3765.00 | 535.6 | 0.142 | Antagonist | 15920 | 15720 | 15820.00 | 141.4 | 26.2 |
| BDKRB2 | EC80 | 683960.00 | 47719.1 | 0.07 | Basal | 146745.00 | 13372.6 | 0.091 | Antagonist | 668520 | 609560 | 639040.00 | 41691 | 8.4 |
| BRS3 | EC80 | 189566.67 | 10867.3 | 0.057 | Basal | 59295.00 | 6518 | 0.11 | Antagonist | 169760 | 167880 | 168820.00 | 1329.4 | 15.9 |
| C3AR1 | EC80 | 201870.00 | 22608.2 | 0.112 | Basal | 7550.00 | 808.2 | 0.107 | Antagonist | 184960 | 178840 | 181900.00 | 4327.5 | 10.3 |
| C5aR1 | EC80 | 252640.00 | 17133.4 | 0.068 | Basal | 20500.00 | 1709.9 | 0.083 | Antagonist | 255160 | 243800 | 249480.00 | 8032.7 | 1.4 |
| C5L2 | EC80 | 149397.50 | 10012.6 | 0.067 | Basal | 54575.00 | 4111.8 | 0.075 | Antagonist | 127440 | 125560 | 126500.00 | 1329.4 | 24.1 |
| CALCR | EC80 | 22533.33 | 1841.7 | 0.082 | Basal | 5560.00 | 235.2 | 0.042 | Antagonist | 21120 | 19160 | 20140.00 | 1385.9 | 14.1 |
| CALCRL-RAMP1 | EC80 | 116753.33 | 5540.3 | 0.047 | Basal | 9555.00 | 899.6 | 0.094 | Antagonist | 82200 | 84400 | 83300.00 | 1555.6 | 31.2 |
| CALCRL-RAMP2 | EC80 | 727130.00 | 40944.4 | 0.056 | Basal | 234295.00 | 21364.1 | 0.091 | Antagonist | 672400 | 647640 | 660020.00 | 17508 | 13.6 |
| CALCRL-RAMP3 | EC80 | 285527.50 | 16504.6 | 0.058 | Basal | 31140.00 | 2294.9 | 0.074 | Antagonist | 234960 | 237880 | 236420.00 | 2064.8 | 19.3 |
| CALCR-RAMP2 | EC80 | 35923.33 | 2288.2 | 0.064 | Basal | 8340.00 | 572.1 | 0.069 | Antagonist | 19560 | 19960 | 19760.00 | 282.8 | 58.6 |
| CALCR-RAMP3 | EC80 | 4780.00 | 261.7 | 0.055 | Basal | 2160.00 | 161.4 | 0.075 | Antagonist | 4760 | 4640 | 4700.00 | 84.8 | 3.1 |
| CCKAR | EC80 | 69102.50 | 3841.4 | 0.056 | Basal | 7100.00 | 765 | 0.108 | Antagonist | 60320 | 64320 | 62320.00 | 2828.4 | 10.9 |
| CCKBR | EC80 | 438815.00 | 32448.5 | 0.074 | Basal | 122455.00 | 7736.5 | 0.063 | Antagonist | 420520 | 392520 | 406520.00 | 19799 | 10.2 |
| CCR1 | EC80 | 344914.67 | 11659.7 | 0.034 | Basal | 202930.00 | 8381.6 | 0.041 | Antagonist | 249840 | 253400 | 251620.00 | 2517.3 | 65.7 |
| CCR10 | EC80 | 48537.14 | 3100 | 0.064 | Basal | 4335.00 | 568.9 | 0.131 | Antagonist | 36000 | 31600 | 33800.00 | 3111.3 | 33.3 |
| CCR2 | EC80 | 136494.29 | 8308.4 | 0.061 | Basal | 9035.00 | 1068.9 | 0.118 | Antagonist | 129520 | 147920 | 138720.00 | 13010.8 | -1.7 |
| CCR3 | EC80 | 61717.50 | 3680.3 | 0.06 | Basal | 22290.00 | 1084.3 | 0.049 | Antagonist | 54560 | 53720 | 54140.00 | 594 | 19.2 |
| CCR4 | EC80 | 267815.00 | 15218.2 | 0.057 | Basal | 32690.00 | 3014.9 | 0.092 | Antagonist | 117320 | 141320 | 129320.00 | 16970.6 | 58.9 |
| CCR5 | EC80 | 125094.29 | 11801.5 | 0.094 | Basal | 7015.00 | 752.6 | 0.107 | Antagonist | 104880 | 110560 | 107720.00 | 4016.4 | 14.7 |
| CCR6 | EC80 | 126082.50 | 8242.5 | 0.065 | Basal | 9725.00 | 562.2 | 0.058 | Antagonist | 56800 | 59880 | 58340.00 | 2177.9 | 58.2 |
| CCR7 | EC80 | 701982.50 | 27220.2 | 0.039 | Basal | 189910.00 | 11727.9 | 0.062 | Antagonist | 661800 | 657880 | 659840.00 | 2771.9 | 8.2 |
| CCR8 | EC80 | 119222.86 | 6359.2 | 0.053 | Basal | 3700.00 | 250.3 | 0.068 | Antagonist | 72120 | 66960 | 69540.00 | 3648.7 | 43 |

| | | | | | | | | | | | | | | |
|---|---|---|---|---|---|---|---|---|---|---|---|---|---|---|
| CCR9 | EC80 | 197671.43 | 13649.6 | 0.069 | Basal | 9115.00 | 975.7 | 0.107 | Antagonist | 11520 | 10920 | 11220.00 | 424.3 | 98.9 |
| CHRM1 | EC80 | 73452.00 | 5070.3 | 0.069 | Basal | 26430.00 | 2451.9 | 0.093 | Antagonist | 57360 | 69000 | 63180.00 | 8230.7 | 21.8 |
| CHRM2 | EC80 | 97336.67 | 10757.4 | 0.111 | Basal | 17315.00 | 1261.4 | 0.073 | Antagonist | 69080 | 66800 | 67940.00 | 1612.2 | 36.7 |
| CHRM3 | EC80 | 43675.00 | 3294.8 | 0.075 | Basal | 11195.00 | 703.1 | 0.063 | Antagonist | 23320 | 25120 | 24220.00 | 1272.8 | 59.9 |
| CHRM4 | EC80 | 67905.00 | 4565.4 | 0.067 | Basal | 32840.00 | 3252.3 | 0.099 | Antagonist | 32600 | 34360 | 33480.00 | 1244.5 | 98.2 |
| CHRM5 | EC80 | 593597.50 | 28556 | 0.048 | Basal | 303653.33 | 19326.7 | 0.064 | Antagonist | 495440 | 508560 | 502000.00 | 9277.2 | 31.6 |
| CMKLR1 | EC80 | 268022.50 | 13770.7 | 0.051 | Basal | 21985.00 | 2120.8 | 0.096 | Antagonist | 217640 | 213120 | 215380.00 | 3196.1 | 21.4 |
| CNR1 | EC80 | 85346.67 | 8286 | 0.097 | Basal | 10945.00 | 1112 | 0.102 | Antagonist | 25200 | 20520 | 22860.00 | 3309.3 | 84 |
| CNR2 | EC80 | 126833.33 | 5553 | 0.044 | Basal | 61766.67 | 4825.2 | 0.078 | Antagonist | 98360 | 92120 | 95240.00 | 4412.4 | 48.6 |
| CRHR1 | EC80 | 551430.00 | 36651.1 | 0.066 | Basal | 44955.00 | 5872.2 | 0.131 | Antagonist | 554480 | 572720 | 563600.00 | 12897.6 | -2.4 |
| CRHR2 | EC80 | 352313.33 | 33963.5 | 0.096 | Basal | 12740.00 | 1642 | 0.129 | Antagonist | 337480 | 285840 | 311660.00 | 36515 | 12 |
| CRTH2 | EC80 | 96196.67 | 10524.9 | 0.109 | Basal | 18700.00 | 1632.5 | 0.087 | Antagonist | 85440 | 80760 | 83100.00 | 3309.3 | 16.9 |
| CX3CR1 | EC80 | 899682.50 | 43415.8 | 0.048 | Basal | 69140.00 | 4451.7 | 0.064 | Antagonist | 611560 | 597080 | 604320.00 | 10238.9 | 35.6 |
| CXCR1 | EC80 | 134005.00 | 16012 | 0.119 | Basal | 8205.00 | 741.4 | 0.09 | Antagonist | 105640 | 123960 | 114800.00 | 12954.2 | 15.3 |
| CXCR2 | EC80 | 140962.50 | 8618.7 | 0.061 | Basal | 29110.00 | 1894 | 0.065 | Antagonist | 115760 | 120280 | 118020.00 | 3196.1 | 20.5 |
| CXCR3 | EC80 | 117768.57 | 6046.2 | 0.051 | Basal | 41190.00 | 2352.9 | 0.057 | Antagonist | 116080 | 112440 | 114260.00 | 2573.9 | 4.6 |
| CXCR4 | EC80 | 13525.00 | 743.9 | 0.055 | Basal | 5114.29 | 550.1 | 0.108 | Antagonist | 10360 | 9320 | 9840.00 | 735.4 | 43.8 |
| CXCR5 | EC80 | 71762.50 | 5395.4 | 0.075 | Basal | 16110.00 | 1095.6 | 0.068 | Antagonist | 65960 | 71920 | 68940.00 | 4214.4 | 5.1 |
| CXCR6 | EC80 | 4537.14 | 456.8 | 0.101 | Basal | 740.00 | 146.6 | 0.198 | Antagonist | 2920 | 3640 | 3280.00 | 509.1 | 33.1 |
| CXCR7 | EC80 | 116335.00 | 10927.9 | 0.094 | Basal | 21795.00 | 3058 | 0.14 | Antagonist | 110480 | 115320 | 112900.00 | 3422.4 | 3.6 |
| DRD1 | EC80 | 56463.33 | 3593 | 0.064 | Basal | 5875.00 | 902.4 | 0.154 | Antagonist | 48880 | 45240 | 47060.00 | 2573.9 | 18.6 |
| DRD2L | EC80 | 29013.33 | 2350.5 | 0.081 | Basal | 7545.00 | 614.8 | 0.081 | Antagonist | 29240 | 27160 | 28200.00 | 1470.8 | 3.8 |
| DRD2S | EC80 | 120920.00 | 10324.9 | 0.085 | Basal | 27370.00 | 1975.8 | 0.072 | Antagonist | 127080 | 130000 | 128540.00 | 2064.8 | -8.1 |
| DRD3 | EC80 | 90502.86 | 4357.6 | 0.048 | Basal | 34005.71 | 3509 | 0.103 | Antagonist | 89200 | 80960 | 85080.00 | 5826.6 | 9.6 |
| DRD4 | EC80 | 5220.00 | 394.7 | 0.076 | Basal | 1380.00 | 196 | 0.142 | Antagonist | 4520 | 4320 | 4420.00 | 141.4 | 20.8 |
| DRD5 | EC80 | 43350.00 | 4800.9 | 0.111 | Basal | 4255.00 | 775 | 0.182 | Antagonist | 45120 | 39480 | 42300.00 | 3988.1 | 2.7 |
| EBI2 | EC80 | 662472.50 | 42533.8 | 0.064 | Basal | 70230.00 | 4475.2 | 0.064 | Antagonist | 586000 | 525200 | 555600.00 | 42992.1 | 18 |
| EDG1 | EC80 | 79217.50 | 9644.5 | 0.122 | Basal | 6310.00 | 1078.4 | 0.171 | Antagonist | 54800 | 55360 | 55080.00 | 396 | 33.1 |
| EDG3 | EC80 | 855536.67 | 59647.2 | 0.07 | Basal | 182340.00 | 25822.2 | 0.142 | Antagonist | 726840 | 701840 | 714340.00 | 17677.7 | 21 |
| EDG4 | EC80 | 282340.00 | 16940.7 | 0.06 | Basal | 122394.29 | 7387.4 | 0.06 | Antagonist | 200200 | 184400 | 192300.00 | 11172.3 | 56.3 |

| | | | | | | | | | | | | | | |
|---|---|---|---|---|---|---|---|---|---|---|---|---|---|---|
| EDG5 | EC80 | 527976.67 | 42337.7 | 0.08 | Basal | 51375.00 | 7620.5 | 0.148 | Antagonist | 537000 | 511520 | 524260.00 | 18017.1 | 0.8 |
| EDG6 | EC80 | 101812.50 | 3600.5 | 0.035 | Basal | 51000.00 | 2494.5 | 0.049 | Antagonist | 93400 | 90320 | 91860.00 | 2177.9 | 19.6 |
| EDG7 | EC80 | 288877.50 | 20897.2 | 0.072 | Basal | 66960.00 | 4768.8 | 0.071 | Antagonist | 180560 | 225880 | 203220.00 | 32046.1 | 38.6 |
| EDNRA | EC80 | 39845.00 | 2752.9 | 0.069 | Basal | 2710.00 | 428.6 | 0.158 | Antagonist | 26640 | 30280 | 28460.00 | 2573.9 | 30.7 |
| EDNRB | EC80 | 77350.00 | 7746.3 | 0.1 | Basal | 6825.00 | 591 | 0.087 | Antagonist | 68640 | 53640 | 61140.00 | 10606.6 | 23 |
| F2R | EC80 | 45786.67 | 6729.6 | 0.147 | Basal | 4345.00 | 1025.6 | 0.236 | Antagonist | 38160 | 43720 | 40940.00 | 3931.5 | 11.7 |
| F2RL1 | EC80 | 425740.00 | 25412 | 0.06 | Basal | 128160.00 | 11969.6 | 0.093 | Antagonist | 413960 | 477160 | 445560.00 | 44689.2 | -6.7 |
| F2RL3 | EC80 | 413546.67 | 8294.8 | 0.02 | Basal | 216060.00 | 14748.2 | 0.068 | Antagonist | 296880 | 296200 | 296540.00 | 480.8 | 59.2 |
| FFAR1 | EC80 | 79033.33 | 5619.9 | 0.071 | Basal | 30753.33 | 2144.2 | 0.07 | Antagonist | 57720 | 59800 | 58760.00 | 1470.8 | 42 |
| FPR1 | EC80 | 354696.67 | 23622.3 | 0.067 | Basal | 110240.00 | 10397.9 | 0.094 | Antagonist | 355160 | 377000 | 366080.00 | 15443.2 | -4.7 |
| FPRL1 | EC80 | 203580.00 | 13764.8 | 0.068 | Basal | 9565.00 | 797.6 | 0.083 | Antagonist | 202760 | 194640 | 198700.00 | 5741.7 | 2.5 |
| FSHR | EC80 | 31733.33 | 2559.9 | 0.081 | Basal | 6605.00 | 349.6 | 0.053 | Antagonist | 22320 | 20800 | 21560.00 | 1074.8 | 40.5 |
| GALR1 | EC80 | 581826.67 | 27328.1 | 0.047 | Basal | 101090.00 | 8194.9 | 0.081 | Antagonist | 551760 | 557440 | 554600.00 | 4016.4 | 5.7 |
| GALR2 | EC80 | 170114.29 | 10817 | 0.064 | Basal | 69940.00 | 5031.9 | 0.072 | Antagonist | 135720 | 158320 | 147020.00 | 15980.6 | 23.1 |
| GCGR | EC80 | 273747.50 | 18275.8 | 0.067 | Basal | 31025.00 | 3179.7 | 0.102 | Antagonist | 264640 | 272120 | 268380.00 | 5289.2 | 2.2 |
| GHSR | EC80 | 170132.50 | 13767 | 0.081 | Basal | 44640.00 | 5637.3 | 0.126 | Antagonist | 138440 | 124640 | 131540.00 | 9758.1 | 30.8 |
| GIPR | EC80 | 3466.67 | 276.4 | 0.08 | Basal | 610.00 | 124.2 | 0.204 | Antagonist | 2920 | 2680 | 2800.00 | 169.7 | 23.3 |
| GLP1R | EC80 | 127020.00 | 7078 | 0.056 | Basal | 4890.00 | 877.6 | 0.179 | Antagonist | 55320 | 52520 | 53920.00 | 1979.9 | 59.9 |
| GLP2R | EC80 | 65210.00 | 4474.4 | 0.069 | Basal | 7795.00 | 1195.7 | 0.153 | Antagonist | 58160 | 48880 | 53520.00 | 6562 | 20.4 |
| GPR1 | EC80 | 126495.00 | 8552.7 | 0.068 | Basal | 8530.00 | 962.3 | 0.113 | Antagonist | 105200 | 103680 | 104440.00 | 1074.8 | 18.7 |
| GPR103 | EC80 | 17221.82 | 997.5 | 0.058 | Basal | 6895.00 | 652 | 0.095 | Antagonist | 8880 | 9080 | 8980.00 | 141.4 | 79.8 |
| GPR109A | EC80 | 134956.67 | 8277.4 | 0.061 | Basal | 47385.00 | 2835.4 | 0.06 | Antagonist | 136000 | 124240 | 130120.00 | 8315.6 | 5.5 |
| GPR109B | EC80 | 290945.00 | 33154.7 | 0.114 | Basal | 56205.00 | 6041.6 | 0.107 | Antagonist | 210680 | 201960 | 206320.00 | 6166 | 36.1 |
| GPR119 | EC80 | 68782.86 | 3192.6 | 0.046 | Basal | 41866.67 | 1844.6 | 0.044 | Antagonist | 37520 | 38560 | 38040.00 | 735.4 | 114.2 |
| GPR120 | EC80 | 13500.00 | 1044.4 | 0.077 | Basal | 4545.00 | 371.5 | 0.082 | Antagonist | 5040 | 4600 | 4820.00 | 311.1 | 96.9 |
| GPR35 | EC80 | 78077.50 | 4448.8 | 0.057 | Basal | 12005.00 | 1354.8 | 0.113 | Antagonist | 61000 | 59320 | 60160.00 | 1187.9 | 27.1 |
| GPR92 | EC80 | 198810.91 | 13873.8 | 0.07 | Basal | 75171.43 | 5296.7 | 0.07 | Antagonist | 135600 | 122360 | 128980.00 | 9362.1 | 56.5 |
| GRPR | EC80 | 103252.50 | 5607.7 | 0.054 | Basal | 4740.00 | 206.2 | 0.044 | Antagonist | 94840 | 99360 | 97100.00 | 3196.1 | 6.2 |
| HCRTR1 | EC80 | 270310.00 | 25364.6 | 0.094 | Basal | 12050.00 | 1048 | 0.087 | Antagonist | 149840 | 159080 | 154460.00 | 6533.7 | 44.9 |
| HCRTR2 | EC80 | 352413.33 | 18255.4 | 0.052 | Basal | 7010.00 | 1144.2 | 0.163 | Antagonist | 358760 | 265600 | 312180.00 | 65874.1 | 11.6 |

| | | | | | | | | | | | | | | |
|---|---|---|---|---|---|---|---|---|---|---|---|---|---|---|
| HRH1 | EC80 | 146909.09 | 14478.6 | 0.099 | Basal | 46640.00 | 1679.5 | 0.036 | Antagonist | 157400 | 130360 | 143880.00 | 19120.2 | 3 |
| HRH2 | EC80 | 28931.43 | 1672.5 | 0.058 | Basal | 10060.00 | 1442.2 | 0.143 | Antagonist | 23640 | 25120 | 24380.00 | 1046.5 | 24.1 |
| HRH3 | EC80 | 26307.50 | 1578.9 | 0.06 | Basal | 4285.00 | 237.6 | 0.055 | Antagonist | 25440 | 24960 | 25200.00 | 339.4 | 5 |
| HRH4 | EC80 | 98146.67 | 2505.5 | 0.026 | Basal | 34140.00 | 2253.3 | 0.066 | Antagonist | 75120 | 72440 | 73780.00 | 1895 | 38.1 |
| HTR1A | EC80 | 625890.00 | 35525.3 | 0.057 | Basal | 165885.00 | 16908.1 | 0.102 | Antagonist | 583880 | 528120 | 556000.00 | 39428.3 | 15.2 |
| HTR1B | EC80 | 101560.00 | 3314 | 0.033 | Basal | 43933.33 | 4744.1 | 0.108 | Antagonist | 78440 | 85560 | 82000.00 | 5034.6 | 33.9 |
| HTR1E | EC80 | 12310.00 | 981 | 0.08 | Basal | 4890.00 | 178.6 | 0.037 | Antagonist | 10640 | 10000 | 10320.00 | 452.6 | 26.8 |
| HTR1F | EC80 | 30300.00 | 1828.6 | 0.06 | Basal | 12393.33 | 737.7 | 0.06 | Antagonist | 23640 | 22040 | 22840.00 | 1131.4 | 41.7 |
| HTR2A | EC80 | 182592.00 | 16704.8 | 0.091 | Basal | 35675.00 | 4876.3 | 0.137 | Antagonist | 56600 | 59040 | 57820.00 | 1725.3 | 84.9 |
| HTR2C | EC80 | 134496.00 | 3643.4 | 0.027 | Basal | 73137.14 | 5732.4 | 0.078 | Antagonist | 77040 | 70320 | 73680.00 | 4751.8 | 99.1 |
| HTR5A | EC80 | 200608.57 | 13854.9 | 0.069 | Basal | 64380.00 | 7510.7 | 0.117 | Antagonist | 136640 | 140320 | 138480.00 | 2602.2 | 45.6 |
| KISS1R | EC80 | 25145.00 | 1071.2 | 0.043 | Basal | 4855.00 | 505.9 | 0.104 | Antagonist | 18200 | 19320 | 18760.00 | 792 | 31.5 |
| LHCGR | EC80 | 6504.00 | 519.9 | 0.08 | Basal | 1315.00 | 308.7 | 0.235 | Antagonist | 3440 | 3720 | 3580.00 | 198 | 56.3 |
| LTB4R | EC80 | 122916.67 | 8532.9 | 0.069 | Basal | 16025.00 | 2338.9 | 0.146 | Antagonist | 123120 | 112800 | 117960.00 | 7297.3 | 4.6 |
| MC1R | EC80 | 4008.89 | 301.8 | 0.075 | Basal | 1360.00 | 130 | 0.096 | Antagonist | 3920 | 3560 | 3740.00 | 254.6 | 10.2 |
| MC3R | EC80 | 7412.50 | 415.4 | 0.056 | Basal | 1345.00 | 171 | 0.127 | Antagonist | 5760 | 6680 | 6220.00 | 650.5 | 19.7 |
| MC4R | EC80 | 22310.00 | 1191.2 | 0.053 | Basal | 4540.00 | 380.7 | 0.084 | Antagonist | 14520 | 12640 | 13580.00 | 1329.4 | 49.1 |
| MC5R | EC80 | 19891.43 | 1070.6 | 0.054 | Basal | 9760.00 | 378.6 | 0.039 | Antagonist | 16320 | 15400 | 15860.00 | 650.5 | 39.8 |
| MCHR1 | EC80 | 4094.55 | 365.8 | 0.089 | Basal | 1065.00 | 120.8 | 0.113 | Antagonist | 3680 | 3520 | 3600.00 | 113.1 | 16.3 |
| MCHR2 | EC80 | 48872.50 | 3441.3 | 0.07 | Basal | 8080.00 | 528.1 | 0.065 | Antagonist | 38320 | 42000 | 40160.00 | 2602.2 | 21.4 |
| MLNR | EC80 | 102612.50 | 5553.2 | 0.054 | Basal | 15075.00 | 567.5 | 0.038 | Antagonist | 55520 | 56720 | 56120.00 | 848.5 | 53.1 |
| MRGPRX1 | EC80 | 256573.33 | 17427.5 | 0.068 | Basal | 122771.43 | 8004.4 | 0.065 | Antagonist | 285440 | 284640 | 285040.00 | 565.7 | -21.3 |
| MRGPRX2 | EC80 | 94786.67 | 4840.2 | 0.051 | Basal | 30545.00 | 3060 | 0.1 | Antagonist | 56600 | 49840 | 53220.00 | 4780 | 64.7 |
| MTNR1A | EC80 | 15083.33 | 988.7 | 0.066 | Basal | 5260.00 | 297.8 | 0.057 | Antagonist | 13240 | 12960 | 13100.00 | 198 | 20.2 |
| NMBR | EC80 | 144760.00 | 9565.5 | 0.066 | Basal | 10025.00 | 1086.2 | 0.108 | Antagonist | 84400 | 98240 | 91320.00 | 9786.4 | 39.7 |
| NMU1R | EC80 | 116912.50 | 9622.9 | 0.082 | Basal | 8410.00 | 761 | 0.09 | Antagonist | 80880 | 87720 | 84300.00 | 4836.6 | 30.1 |
| NPBWR1 | EC80 | 18546.67 | 986.2 | 0.053 | Basal | 8154.29 | 345.2 | 0.042 | Antagonist | 19200 | 20400 | 19800.00 | 848.5 | -12.1 |
| NPBWR2 | EC80 | 80557.50 | 3752 | 0.047 | Basal | 9270.00 | 1004.8 | 0.108 | Antagonist | 68160 | 73400 | 70780.00 | 3705.2 | 13.7 |
| NPFFR1 | EC80 | 19692.00 | 1431.6 | 0.073 | Basal | 6606.67 | 686 | 0.104 | Antagonist | 17000 | 15080 | 16040.00 | 1357.6 | 27.9 |
| NPSR1b | EC80 | 41362.50 | 2971.2 | 0.072 | Basal | 6475.00 | 698.2 | 0.108 | Antagonist | 6080 | 5840 | 5960.00 | 169.7 | 101.5 |

| | | | | | | | | | | | | | | |
|---|---|---|---|---|---|---|---|---|---|---|---|---|---|---|
| NPY1R | EC80 | 76812.50 | 5288.8 | 0.069 | Basal | 18835.00 | 1287 | 0.068 | Antagonist | 74280 | 75400 | 74840.00 | 792 | 3.4 |
| NPY2R | EC80 | 414473.33 | 30920.8 | 0.075 | Basal | 23865.00 | 1752.2 | 0.073 | Antagonist | 412160 | 412880 | 412520.00 | 509.1 | 0.5 |
| NTSR1 | EC80 | 143746.67 | 7587.3 | 0.053 | Basal | 44875.00 | 4524.5 | 0.101 | Antagonist | 124400 | 125920 | 125160.00 | 1074.8 | 18.8 |
| OPRD1 | EC80 | 78717.50 | 5825.8 | 0.074 | Basal | 14265.00 | 1372.7 | 0.096 | Antagonist | 72320 | 75880 | 74100.00 | 2517.3 | 7.2 |
| OPRK1 | EC80 | 7227.50 | 456.5 | 0.063 | Basal | 2010.00 | 201.4 | 0.1 | Antagonist | 5920 | 6440 | 6180.00 | 367.7 | 20.1 |
| OPRL1 | EC80 | 155233.33 | 13580.8 | 0.087 | Basal | 32410.00 | 3867.7 | 0.119 | Antagonist | 149480 | 147760 | 148620.00 | 1216.2 | 5.4 |
| OPRM1 | EC80 | 252777.50 | 18791.6 | 0.074 | Basal | 23360.00 | 1591.8 | 0.068 | Antagonist | 270880 | 239240 | 255060.00 | 22372.9 | -1 |
| OXER1 | EC80 | 27008.00 | 2305.1 | 0.085 | Basal | 9925.00 | 1016.3 | 0.102 | Antagonist | 21840 | 26000 | 23920.00 | 2941.6 | 18.1 |
| OXTR | EC80 | 75317.50 | 7372.1 | 0.098 | Basal | 5280.00 | 236.2 | 0.045 | Antagonist | 47400 | 55360 | 51380.00 | 5628.6 | 34.2 |
| P2RY1 | EC80 | 82880.00 | 5677 | 0.068 | Basal | 34880.00 | 2196.3 | 0.063 | Antagonist | 85440 | 75040 | 80240.00 | 7353.9 | 5.5 |
| P2RY11 | EC80 | 49650.00 | 4713.8 | 0.095 | Basal | 5495.00 | 850.9 | 0.155 | Antagonist | 47480 | 44560 | 46020.00 | 2064.8 | 8.2 |
| P2RY12 | EC80 | 77036.67 | 6270.9 | 0.081 | Basal | 17190.00 | 2571.7 | 0.15 | Antagonist | 80160 | 72280 | 76220.00 | 5572 | 1.4 |
| P2RY2 | EC80 | 247240.00 | 7868.1 | 0.032 | Basal | 63825.00 | 2070.8 | 0.032 | Antagonist | 248480 | 249920 | 249200.00 | 1018.2 | -1.1 |
| P2RY4 | EC80 | 141303.33 | 6631.2 | 0.047 | Basal | 49380.00 | 2972 | 0.06 | Antagonist | 84280 | 71840 | 78060.00 | 8796.4 | 68.8 |
| P2RY6 | EC80 | 241502.50 | 14517.2 | 0.06 | Basal | 67900.00 | 14348.5 | 0.211 | Antagonist | 229120 | 249800 | 239460.00 | 14623 | 1.2 |
| PPYR1 | EC80 | 53132.50 | 2678.2 | 0.05 | Basal | 4905.00 | 745.9 | 0.152 | Antagonist | 29800 | 27560 | 28680.00 | 1583.9 | 50.7 |
| PRLHR | EC80 | 9912.50 | 1022.5 | 0.103 | Basal | 1470.00 | 202.6 | 0.138 | Antagonist | 6680 | 5280 | 5980.00 | 990 | 46.6 |
| PROKR1 | EC80 | 67127.50 | 4899.8 | 0.073 | Basal | 6805.00 | 498.9 | 0.073 | Antagonist | 42960 | 45880 | 44420.00 | 2064.8 | 37.6 |
| PROKR2 | EC80 | 7217.50 | 471.5 | 0.065 | Basal | 1595.00 | 105.7 | 0.066 | Antagonist | 5880 | 5120 | 5500.00 | 537.4 | 30.5 |
| PTAFR | EC80 | 448823.33 | 24786 | 0.055 | Basal | 102290.00 | 10183.8 | 0.1 | Antagonist | 409200 | 389680 | 399440.00 | 13802.7 | 14.3 |
| PTGER2 | EC80 | 10680.00 | 973.2 | 0.091 | Basal | 1995.00 | 233.7 | 0.117 | Antagonist | 10920 | 9680 | 10300.00 | 876.8 | 4.4 |
| PTGER3 | EC80 | 131887.50 | 6704.5 | 0.051 | Basal | 47590.00 | 1970.6 | 0.041 | Antagonist | 133560 | 152200 | 142880.00 | 13180.5 | -13 |
| PTGER4 | EC80 | 134923.33 | 9982.5 | 0.074 | Basal | 37895.00 | 2004.2 | 0.053 | Antagonist | 102880 | 96120 | 99500.00 | 4780 | 36.5 |
| PTGFR | EC80 | 16882.50 | 1141.9 | 0.068 | Basal | 830.00 | 186.1 | 0.224 | Antagonist | 9560 | 10360 | 9960.00 | 565.7 | 43.1 |
| PTGIR | EC80 | 83133.33 | 5307.3 | 0.064 | Basal | 33020.00 | 2374.2 | 0.072 | Antagonist | 50120 | 55840 | 52980.00 | 4044.6 | 60.2 |
| PTHR1 | EC80 | 492882.50 | 21421.8 | 0.043 | Basal | 29490.00 | 2194.1 | 0.074 | Antagonist | 394120 | 429040 | 411580.00 | 24692.2 | 17.5 |
| PTHR2 | EC80 | 319283.33 | 22348.8 | 0.07 | Basal | 20530.00 | 2002.7 | 0.098 | Antagonist | 228040 | 229040 | 228540.00 | 707.1 | 30.4 |
| RXFP3 | EC80 | 25547.69 | 1344 | 0.053 | Basal | 8675.00 | 1471.6 | 0.17 | Antagonist | 20840 | 19200 | 20020.00 | 1159.7 | 32.8 |
| SCTR | EC80 | 449982.50 | 23102 | 0.051 | Basal | 72850.00 | 5777.5 | 0.079 | Antagonist | 371480 | 401240 | 386360.00 | 21043.5 | 16.9 |
| SSTR1 | EC80 | 4366.15 | 254 | 0.058 | Basal | 1205.00 | 248.8 | 0.206 | Antagonist | 2880 | 3440 | 3160.00 | 396 | 38.2 |

| | | | | | | | | | | | | | | |
|---|---|---|---|---|---|---|---|---|---|---|---|---|---|---|
| SSTR2 | EC80 | 30282.50 | 2545.1 | 0.084 | Basal | 910.00 | 79.3 | 0.087 | Antagonist | 31160 | 29440 | 30300.00 | 1216.2 | -0.1 |
| SSTR3 | EC80 | 53875.00 | 3870.1 | 0.072 | Basal | 9565.00 | 963.2 | 0.101 | Antagonist | 34560 | 36560 | 35560.00 | 1414.2 | 41.3 |
| SSTR5 | EC80 | 64290.00 | 5738 | 0.089 | Basal | 10955.00 | 865.2 | 0.079 | Antagonist | 63520 | 62480 | 63000.00 | 735.4 | 2.4 |
| TACR1 | EC80 | 329806.67 | 23223 | 0.07 | Basal | 57515.00 | 4295 | 0.075 | Antagonist | 336200 | 320600 | 328400.00 | 11030.9 | 0.5 |
| TACR2 | EC80 | 297392.50 | 19064.6 | 0.064 | Basal | 95200.00 | 5660.5 | 0.059 | Antagonist | 183600 | 171240 | 177420.00 | 8739.8 | 59.3 |
| TACR3 | EC80 | 174465.00 | 8136.6 | 0.047 | Basal | 11005.00 | 884 | 0.08 | Antagonist | 142200 | 156960 | 149580.00 | 10436.9 | 15.2 |
| TBXA2R | EC80 | 88775.00 | 9070 | 0.102 | Basal | 21575.00 | 1586.4 | 0.074 | Antagonist | 90800 | 92680 | 91740.00 | 1329.4 | -4.4 |
| TRHR | EC80 | 18463.33 | 2122.8 | 0.115 | Basal | 1240.00 | 157.1 | 0.127 | Antagonist | 11680 | 10520 | 11100.00 | 820.2 | 42.8 |
| TSHR(L) | EC80 | 7243.33 | 810.6 | 0.112 | Basal | 645.00 | 125.5 | 0.195 | Antagonist | 7480 | 6000 | 6740.00 | 1046.5 | 7.6 |
| UTR2 | EC80 | 16910.00 | 1158.4 | 0.069 | Basal | 6695.00 | 489.4 | 0.073 | Antagonist | 15400 | 14800 | 15100.00 | 424.3 | 17.7 |
| VIPR1 | EC80 | 510297.50 | 27525.9 | 0.054 | Basal | 52490.00 | 1652.2 | 0.031 | Antagonist | 514480 | 532800 | 523640.00 | 12954.2 | -2.9 |
| VIPR2 | EC80 | 356452.50 | 21961.6 | 0.062 | Basal | 14115.00 | 1306.9 | 0.093 | Antagonist | 349080 | 332200 | 340640.00 | 11936 | 4.6 |

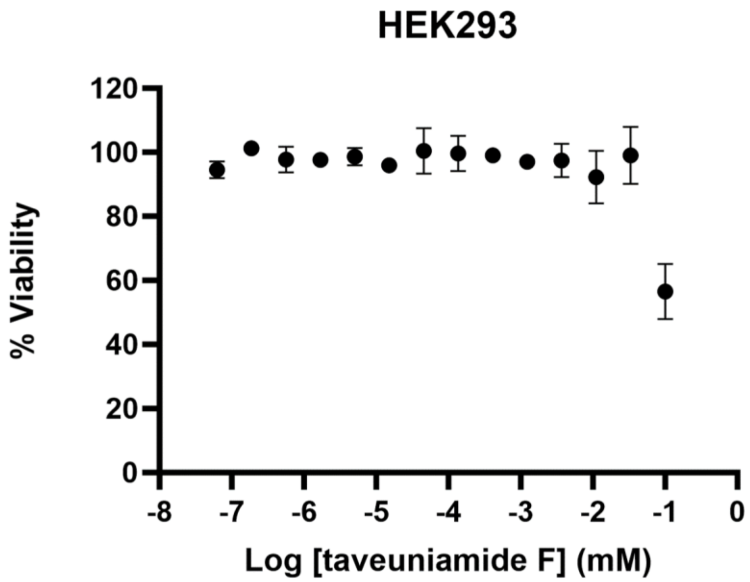


**Figure S26.** Cell viability assay of taveuniamide F using HEK293 cells. The experiment was done as technical triplicates. Data are presented as mean ± SD (n=3).

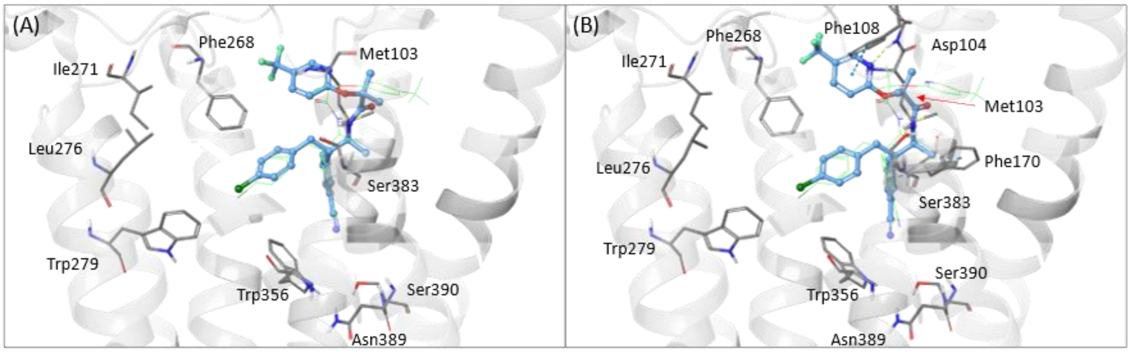


**Figure S27.** Docking poses obtained for taranabant. (A) Glide SP; (B) Induced Fit Docking with GlideSP precision. The determined crystal pose of taranabant (PDBID: 5U09) is overlaid in green lines.

## Supplementary References

(1) Williamson, T. S., Inder.; Gerwick, W. Taveuniamides: new chlorinated toxins from a mixed assemblage of marine cyanobacteria. *Tetrahedron* **2004**, *60* (33), 7025-7033.

(2) Orsini, M. A.; Pannell, L. K.; Erickson, K. L. Polychlorinated acetamides from the cyanobacterium *Microcoleus lyngbyaceus*. *J Nat Prod* **2001**, *64* (5), 572-577.
